# Supplementary material for: Maternal serum levels of prokineticin-1 related to pregnancy complications and metformin use in women with polycystic ovary syndrome: a post hoc analysis of two prospective, randomised, placebo-controlled trials
Source: BMJ Open. 2023 Nov 21;13(11):e073619. doi: 10.1136/bmjopen-2023-073619 (PMC10668301; doi:10.1136/bmjopen-2023-073619)
Supplement: Supplementary data [file bmjopen-2023-073619supp001.pdf]

Supplementary material

All participants in the Pilot and PregMet 1 studies met the Rotterdam criteria (retrospectively evaluated in the Pilot study, as it was conducted prior to the establishment of the Rotterdam criteria), additional inclusion criteria were: age 18-45 years, gestational age between weeks 5-12, and a singleton viable fetus shown on ultrasonography. Exclusion criteria were alanine aminotransferase higher than 90 nmol/L, serum creatinine higher than 139 mmol/L, known alcohol abuse, previously diagnosed diabetes mellitus or fasting serum glucose higher than at inclusion, treatment with oral glucocorticoids, or use of drugs known to interfere with metformin. Randomization to metformin or placebo was stratified according to metformin use at conception. All participants were assigned in a double-blind manner either to metformin or placebo group. The participants received 850 mg (Pilot study) or 1000 mg (PregMet 1 study) metformin or placebo twice daily. Women who used metformin at conception had a "wash-out" period of ≥7 days before inclusion. All the participants received a 1mg tablet of folate daily, and one daily multivitamin tablet containing: vitamin A 800 mg, vitamin B_1 1.4 mg, vitamin B_2 1.6 mg, vitamin B_6 2 mg, vitamin B_{12} 1 mg, folic acid 200 mg, niacin 18 mg, pantotenic acid 6 mg, vitamin C 60 mg, vitamin D 5 mg, vitamin E 10 mg, Fe^{2+} 14 mg, Zn^{+} 15 mg, Cu^{2+} 2 mg, iodine 150 mg, Mn^{2+} 2.5 mg, Cr^{+} 50 mg, and Se^{+} 50 mg (Vitaplex^{W}; Alpharma AS, Norway).

Gestational diabetes mellitus was diagnosed as fasting plasma glucose higher than 7 mmol/L and/or 2-h serum glucose higher than 7.8 mmol/L after an OGTT (75 g glucose dissolved in 300 ml water). Pregnancy-induced hypertension was diagnosed as blood pressure of 140/90 mmHg or higher, measured after 10 minutes rest at least at two occasions. Preeclampsia was diagnosed as blood pressure of 140/90 mmHg or higher, measured on two occasions after pregnancy week 20 and albuminuria of at least 2 dipsticks on one occasion or 1 dipstick on two occasions. Late miscarriage was defined as pregnancy loss between week 13 and week 22 and 6 days, preterm delivery was defined as delivery between week 23 and week 36 and 6 days, including spontaneous deliveries, induced vaginal deliveries, and operative deliveries for medical indications.
