# Supplementary material for: Maternal serum levels of prokineticin-1 related to pregnancy complications and metformin use in women with polycystic ovary syndrome: a post hoc analysis of two prospective, randomised, placebo-controlled trials
Source: BMJ Open. 2023 Nov 21;13(11):e073619. doi: 10.1136/bmjopen-2023-073619 (PMC10668301; doi:10.1136/bmjopen-2023-073619)
Supplement: Supplementary data [file bmjopen-2023-073619supp002.pdf]

Supplementary Table 1 a-e.

a) Baseline characteristics of the participants

	Metformin (n=128)	Placebo (n=136)
Age (years)	29.5 (4.5)	29.0 (4.3)
Weight (kg)	82.3 (18.9)	79.7 (18.7)
BMI (kg/m^2)	29.4 (6.6)	28.7 (7.5)
PCOS phenotype	n=112	n=116
A (PCO+OA+HA) (n, %)	65 (58.0)	67 (57.8)
B (OA+HA) (n, %)	3 (2.7)	4 (3.4)
C (PCO+HA) (n, %)	14 (12.5)	11 (9.5)
D (PCO+OA) (n, %)	30 (26.8)	34 (29.3)
hyperandrogenic (n, %)	82 (73.2)	82 (70.7)
normo-androgenic (n, %)	30 (26.8)	34 (29.3)
Mode of conception	n=128	n=136
Spontaneous (n, %)	76 (59.4)	78 (57.4)
Clomiphene citrate (n, %)	36 (28.1)	28 (20.6)
IVF/ICSI (n, %)	16 (12.5)	26 (19.1)
Other (n, %)	0 (0)	4 (2.9)
Fasting insulin at inclusion (µIU/ml)	15.7 (11.0)	15.3 (10.9)
Fasting glucose at inclusion (mmol/l)	4.6 (0.4)	4.7 (0.6)
HOMA-IR at inclusion	3.3 (2.6)	3.2 (2.3)
2-h glucose (mmol/l)	5.5 (1.5)	5.7 (1.7)
Systolic blood pressure (mm Hg)	119 (12)	117 (12)
Diastolic blood pressure (mm Hg)	75 (13)	73 (10)
Metformin at conception (n, %)	42 (32.8)	41 (30.1)
GDM at inclusion (n, %)	8 (6.3)	10 (7.4)

Abbreviations: BMI = body mass index, PCOS = polycystic ovary syndrome, PCO = polycystic ovaries, OA = oligo- and amennorhea, HA = hyperandrogenism, IVF = *in vitro* fertilization, ICSI = intracytoplasmic sperm injection, HOMA-IR = homeostatic model assessment of insulin resistance, GDM = gestational diabetes mellitus. Continuous variables are presented as mean (SD) and categorical variables as number (%) of participants.

b) Baseline characteristics in the whole population of participants using metformin or no metformin at conception

	Metformin at conception (n=83)	No metformin at conception (n=181)
Age (years)	29.8 (4.3)	29.0 (4.4)
Weight (kg)	82.3 (18.0)	80.3 (19.2)
BMI (kg/m^2)	29.3 (6.6)	28.9 (7.3)
PCOS phenotype	n=77	n=151
A (PCO+OA+HA) (n, %)	45 (58.4)	87 (57.6)
B (OA+HA) (n, %)	3 (3.9)	4 (2.7)
C (PCO+HA) (n, %)	7 (9.1)	18 (11.9)
D (PCO+OA) (n, %)	22 (28.6)	42 (27.8)
hyperandrogenic (n, %)	55 (71.4)	109 (72.2)
normo-androgenic (n, %)	22 (28.6)	42 (27.8)
Mode of conception $^{p=0.003}$	n=83	n=181
Spontaneous (n, %)	48 (57.8)	106 (58.6)
Clomiphene citrate (n, %)	24 (28.9)	40 (22.1)
IVF/ICSI (n, %)	7 (8.4)	35 (19.3)
Other (n, %)	4 (4.8)	0 (0)
Fasting insulin at inclusion (µIU/ml)	15.3 (8.8)	15.7 (11.8)
Fasting glucose at inclusion (mmol/l) $^{p=0.016}$	4.7 (0.6)	4.6 (0.5)
HOMA-IR at inclusion	3.2 (1.9)	3.2 (2.7)
2-h glucose (mmol/l)	5.8 (1.8)	5.5 (1.5)
Systolic blood pressure (mm Hg)	119 (13)	118 (12)
Diastolic blood pressure (mm Hg)	75 (15)	73 (9)
GDM at inclusion (n, %)	10 (13.0)	8 (5.3)

Abbreviations: BMI = body mass index, PCOS = polycystic ovary syndrome, PCO = polycystic ovaries, OA = oligo- and amennorhea, HA = hyperandrogenism, IVF = *in vitro* fertilization, ICSI = intracytoplasmic sperm injection, HOMA-IR = homeostatic model assessment of insulin resistance, GDM = gestational diabetes mellitus. Continuous variables are presented as mean (SD) and categorical variables as number (%) of participants.

c) Baseline characteristics of the participants in the metformin group using metformin or no metformin at conception

	Metformin at conception (n=42)	No metformin at conception (n=86)
Age (years)	30.4 (4.2)	29.1 (4.6)
Weight (kg)	83.3 (18.8)	81.8 (19.1)
BMI (kg/m^2)	29.5 (6.7)	29.3 (6.6)
PCOS phenotype	n=40	n=72
A (PCO+OA+HA) (n, %)	23 (57.5)	42 (58.3)
B (OA+HA) (n, %)	1 (2.5)	2 (2.8)
C (PCO+HA) (n, %)	5 (12.5)	9 (12.5)
D (PCO+OA) (n, %)	11 (27.5)	19 (26.4)
hyperandrogenic (n, %)	29 (72.5)	53 (73.6)
normo-androgenic (n, %)	11 (27.5)	19 (26.4)
Mode of conception $^{p=0.003}$	n=40	n=86
Spontaneous (n, %)	23 (54.8)	53 (61.6)
Clomiphene citrate (n, %)	15 (35.7)	21 (24.4)
IVF/ICSI (n, %)	4 (9.5)	12 (14.0)
Other (n, %)	0 (0)	0 (0)
Fasting insulin at inclusion (µIU/ml)	15.9 (9.6)	15.7 (11.7)
Fasting glucose at inclusion (mmol/l)	4.7 (0.5)	4.6 (0.4)
HOMA-IR at inclusion	3.3 (2.2)	3.2 (2.8)
2-h glucose (mmol/l)	5.6 (1.7)	5.5 (1.5)
Systolic blood pressure (mm Hg)	120 (13)	119 (12)
Diastolic blood pressure (mm Hg)	76 (18)	74 (9)
GDM at inclusion (n, %)	5 (12.5)	3 (4.2)

Abbreviations: BMI = body mass index, PCOS = polycystic ovary syndrome, PCO = polycystic ovaries, OA = oligo- and amennorhea, HA = hyperandrogenism, IVF = *in vitro* fertilization, ICSI = intracytoplasmic sperm injection, HOMA-IR = homeostatic model assessment of insulin resistance, GDM = gestational diabetes mellitus. Continuous variables are presented as mean (SD) and categorical variables as number (%) of participants.

d) Baseline characteristics of the participants using metformin both at conception and during pregnancy or no metformin at all

	Metformin at conception and metformin during pregnancy (n=42)	No metformin at conception and placebo during pregnancy (n=95)
Age (years)	30.4 (4.2)	29.0 (4.3)
Weight (kg)	83.3 (18.8)	79.0 (19.4)
BMI (kg/m^2)	29.5 (6.7)	28.6 (7.9)
PCOS phenotype	n=40	n=79
A (PCO+OA+HA) (n, %)	23 (57.5)	45 (57.0)
B (OA+HA) (n, %)	1 (2.5)	2 (2.5)
C (PCO+HA) (n, %)	5 (12.5)	9 (11.4)
D (PCO+OA) (n, %)	11 (27.5)	23 (29.1)
hyperandrogenic (n, %)	29 (72.5)	56 (70.9)
normo-androgenic (n, %)	11 (27.5)	23 (24.2)
Mode of conception $^{p=0.048}$	n=42	n=95
Spontaneous (n, %)	23 (54.8)	53 (55.8)
Clomiphene citrate (n, %)	15 (35.7)	19 (20.0)
IVF/ICSI (n, %)	4 (9.5)	23 (24.2)
Other (n, %)	0 (0)	0 (0)
Fasting insulin at inclusion (µIU/ml)	15.9 (9.6)	15.7 (12.1)
Fasting glucose at inclusion (mmol/l)	4.7 (0.5)	4.6 (0.5)
HOMA-IR at inclusion	3.3 (2.2)	3.2 (2.5)
2-h glucose (mmol/l)	5.6 (1.7)	5.6 (1.6)
Systolic blood pressure (mm Hg)	120 (13)	117 (12)
Diastolic blood pressure (mm Hg)	76 (18)	72 (9)
GDM at inclusion (n, %)	5 (12.5)	5 (6.3)

Abbreviations: BMI = body mass index, PCOS = polycystic ovary syndrome, PCO = polycystic ovaries, OA = oligo- and amennorhea, HA = hyperandrogenism, IVF = *in vitro* fertilization, ICSI = intracytoplasmic sperm injection, HOMA-IR = homeostatic model assessment of insulin resistance, GDM = gestational diabetes mellitus. Continuous variables are presented as mean (SD) and categorical variables as number (%) of participants.

e) Baseline characteristics of hyperandrogenic and normo-androgenic participants

	Hyperandrogenic PCOS phenotypes (n=164)	Normoandrogenic PCOS phenotypes (n=64)
Age (years)	29.5 (4.6)	28.9 (4.0)
Weight (kg) $^{p=0.034}$	81.8 (18.2)	76.1 (18.2)
BMI (kg/m^{2} $^{p=0.015}$	29.5 (6.9)	27.0 (7.0)
Mode of conception	n=164	n=64
Spontaneous (n, %)	94 (57.4)	32 (50.0)
Clomiphene citrate (n, %)	43 (26.2)	20 (31.2)
IVF/ICSI (n, %)	24 (14.6)	11 (17.2)
Other (n, %)	3 (1.8)	1 (1.6)
Fasting insulin at inclusion (µIU/ml) $^{p=0.029}$	16.5 (12.1)	13.0 (6.7)
Fasting glucose at inclusion (mmol/l)	4.6 (0.5)	4.6 (0.6)
HOMA-IR at inclusion $^{p=0.034}$	3.4 (2.7)	2.7 (1.5)
2-h glucose (mmol/l)	5.6 (1.6)	5.1 (1.3)
Systolic blood pressure (mm Hg)	118 (12)	119 (11)
Diastolic blood pressure (mm Hg)	74 (13)	73 (9)
GDM at inclusion (n, %)	16 (9.8)	5 (6.3)

Abbreviations: BMI = body mass index, IVF = *in vitro* fertilization, ICSI = intracytoplasmic sperm injection, HOMA-IR = homeostatic model assessment of insulin resistance, GDM = gestational diabetes mellitus. Continuous variables are presented as mean (SD) and categorical variables as number (%) of participants.
