# Supplementary material for: Maternal serum levels of prokineticin-1 related to pregnancy complications and metformin use in women with polycystic ovary syndrome: a post hoc analysis of two prospective, randomised, placebo-controlled trials
Source: BMJ Open. 2023 Nov 21;13(11):e073619. doi: 10.1136/bmjopen-2023-073619 (PMC10668301; doi:10.1136/bmjopen-2023-073619)
Supplement: Supplementary data [file bmjopen-2023-073619supp003.pdf]

Supplementary Table 2. Pregnancy complications in women with PCOS according to treatment with metformin or placebo during pregnancy

	Metformin (n=128)	Placebo (n=136)
Pregnancy-induced hypertension (n, %)	7 (5.5)	8 (5.9)
Preeclampsia (n, %)	10 (7.8)	5 (3.7)
Late miscarriage/preterm delivery (n, %)	4 (3.1)	16 (11.8)

Data presented as number (%) of participants.
