# Supplementary material for: Maternal serum levels of prokineticin-1 related to pregnancy complications and metformin use in women with polycystic ovary syndrome: a post hoc analysis of two prospective, randomised, placebo-controlled trials
Source: BMJ Open. 2023 Nov 21;13(11):e073619. doi: 10.1136/bmjopen-2023-073619 (PMC10668301; doi:10.1136/bmjopen-2023-073619)
Supplement: Supplementary data [file bmjopen-2023-073619supp004.pdf]

Supplementary Table 3. Correlations between PROK1 level at pregnancy week 19 and BMI, HOMA-IR, fasting insulin, testosterone and androstenedione levels at inclusion and at pregnancy week 19.

	r-value	p-value	r-value	p-value
	metformin group		placebo group	
BMI at inclusion	-0.091	0.308	-0.013	0.881
BMI at week 19	-0.112	0.207	0.022	0.800
Fasting insulin at inclusion	0.097	0.312	0.017	0.856
Fasting insulin at week 19	0.075	0.432	-0.044	0.636
HOMA-IR at inclusion	0.090	0.345	0.023	0.809
HOMA-IR at week 19	0.085	0.377	-0.065	0.494
Testosterone at inclusion	0.050	0.602	0.119	0.205
Testosterone at week 19	0.123	0.203	-0.057	0.544
Androstenedione at inclusion	0.018	0.850	0.089	0.346
Androstenedione at week 19	0.087	0.308	-0.079	0.881

Abbreviations: BMI = body mass index, HOMA-IR = homeostatic model assessment of insulin resistance
