# Supplementary material for: Maternal serum levels of prokineticin-1 related to pregnancy complications and metformin use in women with polycystic ovary syndrome: a post hoc analysis of two prospective, randomised, placebo-controlled trials
Source: BMJ Open. 2023 Nov 21;13(11):e073619. doi: 10.1136/bmjopen-2023-073619 (PMC10668301; doi:10.1136/bmjopen-2023-073619)
Supplement: Author's manuscript [file bmjopen-2023-073619.draft_revisions.pdf]

BMJ Open

BMJ Open is committed to open peer review. As part of this commitment we make the peer review history of every article we publish publicly available.

When an article is published we post the peer reviewers' comments and the authors' responses online. We also post the versions of the paper that were used during peer review. These are the versions that the peer review comments apply to.

The versions of the paper that follow are the versions that were submitted during the peer review process. They are not the versions of record or the final published versions. They should not be cited or distributed as the published version of this manuscript.

BMJ Open is an open access journal and the full, final, typeset and author-corrected version of record of the manuscript is available on our site with no access controls, subscription charges or pay-per-view fees (http://bmjopen.bmj.com).

If you have any questions on BMJ Open's open peer review process please email info.bmjopen@bmj.com

BMJ Open

Maternal serum levels of prokineticin-1 related to pregnancy complications and metformin use in women with polycystic ovary syndrome

Journal:	*BMJ Open*
Manuscript ID	bmjopen-2023-073619
Article Type:	Original research
Date Submitted by the Author:	14-Mar-2023
Complete List of Authors:	Ujvari, Dorina; Karolinska Institute, Department of Women's and Children's Health; Karolinska Institute, Department of Microbiology<br>Trouva, Anastasia; Karolinska Institute, Department of Molecular Medicine and Surgery; Södersjukhuset, Department of Internal Medicine<br>Linden , Angelica; Karolinska Institute; Karolinska University Hospital, Department of Gynecology and Reproductive Medicine<br>Vanky, Eszter; Norwegian University of Science and Technology; St Olav's University Hospital, Department of Gynaecology and Obstetrics
Keywords:	Maternal medicine < OBSTETRICS, Hypertension < CARDIOLOGY, Reproductive medicine < GYNAECOLOGY

SCHOLARONE™
Manuscripts

I, the Submitting Author has the right to grant and does grant on behalf of all authors of the Work (as defined in the below author licence), an exclusive licence and/or a non-exclusive licence for contributions from authors who are: i) UK Crown employees; ii) where BMJ has agreed a CC-BY licence shall apply, and/or iii) in accordance with the terms applicable for US Federal Government officers or employees acting as part of their official duties; on a worldwide, perpetual, irrevocable, royalty-free basis to BMJ Publishing Group Ltd ("BMJ") its licensees and where the relevant Journal is co-owned by BMJ to the co-owners of the Journal, to publish the Work in this journal and any other BMJ products and to exploit all rights, as set out in our licence.

The Submitting Author accepts and understands that any supply made under these terms is made by BMJ to the Submitting Author unless you are acting as an employee on behalf of your employer or a postgraduate student of an affiliated institution which is paying any applicable article publishing charge ("APC") for Open Access articles. Where the Submitting Author wishes to make the Work available on an Open Access basis (and intends to pay the relevant APC), the terms of reuse of such Open Access shall be governed by a Creative Commons licence – details of these licences and which Creative Commons licence will apply to this Work are set out in our licence referred to above.

Other than as permitted in any relevant BMJ Author's Self Archiving Policies, I confirm this Work has not been accepted for publication elsewhere, is not being considered for publication elsewhere and does not duplicate material already published. I confirm all authors consent to publication of this Work and authorise the granting of this licence.

For peer review only

Maternal serum levels of prokineticin-1 related to pregnancy complications and metformin use in women with polycystic ovary syndrome

Dorina Ujvari[1,2], Anastasia Trouva[3,4], Angelica Lindén Hirschberg[1,5#], Eszter Vanky[6,7#]

[1]Department of Women's and Children's Health, Karolinska Institute, Solna, Sweden

[2]Department of Microbiology, Tumor and Cell Biology, National Pandemic Centre, Centre for Translational Microbiome Research, Karolinska Institute, Solna, Sweden

[3]Department of Molecular Medicine and Surgery, Karolinska Institute, Stockholm, Sweden

[4]Department of Internal Medicine, Section of Diabetes and Endocrinology, Södersjukhuset, Stockholm, Sweden

[5]Department of Gynecology and Reproductive Medicine, Karolinska University Hospital, Solna, Sweden

[6]Department of Clinical and Molecular Medicine, Norwegian University of Science and Technology, Trondheim, Norway

[7]Department of Gynaecology and Obstetrics, St Olav's University Hospital, Trondheim, Norway

[#] These authors contributed equally to the work.

Corresponding author:

Dorina Ujvari, PhD

Department of Women's and Children's Health,

Karolinska Institutet,

SE-171 64 Stockholm, Sweden

E-mail: dorina.ujvari@ki.se

For peer review only

Abstract

Objective

Serum prokineticin-1 (s-PROK1) in the second and third trimester of pregnancy is positively correlated to preeclampsia, intrauterine growth restriction and preterm delivery. Women with polycystic ovary syndrome (PCOS) are prone to these adverse pregnancy outcomes. However, the contribution of PROK1 to the development of pregnancy complications and the effect of metformin and hyperandrogenism on s-PROK1 in PCOS have not been studied previously.

Design

This work is a post-hoc analysis of two prospective, randomized, placebo-controlled trials.

Setting

Pregnant women with PCOS were included from 11 study centres in Norway.

Participants

From 313 women, 264 participated in the present study after exclusions due to dropouts or insufficient serum samples.

Intervention

Women with PCOS were randomly assigned to metformin or placebo, from first trimester to delivery.

Primary and secondary outcome measures

S-PROK1 were analyzed using enzyme-linked immunosorbent assay at gestational week 19 and related to pregnancy complications, fasting insulin levels, HOMA-IR, testosterone, or androstenedione levels, metformin use and hyperandrogenism.

Results

Maternal s-PROK1 in the second trimester did not predict pregnancy-induced hypertension, preeclampsia or late miscarriage/preterm delivery in women with PCOS. However, s-PROK1 was lower in women who used metformin before inclusion, both in those randomized to metformin and to placebo, compared to those who did not. S-PROK1 was also lower in those who used metformin from inclusion, or in those who used metformin both at conception and during pregnancy compared to those who did not use metformin at all. S-PROK1 was lower in hyperandrogenic compared to normo-androgenic women with PCOS.

For peer review only

Conclusions

Maternal s-PROK1 in the second trimester did not predict pregnancy complications in PCOS. Those who used metformin at conception and/or during pregnancy had lower s-PROK1. PCOS women with hyperandrogenism exhibited lower s-PROK1 compared with normo-adrogenic phenotypes.

Trial registration

The clinical trials were registered at www.clinicaltrials.gov as NCT03259919 and NCT00159536.

Keywords: PCOS, pregnancy, prokineticin-1, metformin, preeclampsia, placenta

Abbreviations: PROK1, prokineticin-1; s-PROK1, serum level of prokineticin-1; PCOS, polycystic ovary syndrome

Word count: 1545

For peer review only

Introduction

Polycystic ovary syndrome (PCOS) is the most common endocrine disorder in women of reproductive age. Its prevalence is 10-17% based on at least two of the three features of PCOS (oligo-anovulation, hyperandrogenism and polycystic ovaries) according to the Rotterdam criteria.[1] Approximately 70% of these patients have insulin resistance and compensatory hyperinsulinemia.[2] PCOS is closely related to subfertility and pregnancy complications, including miscarriage, gestational diabetes, preeclampsia, intrauterine growth restriction (IUGR) and preterm delivery.[3, 4] Hyperinsulinemia and hyperandrogenism might contribute to the development of these complications.[5, 6]

Prokineticin-1 (PROK1), is a crucial regulator of embryo implantation and placental development.[7, 8] It is expressed in the endometrium, ovaries, adrenal glands and placenta.[9, 10] The placenta is thought to be the main source of PROK1 in the maternal circulation.[11, 12] In uncomplicated pregnancies, serum level of PROK1 (s-PROK1) increases 5-fold in the first trimester compared to non-pregnant state,[13] and promotes the proliferation of anchoring cytotrophoblasts and formation of trophoblastic plugs in the maternal spiral arteries.[11, 14] After gestational week 10-12, PROK1 levels decline, facilitating the transformation of trophoblasts into an invasive phenotype, the remodeling of the maternal spiral arteries and the increase of placental oxygen tension to promote placental angiogenesis in order to supply the growing fetus.[11, 13] Dysregulation of PROK1 is associated with preeclampsia, IUGR and preterm delivery.[13-16] Despite an increased occurrence of these pregnancy complications, the role of PROK1 has not been studied in PCOS.

The administration of metformin, an insulin sensitizer, in the treatment of non-pregnant PCOS women is well established.[17] Evidence shows its usefulness in reducing late miscarriages and preterm deliveries, however, its efficacy on other complications is debated and the mechanism of action is not fully understood.[18-21]

The primary objective of this study was to investigate if s-PROK1 predicts pregnancy-induced hypertension, preeclampsia, and late miscarriage/preterm delivery. We

For peer review only

hypothesized that metformin treatment was associated with lower, and hyperandrogenism with higher s-PROK1 in the second trimester.

Materials and methods

Study design

This work is a post-hoc analysis of two prospective, randomized, placebo-controlled trials, investigating the effect of metformin on pregnancy complications in women with PCOS. 264 women participated with analyzed serum samples in the present study, 128 and 136 in the metformin and placebo group, respectively (Figure 1). The clinical studies[18, 19] are described in the Supplementary material.

Enzyme-linked immunosorbent assay

Serum samples, collected at gestational week 19, were analyzed by a PROK1 ELISA kit (Thermo Fischer Scientific), in 1:4 dilution. Absorbance of the samples was measured in duplicates at 450 and 550 nm on a Multiscan FC photometer, and A_{450}-A_{550} values were used to correct for optical imperfections in the microplate. To plot the standard curves, the four-parameter logistic curve fit was applied. The limit of detection (LOD) was 13.72 pg/ml. Values below the detection limit were replaced by 0.5xLOD. The intra-assay and inter-assay coefficients of variations were <10% and <12%, respectively.

Statistical analysis

Statistical procedures were performed using SPSS by IBM Version 25. Differences in baseline characteristics were analyzed using unpaired T-test and Fischer's exact test. S-PROK1 was positively skewed and therefore log-transformed. Logistic regression analysis was used to analyze whether s-PROK1 predicts pregnancy complications. Comparisons of s-PROK1 between subgroups were analyzed by Mann-Whitney U-test. Correlations between s-PROK1 and other variables were evaluated using Spearman`s rank order correlation test. $P<0.05$ was considered as statistically significant.

Ethics statement

For peer review only - http://bmjopen.bmj.com/site/about/guidelines.xhtml

An informed consent was obtained from each patient and the declaration of Helsinki was followed throughout the studies. The Committee for Medical Research Ethics of Health Region IV, Norway and The Norwegian Medicines Agency approved the studies (2010/3115 (145-04)-27/10/2016). The clinical trials were registered at www.clinicaltrials.gov as NCT03259919 (The pilot study) and NCT00159536 (The PregMet 1 study).

Results

Study population

Baseline characteristics of the women in the metformin and the placebo groups were comparable (Supplementary Table 1). Baseline characteristics were compared in additional subgroups (Supplementary Table 1). Weight, body mass index (BMI), fasting insulin levels and homeostatic model assessment for insulin resistance (HOMA-IR) were higher in hyperandrogenic compared to normo-androgenic participants ($p<0.05$).

Pregnancy complications

Pregnancy complications are listed in Supplementary Table 2. S-PROK1 did not predict pregnancy-induced hypertension (n=15) (OR 0.95, 95% CI 0.32–2.74, p=0.92), preeclampsia (n=15) (OR 0.47, 95% CI 0.14–1.35, p=0.17) or late miscarriage/preterm delivery (n=20) (OR 0.62, 95% CI 0.24–1.56, p=0.32).

PROK1 and metformin use at conception, during pregnancy, and both at conception and during pregnancy

S-PROK1 was lower in women who used metformin at conception compared to those who did not, independent of later randomization to metformin or placebo ($p<0.05$) (Figure 2a). S-PROK1 was not different in women randomized to metformin or placebo during pregnancy (Figure 2b). Furthermore, s-PROK1 was lower in women who used metformin both at conception and during pregnancy, compared to those who used metformin during pregnancy ($p<0.05$) (Figure 2c). S-PROK1 was lower in women who

For peer review only

took metformin both at conception and during pregnancy compared to those who did not use metformin at all ($p<0.01$) (Figure 2d).

PCOS phenotypes

S-PROK1 was lower in hyperandrogenic compared to normo-androgenic women ($p<0.05$) (Figure 3).

Correlations

S-PROK1 showed no correlation with BMI, fasting insulin levels, HOMA-IR, testosterone, or androstenedione levels either at inclusion or at week 19 of gestation (Supplementary Table 3).

Discussion

The primary aim of this study was to investigate if s-PROK1 predicts pregnancy-induced hypertension, preeclampsia, and late miscarriage/preterm delivery in women with PCOS. Previously, s-PROK1 was reported to be higher in the second and third trimesters in preeclamptic and IUGR pregnancies compared to uncomplicated pregnancies.[13, 14] We and others have also demonstrated that PROK1 restricts the migration and invasion of trophoblast cells *in vitro*, suggesting that elevated s-PROK1 impairs embryo implantation and placentation.[13, 22] However, s-PROK1 in the second trimester did not predict pregnancy-induced hypertension or preeclampsia in our women with PCOS. It has also been demonstrated that PROK1 is up-regulated in the myometrium and the placenta during labor, suggesting a role for PROK1 in the induction of term and preterm labor.[15, 16] However, in this study, s-PROK1 did not predict late miscarriages/preterm deliveries. Thus, we could not confirm the association of s-PROK1 with pregnancy complications in PCOS as it was demonstrated in non-PCOS women. However, we cannot exclude that a limited number of cases might have influenced our results.

We also investigated the impact of metformin on s-PROK1 and found lower levels by metformin use at conception, both with and without metformin use after inclusion, suggesting that metformin intake, especially in early pregnancy, might have a lowering

For peer review only - http://bmjopen.bmj.com/site/about/guidelines.xhtml

effect on s-PROK1. However, participants were not randomized at this timepoint and the lower s-PROK1 at week 19 might reflect that those who used metformin at conception differed from those who did not. It is known that metformin decreases insulin levels, although the treatment does not seem to prevent gestational diabetes in PCOS women.[18, 19, 21] We recently demonstrated that insulin upregulates PROK1 in decidualizing endometrial stromal cells *in vitro*.[22] However, in this study, we found no correlation between s-PROK1 and insulin levels.

A possible association between s-PROK1 and hyperandrogenism was also investigated. In contrast to our hypothesis, s-PROK1 was lower in women with hyperandrogenic phenotypes compared to normo-androgenic phenotype. However, there was no correlation between s-PROK1 and androgen levels at inclusion and week 19. Direct and indirect evidence support the role of androgens in the pathogenesis of placenta-related pregnancy complications.[23] We have previously showed that androgens potentiate insulin to up-regulate PROK1 in decidualizing endometrial stromal cells *in vitro*.[24] Increased maternal serum androgens have been reported in preeclamptic pregnancies.[25-28] PCOS women with pregnancy complications have higher androgen levels than those without.[29] It was also shown that trophoblast invasion and placentation are impaired in PCOS compared to controls, and this impairment is associated with insulin resistance and circulating testosterone concentrations.[30] However, androgens do not seem to directly contribute to the regulation of s-PROK1, although hyperandrogenism could be indirectly involved.

For peer review only

Conclusions

S-PROK1, in the second trimester, did not predict pregnancy-induced hypertension, preeclampsia, or late miscarriage/preterm delivery in pregnant women with PCOS. Women who used metformin at conception and/or during pregnancy and those with hyperandrogenism exhibited lower s-PROK1. Further studies are needed to determine the role of s-PROK1 in pregnancy complications and the involvement of metformin and hyperandrogenism in the regulation of PROK1 in PCOS.

Article summary

For peer review only - http://bmjopen.bmj.com/site/about/guidelines.xhtml

Strengths and limitations of this study

There are several strengths of the present study including the well-characterized women with PCOS and the randomized, double-blinded placebo-controlled multi-center study design, prospective data sampling and meticulous quality-checked diagnose control. Some limitations should also be mentioned. Since maternal s-PROK1 has a dynamic course during pregnancy, timing of blood sampling for determination of s-PROK1 is crucial. Based on previous studies showing elevated s-PROK1 in the second and third trimester of preeclamptic and IUGR pregnancies, we chose to analyze s-PROK1 in gestational week 19. However, we cannot exclude that s-PROK1 should have been determined at several timepoints to study the course of PROK1 for pregnancy complications in PCOS. Furthermore, the number of pregnancy complication cases was limited possibly leading to type II errors.

Acknowledgements

The authors thank Weifa A/S for delivering metformin and placebo tablets free of charge.

Author contributions

EV conceived and designed the original protocol. On the initiative of DU and ALH, the protocol of the present study was written together with EV. DU run the analysis, DU and AT managed the database and analyzed the data with the input from EV and ALH. A statistician performed the statistical analyses. DU and AT drafted the manuscript with input from ALH, EV. All the authors contributed to the article, reviewed and approved the submitted version.

Conflicts of interest

The authors declare no conflicts of interest.

Funding information

For peer review only - http://bmjopen.bmj.com/site/about/guidelines.xhtml

This work was supported by grants from the Liaison Committee between the Central Norway Regional Health Authority and the Norwegian University of Science and Technology, Swedish Research Council (2021-01348), Stockholm County Council (2019-0248) and Åke Wiberg foundation (M18-0114).

Data availability

Data are available upon reasonable request.

For peer review only

References

1. Bozdag G, Mumusoglu S, Zengin D, Karabulut E, Yildiz BO. The prevalence and phenotypic features of polycystic ovary syndrome: a systematic review and meta-analysis. Hum Reprod. 2016;31(12):2841-55.
2. March WA, Moore VM, Willson KJ, Phillips DI, Norman RJ, Davies MJ. The prevalence of polycystic ovary syndrome in a community sample assessed under contrasting diagnostic criteria. Hum Reprod. 2010;25(2):544-51.
3. Hirschberg AL. Polycystic ovary syndrome, obesity and reproductive implications. Womens Health (Lond). 2009;5(5):529-40; quiz 41-2.
4. Franks S. Polycystic ovary syndrome. N Engl J Med. 1995;333(13):853-61.
5. Azziz R, Carmina E, Chen Z, Dunaif A, Laven JS, Legro RS, et al. Polycystic ovary syndrome. Nat Rev Dis Primers. 2016;2:16057.
6. Dumesic DA, Oberfield SE, Stener-Victorin E, Marshall JC, Laven JS, Legro RS. Scientific Statement on the Diagnostic Criteria, Epidemiology, Pathophysiology, and Molecular Genetics of Polycystic Ovary Syndrome. Endocr Rev. 2015;36(5):487-525.
7. Alfaidy N, Brouillet S, Rajaraman G, Kalionis B, Hoffmann P, Barjat T, et al. The Emerging Role of the Prokineticins and Homeobox Genes in the Vascularization of the Placenta: Physiological and Pathological Aspects. Front Physiol. 2020;11:591850.
8. Alfaidy N, Hoffmann P, Boufettal H, Samouh N, Aboussaouira T, Benharouga M, et al. The multiple roles of EG-VEGF/PROK1 in normal and pathological placental angiogenesis. Biomed Res Int. 2014;2014:451906.
9. Battersby S, Critchley HO, Morgan K, Millar RP, Jabbour HN. Expression and regulation of the prokineticins (endocrine gland-derived vascular endothelial growth factor and Bv8) and their receptors in the human endometrium across the menstrual cycle. The Journal of clinical endocrinology and metabolism. 2004;89(5):2463-9.
10. Traboulsi W, Brouillet S, Sergent F, Boufettal H, Samouh N, Aboussaouira T, et al. Prokineticins in central and peripheral control of human reproduction. Horm Mol Biol Clin Investig. 2015;24(2):73-81.
11. Hoffmann P, Feige JJ, Alfaidy N. Expression and oxygen regulation of endocrine gland-derived vascular endothelial growth factor/prokineticin-1 and

its receptors in human placenta during early pregnancy. Endocrinology. 2006;147(4):1675-84.
12. Brouillet S, Hoffmann P, Benharouga M, Salomon A, Schaal JP, Feige JJ, et al. Molecular characterization of EG-VEGF-mediated angiogenesis: differential effects on microvascular and macrovascular endothelial cells. Molecular biology of the cell. 2010;21(16):2832-43.
13. Hoffmann P, Saoudi Y, Benharouga M, Graham CH, Schaal JP, Mazouni C, et al. Role of EG-VEGF in human placentation: Physiological and pathological implications. Journal of cellular and molecular medicine. 2009;13(8B):2224-35.
14. Brouillet S, Murthi P, Hoffmann P, Salomon A, Sergent F, De Mazancourt P, et al. EG-VEGF controls placental growth and survival in normal and pathological pregnancies: case of fetal growth restriction (FGR). Cellular and molecular life sciences : CMLS. 2013;70(3):511-25.
15. Catalano RD, Lannagan TR, Gorowiec M, Denison FC, Norman JE, Jabbour HN. Prokineticins: novel mediators of inflammatory and contractile pathways at parturition? Mol Hum Reprod. 2010;16(5):311-9.
16. Gorowiec MR, Catalano RD, Norman JE, Denison FC, Jabbour HN. Prokineticin 1 induces inflammatory response in human myometrium: a potential role in initiating term and preterm parturition. Am J Pathol. 2011;179(6):2709-19.
17. Teede HJ, Misso ML, Costello MF, Dokras A, Laven J, Moran L, et al. Recommendations from the international evidence-based guideline for the assessment and management of polycystic ovary syndrome. Hum Reprod. 2018;33(9):1602-18.
18. Vanky E, Salvesen KA, Heimstad R, Fougner KJ, Romundstad P, Carlsen SM. Metformin reduces pregnancy complications without affecting androgen levels in pregnant polycystic ovary syndrome women: results of a randomized study. Hum Reprod. 2004;19(8):1734-40.
19. Vanky E, Stridsklev S, Heimstad R, Romundstad P, Skogoy K, Kleggetveit O, et al. Metformin versus placebo from first trimester to delivery in polycystic ovary syndrome: a randomized, controlled multicenter study. The Journal of clinical endocrinology and metabolism. 2010;95(12):E448-55.
20. Fornes R, Simin J, Nguyen MH, Cruz G, Crisosto N, van der Schaaf M, et al. Pregnancy, perinatal and childhood outcomes in women with and without polycystic ovary syndrome and metformin during pregnancy: a nationwide population-based study. Reprod Biol Endocrinol. 2022;20(1):30.
21. Lovvik TS, Carlsen SM, Salvesen O, Steffensen B, Bixo M, Gomez-Real F, et al. Use of metformin to treat pregnant women with polycystic ovary syndrome (PregMet2): a randomised, double-blind, placebo-controlled trial. Lancet Diabetes Endocrinol. 2019;7(4):256-66.
22. Ujvari D, Jakson I, Oldmark C, Attarha S, Alkasalias T, Salamon D, et al. Prokineticin 1 is up-regulated by insulin in decidualizing human endometrial stromal cells. Journal of cellular and molecular medicine. 2018;22(1):163-72.
23. Kumar S, Gordon GH, Abbott DH, Mishra JS. Androgens in maternal vascular and placental function: implications for preeclampsia pathogenesis. Reproduction. 2018;156(5):R155-R67.
24. Ujvari D, Graells Brugalla C, Hirschberg AL. Dihydrotestosterone potentiates insulin to up-regulate prokineticin-1 in decidualizing human

For peer review only - http://bmjopen.bmj.com/site/about/guidelines.xhtml

endometrial stromal cells. Journal of cellular and molecular medicine. 2020;24(5):3242-5.
25. Acromite MT, Mantzoros CS, Leach RE, Hurwitz J, Dorey LG. Androgens in preeclampsia. American journal of obstetrics and gynecology. 1999;180(1 Pt 1):60-3.
26. Serin IS, Kula M, Basbug M, Unluhizarci K, Gucer S, Tayyar M. Androgen levels of preeclamptic patients in the third trimester of pregnancy and six weeks after delivery. Acta obstetricia et gynecologica Scandinavica. 2001;80(11):1009-13.
27. Atamer Y, Erden AC, Demir B, Kocyigit Y, Atamer A. The relationship between plasma levels of leptin and androgen in healthy and preeclamptic pregnant women. Acta obstetricia et gynecologica Scandinavica. 2004;83(5):425-30.
28. Carlsen SM, Romundstad P, Jacobsen G. Early second-trimester maternal hyperandrogenemia and subsequent preeclampsia: a prospective study. Acta obstetricia et gynecologica Scandinavica. 2005;84(2):117-21.
29. Falbo A, Rocca M, Russo T, D'Ettore A, Tolino A, Zullo F, et al. Changes in androgens and insulin sensitivity indexes throughout pregnancy in women with polycystic ovary syndrome (PCOS): relationships with adverse outcomes. Journal of ovarian research. 2010;3:23.
30. Palomba S, Russo T, Falbo A, Di Cello A, Amendola G, Mazza R, et al. Decidual endovascular trophoblast invasion in women with polycystic ovary syndrome: an experimental case-control study. The Journal of clinical endocrinology and metabolism. 2012;97(7):2441-9.

For peer review only

Figure legends

Figure 1

Flowchart of the original and the secondary studies.

Figure 2

a. Serum levels of PROK1 at pregnancy week 19 in women with PCOS, who took metformin at conception compared to those, who did not use metformin at conception. Values are presented as median, interquartile ranges (P25-P75) and non-outlier ranges (min-max). Open circles represent outliers (1.5*the box-height from the box) and the stars represent extremes (3*the box-height from the box).

b. Serum levels of PROK1 at pregnancy week 19 in women with PCOS, who took metformin compared to those, who took placebo after randomization. Values are presented as median, interquartile ranges (P25-P75) and non-outlier ranges (min-max). Open circles represent outliers (1.5*the box-height from the box) and the stars represent extremes (3*the box-height from the box).

c. Serum levels of PROK1 at pregnancy week 19 in women with PCOS, who took metformin both at conception and after randomization compared to those, who did not use metformin at conception, but used after randomization. Values are presented as median, interquartile ranges (P25-P75) and non-outlier ranges (min-max). Open circles represent outliers (1.5*the box-height from the box) and the stars represent extremes (3*the box-height from the box).

d. Serum levels of PROK1 at pregnancy week 19 in women with PCOS, who took metformin both at conception and after randomization compared to those, who did not use metformin either at conception or after randomization. Values are presented as median, interquartile ranges (P25-P75) and non-outlier ranges (min-max). Open circles represent outliers (1.5*the box-height from the box) and the stars represent extremes (3*the box-height from the box).

Figure 3

For peer review only - http://bmjopen.bmj.com/site/about/guidelines.xhtml

Serum levels of PROK1 at pregnancy week 19 in women with PCOS and hyperandrogenism compared with those without hyperandrogenism. Values are presented as median, interquartile ranges (P25-P75) and non-outlier ranges (min-max). Open circles represent outliers (1.5*the box-height from the box).

Supplementary material

Description and details of the two prospective, randomized, placebo-controlled trials

For peer review only

Figure 1

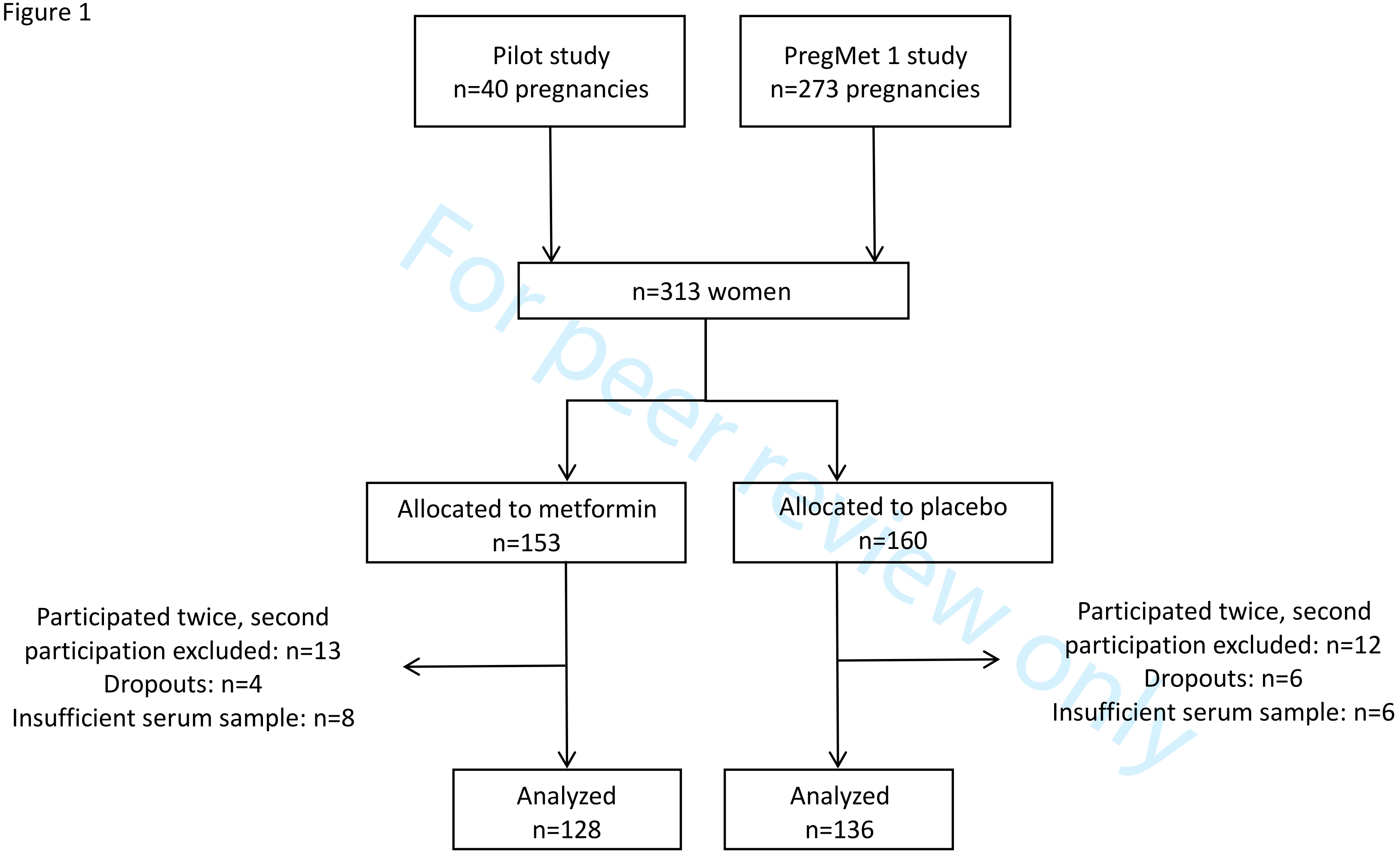

For peer review only - http://bmjopen.bmj.com/site/about/guidelines.xhtml

Figure 2

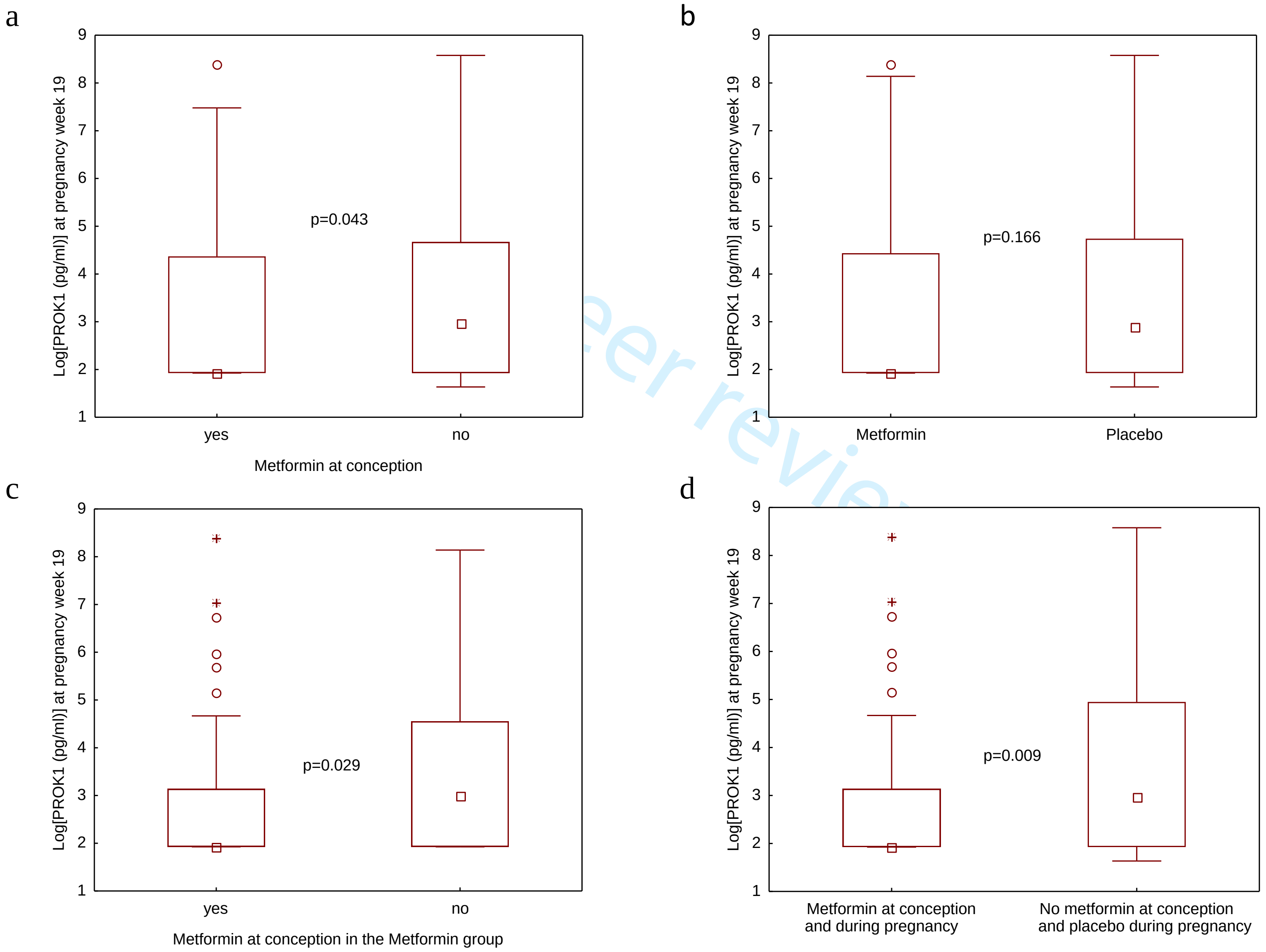
a
b
c
d
Log[PROK1 (pg/ml)] at pregnancy week 19
p=0.043
yes
no
Metformin at conception
p=0.166
Metformin
Placebo
p=0.029
Metformin at conception in the Metformin group
p=0.009
Metformin at conception and during pregnancy
No metformin at conception and placebo during pregnancy

Figure 3

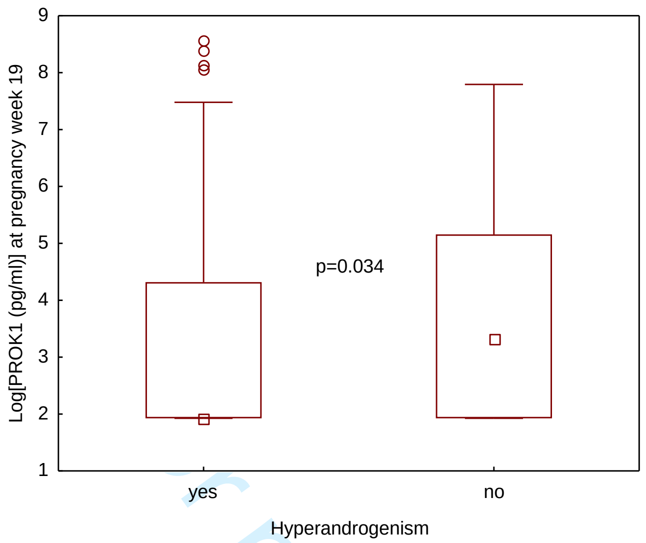

For peer review only

Supplementary material

All participants in the Pilot and PregMet 1 studies met the Rotterdam criteria (retrospectively evaluated in the Pilot study, as it was conducted prior to the establishment of the Rotterdam criteria), additional inclusion criteria were: age 18-45 years, gestational age between weeks 5-12, and a singleton viable fetus shown on ultrasonography. Exclusion criteria were alanine aminotransferase higher than 90 nmol/L, serum creatinine higher than 139 mmol/L, known alcohol abuse, previously diagnosed diabetes mellitus or fasting serum glucose higher than at inclusion, treatment with oral glucocorticoids, or use of drugs known to interfere with metformin. Randomization to metformin or placebo was stratified according to metformin use at conception. All participants were assigned in a double-blind manner either to metformin or placebo group. The participants received 850 mg (Pilot study) or 1000 mg (PregMet 1 study) metformin or placebo twice daily. Women who used metformin at conception had a "wash-out" period of ≥7 days before inclusion. All the participants received a 1mg tablet of folate daily, and one daily multivitamin tablet containing: vitamin A 800 mg, vitamin B_1 1.4 mg, vitamin B_2 1.6 mg, vitamin B_6 2 mg, vitamin B_{12} 1 mg, folic acid 200 mg, niacin 18 mg, pantotenic acid 6 mg, vitamin C 60 mg, vitamin D 5 mg, vitamin E 10 mg, Fe^{2+} 14 mg, Zn^{+} 15 mg, Cu^{2+} 2 mg, iodine 150 mg, Mn^{2+} 2.5 mg, Cr^{+} 50 mg, and Se^{+} 50 mg (Vitaplex^{W}; Alpharma AS, Norway).

Gestational diabetes mellitus was diagnosed as fasting plasma glucose higher than 7 mmol/L and/or 2-h serum glucose higher than 7.8 mmol/L after an OGTT (75 g glucose dissolved in 300 ml water). Pregnancy-induced hypertension was diagnosed as blood pressure of 140/90 mmHg or higher, measured after 10 minutes rest at least at two occasions. Preeclampsia was diagnosed as blood pressure of 140/90 mmHg or higher, measured on two occasions after pregnancy week 20 and albuminuria of at least 2 dipsticks on one occasion or 1 dipstick on two occasions. Late miscarriage was defined as pregnancy loss between week 13 and week 22 and 6 days, preterm delivery was defined as delivery between week 23 and week 36 and 6 days, including spontaneous deliveries, induced vaginal deliveries, and operative deliveries for medical indications.

For peer review only

Supplementary Table 1 a-e.

a) Baseline characteristics of the participants

	Metformin (n=128)	Placebo (n=136)
Age (years)	29.5 (4.5)	29.0 (4.3)
Weight (kg)	82.3 (18.9)	79.7 (18.7)
BMI (kg/m^2)	29.4 (6.6)	28.7 (7.5)
PCOS phenotype	(n=112)	(n=116)
hyperandrogenic (n, %)	82 (73.2)	82 (70.7)
normo-androgenic (n, %)	30 (26.8)	34 (29.3)
Fasting insulin at inclusion (µIU/ml)	15.7 (11.0)	15.3 (10.9)
Fasting glucose at inclusion (mmol/l)	4.6 (0.4)	4.7 (0.6)
HOMA-IR at inclusion	3.3 (2.6)	3.2 (2.3)
2-h glucose (mmol/l)	5.5 (1.5)	5.7 (1.7)
Systolic blood pressure (mm Hg)	119 (12)	117 (12)
Diastolic blood pressure (mm Hg)	75 (13)	73 (10)
Metformin at conception (n, %)	42 (32.8)	41 (30.1)
GDM at inclusion (n, %)	8 (6.3)	10 (7.4)

Abbreviations: BMI = body mass index, GDM = gestational diabetes mellitus, HOMA-IR = homeostatic model assessment of insulin resistance, PCOS = polycystic ovary syndrome

Continuous variables are presented as mean (SD) and categorical variables as number (%) of participants.

b) Baseline characteristics in the whole population of participants using metformin or no metformin at conception

	Metformin at conception (n=83)	No metformin at conception (n=181)
Age (years)	29.8 (4.3)	29.0 (4.4)
Weight (kg)	82.3 (18.0)	80.3 (19.2)
BMI (kg/m^2)	29.3 (6.6)	28.9 (7.3)
PCOS phenotype	(n=40)	(n=72)
hyperandrogenic (n, %)	55 (71.4)	109 (72.2)
normo-androgenic (n, %)	22 (28.6)	42 (27.8)
Fasting insulin at inclusion (µIU/ml)	15.3 (8.8)	15.7 (11.8)
Fasting glucose at inclusion (mmol/l)	4.7 (0.6)	4.6 (0.5)*
2-h glucose (mmol/l)	5.8 (1.8)	5.5 (1.5)
HOMA-IR at inclusion	3.2 (1.9)	3.2 (2.7)
Systolic blood pressure (mm Hg)	119 (13)	118 (12)
Diastolic blood pressure (mm Hg)	75 (15)	73 (9)
GDM at inclusion (n, %)	10 (13.0)	8 (5.3)

Abbreviations: BMI = body mass index, GDM = gestational diabetes mellitus, HOMA-IR = homeostatic model assessment of insulin resistance, PCOS =

For peer review only

polycystic ovary syndrome. Continuous variables are presented as mean (SD) and categorical variables as number (%) of participants. *p<0.05

c) Baseline characteristics of the participants in the metformin group using metformin or no metformin at conception

	Metformin at conception (n=42)	No metformin at conception (n=86)
Age (years)	30.4 (4.2)	29.1 (4.6)
Weight (kg)	83.3 (18.8)	81.8 (19.1)
BMI (kg/m^2)	29.5 (6.7)	29.3 (6.6)
PCOS phenotype	(n=40)	(n=72)
hyperandrogenic (n, %)	29 (72.5)	53 (73.6)
normo-androgenic (n, %)	11 (27.5)	19 (26.4)
Fasting insulin at inclusion (μIU/ml)	15.9 (9.6)	15.7 (11.7)
Fasting glucose at inclusion (mmol/l)	4.7 (0.5)	4.6 (0.4)
2-h glucose (mmol/l)	5.6 (1.7)	5.5 (1.5)
HOMA-IR at inclusion	3.3 (2.2)	3.2 (2.8)
Systolic blood pressure (mm Hg)	120 (13)	119 (12)
Diastolic blood pressure (mm Hg)	76 (18)	74 (9)
GDM at inclusion (n, %)	5 (12.5)	3 (4.2)

Abbreviations: BMI = body mass index, GDM = gestational diabetes mellitus, HOMA-IR = homeostatic model assessment of insulin resistance, PCOS = polycystic ovary syndrome. Continuous variables are presented as mean (SD) and categorical variables as number (%) of participants. *p<0.05

d) Baseline characteristics of the participants using metformin both at conception and during pregnancy or no metformin at all

Characteristic	Metformin at conception and metformin during pregnancy (n=42)	No metformin at conception and placebo during pregnancy (n=95)
Age (years)	30.4 (4.2)	29.0 (4.3)
Weight (kg)	83.3 (18.8)	79.0 (19.4)
BMI (kg/m^2)	29.5 (6.7)	28.6 (7.9)
PCOS phenotype	(n=40)	(n=72)
hyperandrogenic (n, %)	29 (72.5)	56 (70.9)
normo-androgenic (n, %)	11 (27.5)	23 (29.1)
Fasting insulin at inclusion (μIU/ml)	15.9 (9.6)	15.7 (12.1)
Fasting glucose at inclusion (mmol/l)	4.7 (0.6)	4.6 (0.5)
2-h glucose (mmol/l)	5.6 (1.7)	5.6 (1.6)
HOMA-IR at inclusion	3.3 (2.2)	3.2 (2.5)
Systolic blood pressure (mm Hg)	120 (13)	117 (12)
Diastolic blood pressure (mm Hg)	76 (18)	72 (9)
GDM at inclusion (n, %)	5 (12.5)	5 (6.3)

For peer review only

Abbreviations: BMI = body mass index, GDM = gestational diabetes mellitus, HOMA-IR = homeostatic model assessment of insulin resistance, PCOS = polycystic ovary syndrome. Continuous variables are presented as mean (SD) and categorical variables as number (%) of participants.

e) Baseline characteristics of hyperandrogenic and normo-androgenic participants

Characteristic	Hyperandrogenic PCOS phenotypes (n=164)	Normo-androgenic PCOS phenotypes (n=64)
Age (years)	29.5 (4.6)	28.9 (4.0)
Weight (kg)	81.8 (18.2)	76.1 (18.2)*
BMI (kg/m^2)	29.5 (6.9)	27.0 (7.0)*
Fasting insulin at inclusion (µIU/ml)	16.5 (12.1)	13.0 (6.7)*
Fasting glucose at inclusion (mmol/l)	4.6 (0.5)	4.6 (0.6)
2-h glucose (mmol/l)	5.6 (1.6)	5.1 (1.3)
HOMA-IR at inclusion	3.4 (2.7)	2.7 (1.5)*
Systolic blood pressure (mm Hg)	118 (12)	119 (11)
Diastolic blood pressure (mm Hg)	74 (13)	73 (9)
GDM at inclusion (n, %)	16 (9.8)	5 (3.1)

Abbreviations: BMI = body mass index, GDM = gestational diabetes mellitus, HOMA-IR = homeostatic model assessment of insulin resistance, PCOS = polycystic ovary syndrome. Continuous variables are presented as mean (SD) and categorical variables as number (%) of participants. *$p<0.05$

For peer review only

Supplementary Table 2. Pregnancy complications in women with PCOS according to treatment with metformin or placebo during pregnancy

	Metformin (n=128)	Placebo (n=136)
Pregnancy-induced hypertension (n, %)	7 (5.5)	8 (5.9)
Preeclampsia (n, %)	10 (7.8)	5 (3.7)
Late miscarriage/preterm delivery (n, %)	4 (3.1)	16 (11.8)

Data presented as number (%) of participants.

For peer review only

Supplementary Table 3. Correlations between PROK1 level at pregnancy week 19 and BMI, HOMA-IR, fasting insulin, testosterone and androstenedione levels at inclusion and at pregnancy week 19.

	r-value	p-value	r-value	p-value
	metformin group		placebo group	
BMI at inclusion	-0.091	0.308	-0.013	0.881
BMI at week 19	-0.112	0.207	0.022	0.800
Fasting insulin at inclusion	0.097	0.312	0.017	0.856
Fasting insulin at week 19	0.075	0.432	-0.044	0.636
HOMA-IR at inclusion	0.090	0.345	0.023	0.809
HOMA-IR at week 19	0.085	0.377	-0.065	0.494
Testosterone at inclusion	0.050	0.602	0.119	0.205
Testosterone at week 19	0.123	0.203	-0.057	0.544
Androstenedione at inclusion	0.018	0.850	0.089	0.346
Androstenedione at week 19	0.087	0.308	-0.079	0.881

Abbreviations: BMI = body mass index, HOMA-IR = homeostatic model assessment of insulin resistance

For peer review only

BMJ Open

Maternal serum levels of prokineticin-1 related to pregnancy complications and metformin use in women with polycystic ovary syndrome: a post-hoc analysis of two prospective, randomized, placebo-controlled trials

Journal:	*BMJ Open*
Manuscript ID	bmjopen-2023-073619.R1
Article Type:	Original research
Date Submitted by the Author:	31-Aug-2023
Complete List of Authors:	Ujvari, Dorina; Karolinska Institute, Department of Women's and Children's Health; Karolinska Institute, Department of Microbiology, Tumor and Cell Biology; National Pandemic Centre; Centre for Translational Microbiome Research<br>Trouva, Anastasia; Karolinska Institute, Department of Molecular Medicine and Surgery; Södersjukhuset, Department of Internal Medicine; Section of Diabetes and Endocrinology<br>Linden , Angelica; Karolinska Institute; Karolinska University Hospital, Department of Gynecology and Reproductive Medicine<br>Vanky, Eszter; Norwegian University of Science and Technology, Department of Clinical and Molecular Medicine; St Olav's University Hospital, Department of Gynaecology and Obstetrics
**Primary Subject Heading**:	Obstetrics and gynaecology
Secondary Subject Heading:	Reproductive medicine
Keywords:	Maternal medicine < OBSTETRICS, Hypertension < CARDIOLOGY, Reproductive medicine < GYNAECOLOGY, Subfertility < GYNAECOLOGY

SCHOLARONE™
Manuscripts

I, the Submitting Author has the right to grant and does grant on behalf of all authors of the Work (as defined in the below author licence), an exclusive licence and/or a non-exclusive licence for contributions from authors who are: i) UK Crown employees; ii) where BMJ has agreed a CC-BY licence shall apply, and/or iii) in accordance with the terms applicable for US Federal Government officers or employees acting as part of their official duties; on a worldwide, perpetual, irrevocable, royalty-free basis to BMJ Publishing Group Ltd ("BMJ") its licensees and where the relevant Journal is co-owned by BMJ to the co-owners of the Journal, to publish the Work in this journal and any other BMJ products and to exploit all rights, as set out in our licence.

The Submitting Author accepts and understands that any supply made under these terms is made by BMJ to the Submitting Author unless you are acting as an employee on behalf of your employer or a postgraduate student of an affiliated institution which is paying any applicable article publishing charge ("APC") for Open Access articles. Where the Submitting Author wishes to make the Work available on an Open Access basis (and intends to pay the relevant APC), the terms of reuse of such Open Access shall be governed by a Creative Commons licence – details of these licences and which Creative Commons licence will apply to this Work are set out in our licence referred to above.

Other than as permitted in any relevant BMJ Author's Self Archiving Policies, I confirm this Work has not been accepted for publication elsewhere, is not being considered for publication elsewhere and does not duplicate material already published. I confirm all authors consent to publication of this Work and authorise the granting of this licence.

For peer review only

Maternal serum levels of prokineticin-1 related to pregnancy complications and metformin use in women with polycystic ovary syndrome: a post-hoc analysis of two prospective, randomized, placebo-controlled trials

Dorina Ujvari[1,2], Anastasia Trouva[3,4], Angelica Lindén Hirschberg[1,5#], Eszter Vanky[6,7#]

[1]Department of Women's and Children's Health, Karolinska Institute, Solna, Sweden
[2]Department of Microbiology, Tumor and Cell Biology, National Pandemic Centre, Centre for Translational Microbiome Research, Karolinska Institute, Solna, Sweden
[3]Department of Molecular Medicine and Surgery, Karolinska Institute, Stockholm, Sweden
[4]Department of Internal Medicine, Section of Diabetes and Endocrinology, Södersjukhuset, Stockholm, Sweden
[5]Department of Gynecology and Reproductive Medicine, Karolinska University Hospital, Solna, Sweden
[6]Department of Clinical and Molecular Medicine, Norwegian University of Science and Technology, Trondheim, Norway
[7]Department of Gynaecology and Obstetrics, St Olav's University Hospital, Trondheim, Norway

[#] These authors contributed equally to the work.

Corresponding author:
Dorina Ujvari, PhD
Department of Women's and Children's Health,
Karolinska Institutet,
SE-171 64 Stockholm, Sweden
E-mail: dorina.ujvari@ki.se

For peer review only

Abstract

Objective

Serum prokineticin-1 (s-PROK1) in the second and third trimester of pregnancy is positively correlated to preeclampsia, intrauterine growth restriction and preterm delivery. Women with polycystic ovary syndrome (PCOS) are prone to these adverse pregnancy outcomes. However, the contribution of PROK1 to the development of pregnancy complications and the effect of metformin and hyperandrogenism on s-PROK1 in PCOS have not been studied previously.

Design

This work is a post-hoc analysis of two prospective, randomized, placebo-controlled trials.

Setting

Pregnant women with PCOS were included from 11 study centres in Norway.

Participants

From 313 women, 264 participated in the present study after exclusions due to dropouts or insufficient serum samples.

Intervention

Women with PCOS were randomly assigned to metformin or placebo, from first trimester to delivery.

Primary and secondary outcome measures

S-PROK1 were analyzed using enzyme-linked immunosorbent assay at gestational week 19 and related to pregnancy complications, fasting insulin levels, HOMA-IR, testosterone, or androstenedione levels, metformin use, PCOS phenotype and hyperandrogenism.

Results

Maternal s-PROK1 in the second trimester did not predict pregnancy-induced hypertension, preeclampsia or late miscarriage/preterm delivery in women with PCOS. However, s-PROK1 was lower in women who used metformin before inclusion, both in those randomized to metformin and to placebo, compared to those who did not. S-PROK1 was also lower in those who used metformin from inclusion, or in those who used metformin both at conception and during pregnancy compared to those who did not use metformin at all. S-PROK1 was lower in those women fulfilling all three criteria

For peer review only

compared to those without hyperandrogenism and in hyperandrogenic compared to normo-androgenic women with PCOS.

Conclusions

Maternal s-PROK1 in the second trimester did not predict pregnancy complications in PCOS. Those who used metformin at conception and/or during pregnancy had lower s-PROK1. PCOS women with hyperandrogenism exhibited lower s-PROK1 compared with normo-adrogenic phenotypes.

Trial registration

The clinical trials were registered at www.clinicaltrials.gov as NCT03259919 and NCT00159536.

Strengths and limitations of this study

- This study is a post-hoc analysis of 2 randomized, double-blinded, placebo controlled, multi-center trials.
- The study includes 264 well-characterized pregnant women with PCOS.
- The number of pregnancy complications was limited, potentially leading to type II errors.
- Due to the broad timeframe of sampling in the first trimester and the lack of predictive value at week 32 and 36, s-PROK1 was measured only at week 19.

Keywords: PCOS, pregnancy, prokineticin-1, metformin, preeclampsia, placenta

Abbreviations: PROK1, prokineticin-1; s-PROK1, serum level of prokineticin-1; PCOS, polycystic ovary syndrome; HA, hyperandrogenism; PCO, polycystic ovaries; OA, oligo-amenorrhea; IUGR, intrauterine growth restriction; BMI, body mass index; HOMA-IR, homeostatic model assessment for insulin resistance

Word count: 1833

For peer review only

Introduction

Polycystic ovary syndrome (PCOS) is the most common endocrine disorder in women of reproductive age. Its prevalence is 10-17% based on at least two of the three features of PCOS (oligo-amenorrhea, hyperandrogenism and polycystic ovaries) according to the Rotterdam criteria (1). Approximately 70% of these patients have insulin resistance and compensatory hyperinsulinemia (2). PCOS is closely related to subfertility and pregnancy complications, including miscarriage, gestational diabetes, preeclampsia, intrauterine growth restriction (IUGR) and preterm delivery (3,4). Hyperinsulinemia and hyperandrogenism might contribute to the development of these complications (5,6).

Prokineticin-1 (PROK1) is a pleiotropic factor secreted by endocrine glands, with a broad range of biological functions. In the female reproductive system, it is expressed in the endometrium, ovaries, adrenal glands and placenta (7,8), and seems to be a pivotal actor at all the different stages of the gestation, from follicular development to parturition. PROK1 is significantly increased in first trimester decidua, compared with non-pregnant endometrium and controls trophoblast migration and invasion into the decidua and angiogenesis in the developing placenta (9,10). The placenta is thought to be the main source of PROK1 in the maternal circulation (11,12). In uncomplicated pregnancies, serum level of PROK1 (s-PROK1) increases 5-fold in the first trimester compared to non-pregnant state (13), and promotes the proliferation of anchoring cytotrophoblasts and formation of trophoblastic plugs in the maternal spiral arteries (11,14). After gestational week 10-12, PROK1 levels decline, facilitating the transformation of trophoblasts into an invasive phenotype, the remodeling of the maternal spiral arteries and the increase of placental oxygen tension to promote placental angiogenesis in order to supply the growing fetus (11,13). Excess expression of PROK1 was demonstrated to cause poor invasion of extravillous trophoblasts in the first trimester. This potentially leads to imperfect trophoblastic plugs resulting in early pregnancy loss, or in less severe cases to insufficient remodeling of maternal spiral arteries, and consequently to impaired placental development. The relatively hypoxic milieu in the abnormal placenta leads to the up-regulation of several hypoxia-regulated factors, among others PROK1 (10). Dysregulation of PROK1 is associated with preeclampsia, IUGR and preterm delivery (13,14,15,16). We have previously shown

For peer review only - http://bmjopen.bmj.com/site/about/guidelines.xhtml

that insulin up-regulates PROK1 in decidualizing endometrial stromal cells *in vitro*, and androgens potentiate this enhancement (17,18). Despite hyperinsulinemia/hyperandrogenism and an increased occurrence of these pregnancy complications, the role of PROK1 has not been studied in PCOS.

The administration of metformin, an insulin sensitizer, in the treatment of non-pregnant PCOS women is well established (19). Evidence shows its usefulness in reducing late miscarriages and preterm deliveries, however, its efficacy on other complications is debated and the mechanism of action is not fully understood (20,21,22,23).

The primary objective of this study was to investigate if s-PROK1 predicts pregnancy-induced hypertension, preeclampsia, and late miscarriage/preterm delivery. We hypothesized that metformin treatment was associated with lower, and hyperandrogenism with higher s-PROK1 in the second trimester.

Materials and methods

Study design

This work is a post-hoc analysis of two prospective, randomized, placebo-controlled trials, investigating the effect of metformin on pregnancy complications in women with PCOS. 264 women participated with analyzed serum samples in the present study, 128 and 136 in the metformin and placebo group, respectively (Figure 1). The clinical studies (20,21) are described in the Supplementary material.

Enzyme-linked immunosorbent assay

Serum samples, collected at gestational week 19, were analyzed by a PROK1 ELISA kit (Thermo Fischer Scientific), in 1:4 dilution. Absorbance of the samples was measured in duplicates at 450 and 550 nm on a Multiscan FC photometer, and A_{450}-A_{550} values were used to correct for optical imperfections in the microplate. To plot the standard curves, the four-parameter logistic curve fit was applied. The limit of detection (LOD) was 13.72 pg/ml. Values below the detection limit were replaced by 0.5xLOD. The intra-assay and inter-assay coefficients of variations were <10% and <12%, respectively.

For peer review only - http://bmjopen.bmj.com/site/about/guidelines.xhtml

Patient and public involvement statement

None.

Statistical analysis

Statistical procedures were performed using SPSS by IBM Version 25. Differences in baseline characteristics were analyzed using unpaired T-test and Fischer's exact test. S-PROK1 was positively skewed and therefore log-transformed. Logistic regression analysis was used to analyze whether s-PROK1 predicts pregnancy complications. Comparisons of s-PROK1 between subgroups were analyzed by Mann-Whitney U-test. Correlations between s-PROK1 and other variables were evaluated using Spearman`s rank order correlation test. $P<0.05$ was considered as statistically significant.

Results

Study population

Baseline characteristics of the women in the metformin and the placebo groups were comparable (Supplementary Table 1). Baseline characteristics were compared in additional subgroups (Supplementary Table 1). Weight, body mass index (BMI), fasting insulin levels and homeostatic model assessment for insulin resistance (HOMA-IR) were higher in hyperandrogenic compared to normo-androgenic participants (p=0.034, p=0.015, p=0.029 and p=0.034, respectively). The mode of conception was significantly different between subgroups used and not used metformin during conception (p=0.003) and between women who took metformin both at conception and during pregnancy compared to those who did not use metformin at all (p=0.048).

Pregnancy complications

Pregnancy complications are listed in Supplementary Table 2. S-PROK1 did not predict pregnancy-induced hypertension (n=15) (OR 0.95, 95% CI 0.32–2.74, p=0.92), preeclampsia (n=15) (OR 0.47, 95% CI 0.14–1.35, p=0.17) or late miscarriage/preterm delivery (n=20) (OR 0.62, 95% CI 0.24–1.56, p=0.32).

For peer review only - http://bmjopen.bmj.com/site/about/guidelines.xhtml

PROK1 and metformin use at conception, during pregnancy, and both at conception and during pregnancy

S-PROK1 was lower in women who used metformin at conception compared to those who did not, independent of later randomization to metformin or placebo (p=0.043) (Figure 2a). S-PROK1 was not different in women randomized to metformin or placebo during pregnancy (Figure 2b). Furthermore, s-PROK1 was lower in women who used metformin both at conception and during pregnancy, compared to those who used metformin during pregnancy (p=0.029) (Figure 2c). S-PROK1 was lower in women who took metformin both at conception and during pregnancy compared to those who did not use metformin at all (p=0.009) (Figure 2d).

PCOS phenotypes

Differences in s- PROK1 were also analyzed in subgroups stratified according to PCOS phenotypes. Patients from the Pilot study (n=37) were not included in this analysis, as they were diagnosed according to the NIH criteria, and due to the lack of data on the presence or absence of polycystic ovaries, those fulfilling all three criteria and hyperandrogenism-oligo-amenorrhea groups could not been distinguished. In PregMet1 samples, serum levels of PROK1 were lower in PCOS women with phenotype A (HA+PCO+OA) compared to normo-androgenic phenotype D (PCO+OA) (p=0.043) (Figure 3), while no pairwise differences were found between other PCOS phenotypes (data not shown).

S-PROK1 was lower in hyperandrogenic compared to normo-androgenic women (p=0.034) (Figure 3).

Correlations

S-PROK1 showed no correlation with BMI, fasting insulin levels, HOMA-IR, testosterone, or androstenedione levels either at inclusion or at week 19 of gestation (Supplementary Table 3).

Discussion

The primary aim of this study was to investigate if s-PROK1 predicts pregnancy-induced hypertension, preeclampsia, and late miscarriage/preterm delivery in women

For peer review only - http://bmjopen.bmj.com/site/about/guidelines.xhtml

with PCOS. Previously, s-PROK1 was reported to be higher in the second and third trimesters in preeclamptic and IUGR pregnancies compared to uncomplicated pregnancies (13,14). We and others have also demonstrated that PROK1 restricts the migration and invasion of trophoblast cells *in vitro*, suggesting that elevated s-PROK1 impairs embryo implantation and placentation (13,18). However, s-PROK1 in the second trimester did not predict pregnancy-induced hypertension or preeclampsia in our women with PCOS. It has also been demonstrated that PROK1 is up-regulated in the myometrium and the placenta during labor, suggesting a role for PROK1 in the induction of term and preterm labor (15,16). However, in this study, s-PROK1 did not predict late miscarriages/preterm deliveries in pregnant women with PCOS, but we cannot exclude that a limited number of cases might have influenced our results.

We also investigated the impact of metformin on s-PROK1 and found lower levels by metformin use at conception, both with and without metformin use after inclusion, suggesting that metformin intake, especially in early pregnancy, might have a lowering effect on s-PROK1. However, participants were not randomized at this timepoint and the lower s-PROK1 at week 19 might reflect that those who used metformin at conception differed from those who did not. It is known that metformin decreases insulin levels, although the treatment does not seem to prevent gestational diabetes in PCOS women (20,21,23). We recently demonstrated that insulin upregulates PROK1 in decidualizing endometrial stromal cells *in vitro* (18). However, in this study, we found no correlation between s-PROK1 and insulin levels.

A possible association between s-PROK1 and hyperandrogenism was also investigated. In contrast to our hypothesis, s-PROK1 was lower in women with hyperandrogenic phenotypes compared to normo-androgenic phenotype. However, there was no correlation between s-PROK1 and androgen levels at inclusion and week 19. Direct and indirect evidence support the role of androgens in the pathogenesis of placenta-related pregnancy complications (24). We have previously showed that androgens potentiate insulin to up-regulate PROK1 in decidualizing endometrial stromal cells *in vitro* (17). Increased maternal serum androgens have been reported in preeclamptic pregnancies (25,26,27,28). PCOS women with pregnancy complications have higher androgen levels than those without (29). It was also shown that trophoblast invasion and placentation are impaired in PCOS compared to controls, and this impairment is

For peer review only - http://bmjopen.bmj.com/site/about/guidelines.xhtml

associated with insulin resistance and circulating testosterone concentrations (30). However, androgens do not seem to directly contribute to the regulation of s-PROK1, although hyperandrogenism could be indirectly involved.

Strengths and limitations of this study

There are several strengths of the present study including the well-characterized women with PCOS and the randomized, double-blinded placebo-controlled multi-center study design, prospective data sampling and meticulous quality-checked diagnose control. Some limitations should also be mentioned. There was a slight difference between the two trials regarding the metformin dose, 1700 mg and 2000 mg daily, respectively, which might have affected our results. Since maternal s-PROK1 has a dynamic course during pregnancy, timing of blood sampling for determination of s-PROK1 is crucial. Based on previous studies showing elevated s-PROK1 in the second and third trimester of preeclamptic and IUGR pregnancies, we chose to analyze s-PROK1 in gestational week 19, representing the second trimester. We did not measure the s-PROK1 in serum samples taken at inclusion (between gestational week 5 and 12), as s-PROK1 has been shown to decline remarkably within this period and patients could not have been comparable. S-PROK1 was not measured at gestational week 32 and 36 either, as these late timepoints would not hold predictive values for gestational diabetes, early onset preeclampsia or late miscarriage. However, we cannot exclude that s-PROK1 should have been determined at several timepoints to study the course of PROK1 for pregnancy complications in PCOS. Furthermore, the number of pregnancy complication cases was limited possibly leading to type II errors.

For peer review only

Conclusions

S-PROK1, in the second trimester, did not predict pregnancy-induced hypertension, preeclampsia, or late miscarriage/preterm delivery in pregnant women with PCOS. Women who used metformin at conception and/or during pregnancy, those fulfilling all three criteria of PCOS in phenotype A compared to normo-androgenic phenotype D and those with hyperandrogenism compared to normo-androgenism exhibited lower s-PROK1. Further studies are needed to determine the exact role of s-PROK1 in

For peer review only - http://bmjopen.bmj.com/site/about/guidelines.xhtml

pregnancy complications and the involvement of metformin and hyperandrogenism in the regulation of PROK1 in PCOS.

Acknowledgements

The authors thank Weifa A/S for delivering metformin and placebo tablets free of charge.

Author contributions

EV conceived and designed the original protocol. On the initiative of DU and ALH, the protocol of the present study was written together with EV. DU run the analysis, DU and AT managed the database and analyzed the data with the input from EV and ALH. A statistician performed the statistical analyses. DU and AT drafted the manuscript with input from ALH, EV. All the authors contributed to the article, reviewed and approved the submitted version.

Conflicts of interest

The authors declare no conflicts of interest.

Funding information

This work was supported by grants from the Liaison Committee between the Central Norway Regional Health Authority and the Norwegian University of Science and Technology, Swedish Research Council (2021-01348), Stockholm County Council (2019-0248) and Åke Wiberg foundation (M18-0114).

Data availability

Data are available upon reasonable request.

For peer review only - http://bmjopen.bmj.com/site/about/guidelines.xhtml

Ethics statement

An informed consent was obtained from each patient and the declaration of Helsinki was followed throughout the studies. The Committee for Medical Research Ethics of Health Region IV, Norway and The Norwegian Medicines Agency approved the studies (project numbers: 51–2000 and 145.04) and the supplement application for the current study (reference number 2010/3115 (145-04)). The clinical trials were registered at www.clinicaltrials.gov as NCT03259919 (The pilot study) and NCT00159536 (The PregMet1 study).

References

1. Bozdag G, Mumusoglu S, Zengin D, et al. The prevalence and phenotypic features of polycystic ovary syndrome: a systematic review and meta-analysis. *Hum Reprod* 2016;31(12):2841-55. doi: 10.1093/humrep/dew218 [published Online First: 20160922]
2. March WA, Moore VM, Willson KJ, et al. The prevalence of polycystic ovary syndrome in a community sample assessed under contrasting diagnostic criteria. *Hum Reprod* 2010;25(2):544-51. doi: 10.1093/humrep/dep399 [published Online First: 20091112]
3. Hirschberg AL. Polycystic ovary syndrome, obesity and reproductive implications. *Womens Health (Lond)* 2009;5(5):529-40; quiz 41-2. doi: 10.2217/whe.09.39 [published Online First: 2009/08/26]
4. Franks S. Polycystic ovary syndrome. *N Engl J Med* 1995;333(13):853-61. doi: 10.1056/NEJM199509283331307
5. Azziz R, Carmina E, Chen Z, et al. Polycystic ovary syndrome. *Nat Rev Dis Primers* 2016;2:16057. doi: 10.1038/nrdp.2016.57 [published Online First: 20160811]
6. Dumesic DA, Oberfield SE, Stener-Victorin E, et al. Scientific Statement on the Diagnostic Criteria, Epidemiology, Pathophysiology, and Molecular Genetics of Polycystic Ovary Syndrome. *Endocr Rev* 2015;36(5):487-525. doi: 10.1210/er.2015-1018
7. Battersby S, Critchley HO, Morgan K, et al. Expression and regulation of the prokineticins (endocrine gland-derived vascular endothelial growth factor and Bv8) and their receptors in the human endometrium across the menstrual cycle. *The Journal of clinical endocrinology and metabolism* 2004;89(5):2463-9. doi: 10.1210/jc.2003-032012
8. Traboulsi W, Brouillet S, Sergent F, et al. Prokineticins in central and peripheral control of human reproduction. *Horm Mol Biol Clin Investig* 2015;24(2):73-81. doi: 10.1515/hmbci-2015-0040

For peer review only

9. Alfaidy N, Brouillet S, Rajaraman G, et al. The Emerging Role of the Prokineticins and Homeobox Genes in the Vascularization of the Placenta: Physiological and Pathological Aspects. *Front Physiol* 2020;11:591850. doi: 10.3389/fphys.2020.591850 [published Online First: 20201112]
10. Alfaidy N, Hoffmann P, Boufettal H, et al. The multiple roles of EG-VEGF/PROK1 in normal and pathological placental angiogenesis. *Biomed Res Int* 2014;2014:451906. doi: 10.1155/2014/451906 [published Online First: 20140515]
11. Hoffmann P, Feige JJ, Alfaidy N. Expression and oxygen regulation of endocrine gland-derived vascular endothelial growth factor/prokineticin-1 and its receptors in human placenta during early pregnancy. *Endocrinology* 2006;147(4):1675-84. doi: 10.1210/en.2005-0912
12. Brouillet S, Hoffmann P, Benharouga M, et al. Molecular characterization of EG-VEGF-mediated angiogenesis: differential effects on microvascular and macrovascular endothelial cells. *Molecular biology of the cell* 2010;21(16):2832-43. doi: 10.1091/mbc.E10-01-0059
13. Hoffmann P, Saoudi Y, Benharouga M, et al. Role of EG-VEGF in human placentation: Physiological and pathological implications. *Journal of cellular and molecular medicine* 2009;13(8B):2224-35. doi: 10.1111/j.1582-4934.2008.00554.x
14. Brouillet S, Murthi P, Hoffmann P, et al. EG-VEGF controls placental growth and survival in normal and pathological pregnancies: case of fetal growth restriction (FGR). *Cellular and molecular life sciences : CMLS* 2013;70(3):511-25. doi: 10.1007/s00018-012-1141-z
15. Catalano RD, Lannagan TR, Gorowiec M, et al. Prokineticins: novel mediators of inflammatory and contractile pathways at parturition? *Mol Hum Reprod* 2010;16(5):311-9. doi: 10.1093/molehr/gaq014 [published Online First: 20100219]
16. Gorowiec MR, Catalano RD, Norman JE, et al. Prokineticin 1 induces inflammatory response in human myometrium: a potential role in initiating term and preterm parturition. *Am J Pathol* 2011;179(6):2709-19. doi: 10.1016/j.ajpath.2011.08.029 [published Online First: 20111006]
17. Ujvari D, Graells Brugalla C, Hirschberg AL. Dihydrotestosterone potentiates insulin to up-regulate prokineticin-1 in decidualizing human endometrial stromal cells. *Journal of cellular and molecular medicine* 2020;24(5):3242-45. doi: 10.1111/jcmm.14923 [published Online First: 20200128]
18. Ujvari D, Jakson I, Oldmark C, et al. Prokineticin 1 is up-regulated by insulin in decidualizing human endometrial stromal cells. *Journal of cellular and molecular medicine* 2018;22(1):163-72. doi: 10.1111/jcmm.13305 [published Online First: 2017/08/07]
19. Teede HJ, Misso ML, Costello MF, et al. Recommendations from the international evidence-based guideline for the assessment and management of polycystic ovary syndrome. *Hum Reprod* 2018;33(9):1602-18. doi: 10.1093/humrep/dey256
20. Vanky E, Salvesen KA, Heimstad R, et al. Metformin reduces pregnancy complications without affecting androgen levels in pregnant polycystic ovary syndrome women: results of a randomized study. *Hum Reprod* 2004;19(8):1734-40. doi: 10.1093/humrep/deh347

For peer review only

21. Vanky E, Stridsklev S, Heimstad R, et al. Metformin versus placebo from first trimester to delivery in polycystic ovary syndrome: a randomized, controlled multicenter study. *The Journal of clinical endocrinology and metabolism* 2010;95(12):E448-55. doi: 10.1210/jc.2010-0853 [published Online First: 2010/10/12]
22. Fornes R, Simin J, Nguyen MH, et al. Pregnancy, perinatal and childhood outcomes in women with and without polycystic ovary syndrome and metformin during pregnancy: a nationwide population-based study. *Reprod Biol Endocrinol* 2022;20(1):30. doi: 10.1186/s12958-022-00905-6 [published Online First: 20220207]
23. Lovvik TS, Carlsen SM, Salvesen O, et al. Use of metformin to treat pregnant women with polycystic ovary syndrome (PregMet2): a randomised, double-blind, placebo-controlled trial. *Lancet Diabetes Endocrinol* 2019;7(4):256-66. doi: 10.1016/S2213-8587(19)30002-6 [published Online First: 20190218]
24. Kumar S, Gordon GH, Abbott DH, et al. Androgens in maternal vascular and placental function: implications for preeclampsia pathogenesis. *Reproduction* 2018;156(5):R155-R67. doi: 10.1530/REP-18-0278 [published Online First: 20181016]
25. Acromite MT, Mantzoros CS, Leach RE, et al. Androgens in preeclampsia. *American journal of obstetrics and gynecology* 1999;180(1 Pt 1):60-3. doi: 10.1016/s0002-9378(99)70150-x [published Online First: 1999/01/23]
26. Serin IS, Kula M, Basbug M, et al. Androgen levels of preeclamptic patients in the third trimester of pregnancy and six weeks after delivery. *Acta obstetricia et gynecologica Scandinavica* 2001;80(11):1009-13.
27. Atamer Y, Erden AC, Demir B, et al. The relationship between plasma levels of leptin and androgen in healthy and preeclamptic pregnant women. *Acta obstetricia et gynecologica Scandinavica* 2004;83(5):425-30. doi: 10.1111/j.0001-6349.2004.00276.x
28. Carlsen SM, Romundstad P, Jacobsen G. Early second-trimester maternal hyperandrogenemia and subsequent preeclampsia: a prospective study. *Acta obstetricia et gynecologica Scandinavica* 2005;84(2):117-21. doi: 10.1111/j.0001-6349.2005.00493.x
29. Falbo A, Rocca M, Russo T, et al. Changes in androgens and insulin sensitivity indexes throughout pregnancy in women with polycystic ovary syndrome (PCOS): relationships with adverse outcomes. *Journal of ovarian research* 2010;3:23. doi: 10.1186/1757-2215-3-23
30. Palomba S, Russo T, Falbo A, et al. Decidual endovascular trophoblast invasion in women with polycystic ovary syndrome: an experimental case-control study. *The Journal of clinical endocrinology and metabolism* 2012;97(7):2441-9. doi: 10.1210/jc.2012-1100

For peer review only - http://bmjopen.bmj.com/site/about/guidelines.xhtml

Figure legends

Figure 1

Flowchart of the original and the secondary studies.

Figure 2

a. Serum levels of PROK1 at pregnancy week 19 in women with PCOS, who took metformin at conception (n=83) compared to those, who did not use metformin at conception (n=181). Values are presented as median, interquartile ranges (P25-P75) and non-outlier ranges (min-max). Open circles represent outliers (1.5*the box-height from the box).

b. Serum levels of PROK1 at pregnancy week 19 in women with PCOS, who took metformin (n=128) compared to those, who took placebo after randomization (n=136). Values are presented as median, interquartile ranges (P25-P75) and non-outlier ranges (min-max). Open circles represent outliers (1.5*the box-height from the box).

c. Serum levels of PROK1 at pregnancy week 19 in women with PCOS, who took metformin both at conception and after randomization (n=42) compared to those, who did not use metformin at conception, but used after randomization (n=86). Values are presented as median, interquartile ranges (P25-P75) and non-outlier ranges (min-max). Open circles represent outliers (1.5*the box-height from the box) and the stars represent extremes (3*the box-height from the box).

d. Serum levels of PROK1 at pregnancy week 19 in women with PCOS, who took metformin both at conception and after randomization (n=42) compared to those, who did not use metformin either at conception or after randomization (n=95). Values are presented as median, interquartile ranges (P25-P75) and non-outlier ranges (min-max). Open circles represent outliers (1.5*the box-height from the box) and the stars represent extremes (3*the box-height from the box).

For peer review only

Figure 3

a. Serum levels of PROK1 at pregnancy week 19 in women with PCOS phenotype A (HA+PCO+OA) (n=132) compared to PCOS phenotype D (PCO+OA) (n=64). Values are presented as median, interquartile ranges (P25-P75) and non-outlier ranges (min-max). Open circles represent outliers (1.5*the box-height from the box).

b. Serum levels of PROK1 at pregnancy week 19 in women with PCOS and hyperandrogenism (n=164) compared with those without hyperandrogenism (n=64). Values are presented as median, interquartile ranges (P25-P75) and non-outlier ranges (min-max). Open circles represent outliers (1.5*the box-height from the box).

Supplementary material

Description and details of the two prospective, randomized, placebo-controlled trials

For peer review only

Figure 1

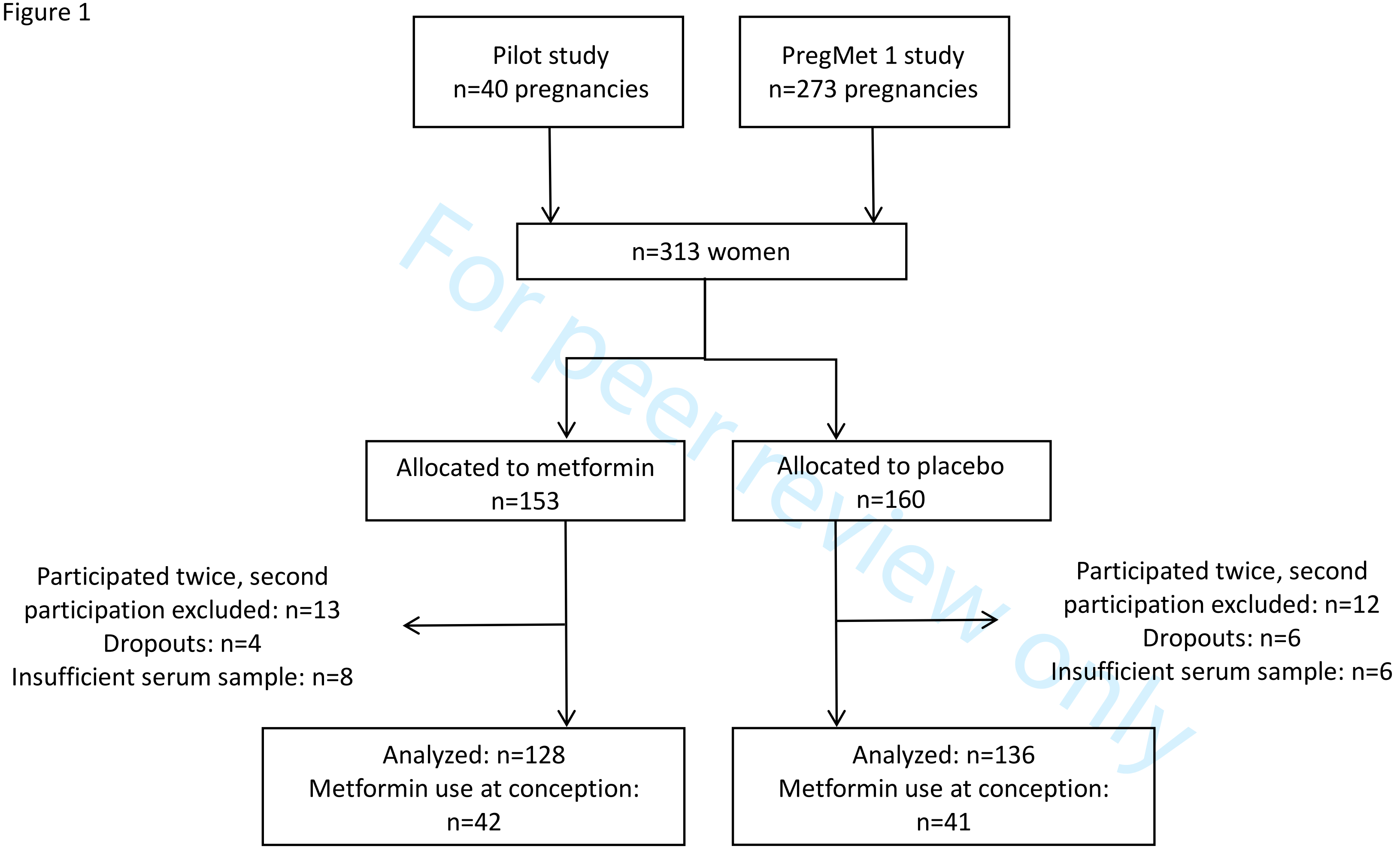

For peer review only

Figure 2

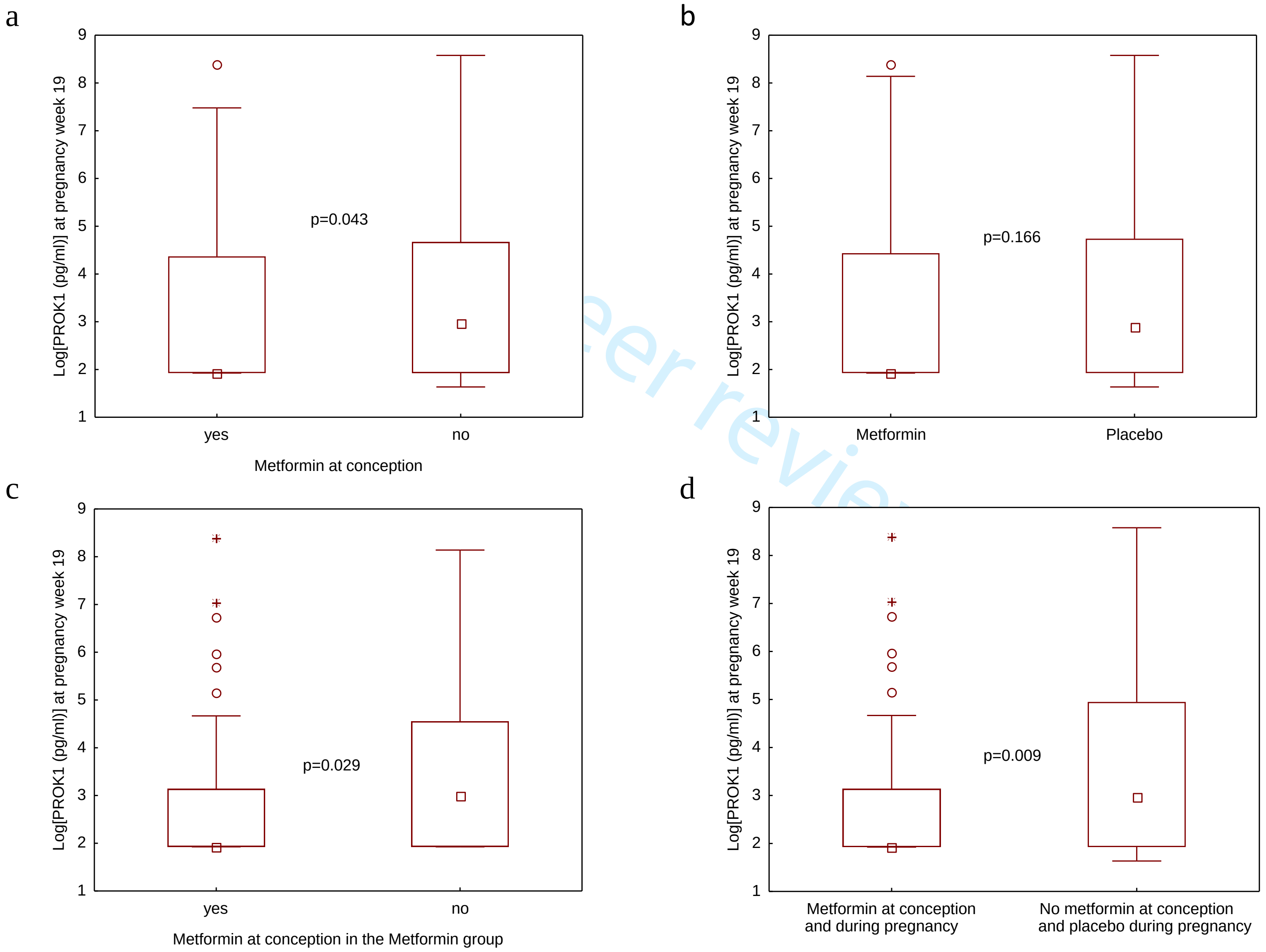
a
Log[PROK1 (pg/ml)] at pregnancy week 19
p=0.043
yes
no
Metformin at conception
b
Log[PROK1 (pg/ml)] at pregnancy week 19
p=0.166
Metformin
Placebo
c
Log[PROK1 (pg/ml)] at pregnancy week 19
p=0.029
yes
no
Metformin at conception in the Metformin group
d
Log[PROK1 (pg/ml)] at pregnancy week 19
p=0.009
Metformin at conception and during pregnancy
No metformin at conception and placebo during pregnancy

Figure 3a

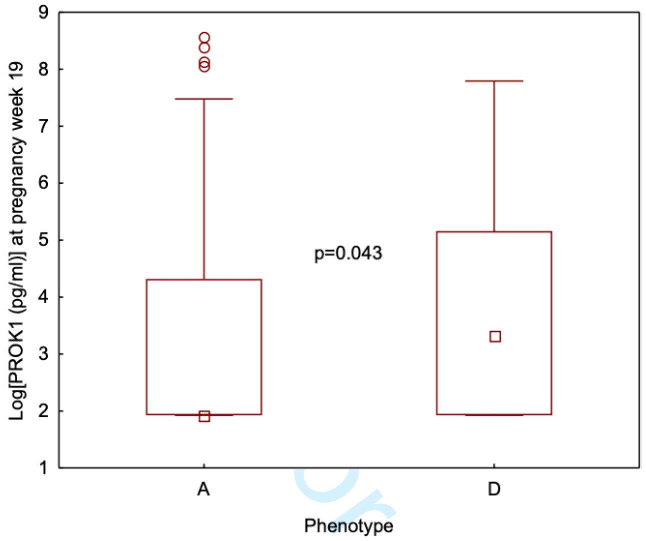

Figure 3b

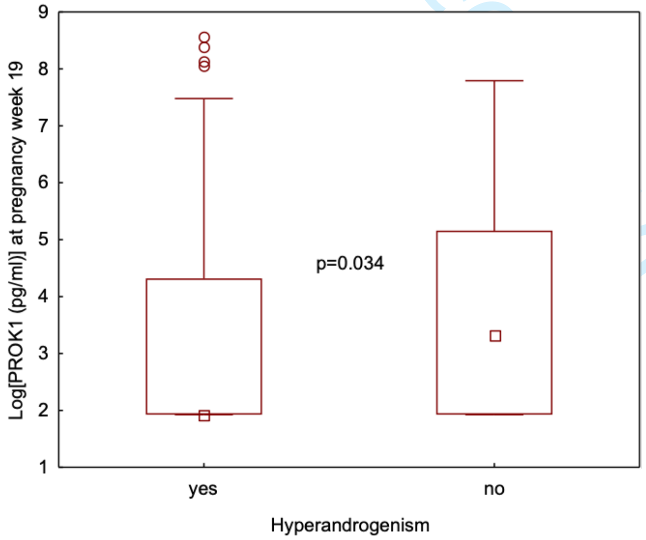

Supplementary material

All participants in the Pilot and PregMet 1 studies met the Rotterdam criteria (retrospectively evaluated in the Pilot study, as it was conducted prior to the establishment of the Rotterdam criteria), additional inclusion criteria were: age 18-45 years, gestational age between weeks 5-12, and a singleton viable fetus shown on ultrasonography. Exclusion criteria were alanine aminotransferase higher than 90 nmol/L, serum creatinine higher than 139 mmol/L, known alcohol abuse, previously diagnosed diabetes mellitus or fasting serum glucose higher than at inclusion, treatment with oral glucocorticoids, or use of drugs known to interfere with metformin. Randomization to metformin or placebo was stratified according to metformin use at conception. All participants were assigned in a double-blind manner either to metformin or placebo group. The participants received 850 mg (Pilot study) or 1000 mg (PregMet 1 study) metformin or placebo twice daily. Women who used metformin at conception had a "wash-out" period of ≥7 days before inclusion. All the participants received a 1mg tablet of folate daily, and one daily multivitamin tablet containing: vitamin A 800 mg, vitamin B_1 1.4 mg, vitamin B_2 1.6 mg, vitamin B_6 2 mg, vitamin B_{12} 1 mg, folic acid 200 mg, niacin 18 mg, pantotenic acid 6 mg, vitamin C 60 mg, vitamin D 5 mg, vitamin E 10 mg, Fe^{2+} 14 mg, Zn^{+} 15 mg, Cu^{2+} 2 mg, iodine 150 mg, Mn^{2+} 2.5 mg, Cr^{+} 50 mg, and Se^{+} 50 mg (Vitaplex^{W}; Alpharma AS, Norway).

Gestational diabetes mellitus was diagnosed as fasting plasma glucose higher than 7 mmol/L and/or 2-h serum glucose higher than 7.8 mmol/L after an OGTT (75 g glucose dissolved in 300 ml water). Pregnancy-induced hypertension was diagnosed as blood pressure of 140/90 mmHg or higher, measured after 10 minutes rest at least at two occasions. Preeclampsia was diagnosed as blood pressure of 140/90 mmHg or higher, measured on two occasions after pregnancy week 20 and albuminuria of at least 2 dipsticks on one occasion or 1 dipstick on two occasions. Late miscarriage was defined as pregnancy loss between week 13 and week 22 and 6 days, preterm delivery was defined as delivery between week 23 and week 36 and 6 days, including spontaneous deliveries, induced vaginal deliveries, and operative deliveries for medical indications.

For peer review only - http://bmjopen.bmj.com/site/about/guidelines.xhtml

Supplementary Table 1 a-e.

a) Baseline characteristics of the participants

	Metformin (n=128)	Placebo (n=136)
Age (years)	29.5 (4.5)	29.0 (4.3)
Weight (kg)	82.3 (18.9)	79.7 (18.7)
BMI (kg/m^2)	29.4 (6.6)	28.7 (7.5)
PCOS phenotype	(n=112)	(n=116)
A (PCO+OA+HA) (n, %)	65 (58.0)	67 (57.8)
B (OA+HA) (n, %)	3 (2.7)	4 (3.4)
C (PCO+HA) (n, %)	14 (12.5)	11 (9.5)
D (PCO+OA) (n, %)	30 (26.8)	34 (29.3)
hyperandrogenic (n, %)	82 (73.2)	82 (70.7)
normo-androgenic (n, %)	30 (26.8)	34 (29.3)
Mode of conception	128	136
Spontaneous (n, %)	76 (59.4)	78 (57.4)
Clomiphene citrate (n, %)	36 (28.1)	28 (20.6)
IVF/ICSI (n, %)	16 (12.5)	26 (19.1)
Other (n, %)	0 (0)	4 (2.9)
Fasting insulin at inclusion (µIU/ml)	15.7 (11.0)	15.3 (10.9)
Fasting glucose at inclusion (mmol/l)	4.6 (0.4)	4.7 (0.6)
HOMA-IR at inclusion	3.3 (2.6)	3.2 (2.3)
2-h glucose (mmol/l)	5.5 (1.5)	5.7 (1.7)
Systolic blood pressure (mm Hg)	119 (12)	117 (12)
Diastolic blood pressure (mm Hg)	75 (13)	73 (10)
Metformin at conception (n, %)	42 (32.8)	41 (30.1)
GDM at inclusion (n, %)	8 (6.3)	10 (7.4)

Abbreviations: BMI = body mass index, PCOS = polycystic ovary syndrome, PCO = polycystic ovaries, OA = oligo- and amennorhea, HA = hyperandrogenism, IVF = *in vitro* fertilization, ICSI = intracytoplasmic sperm injection, HOMA-IR = homeostatic model assessment of insulin resistance, GDM = gestational diabetes mellitus. Continuous variables are presented as mean (SD) and categorical variables as number (%) of participants.

For peer review only

b) Baseline characteristics in the whole population of participants using metformin or no metformin at conception

	Metformin at conception (n=83)	No metformin at conception (n=181)
Age (years)	29.8 (4.3)	29.0 (4.4)
Weight (kg)	82.3 (18.0)	80.3 (19.2)
BMI (kg/m^2)	29.3 (6.6)	28.9 (7.3)
PCOS phenotype	(n=77)	(n=151)
A (PCO+OA+HA) (n, %)	45 (58.4)	87 (57.6)
B (OA+HA) (n, %)	3 (3.9)	4 (2.7)
C (PCO+HA) (n, %)	7 (9.1)	18 (11.9)
D (PCO+OA) (n, %)	22 (28.6)	42 (27.8)
hyperandrogenic (n, %)	55 (71.4)	109 (72.2)
normo-androgenic (n, %)	22 (28.6)	42 (27.8)
Mode of conception $^{p=0.003}$	83	181
Spontaneous (n, %)	48 (57.8)	106 (58.6)
Clomiphene citrate (n, %)	24 (28.9)	40 (22.1)
IVF/ICSI (n, %)	7 (8.4)	35 (19.3)
Other (n, %)	4 (4.8)	0 (0)
Fasting insulin at inclusion (µIU/ml)	15.3 (8.8)	15.7 (11.8)
Fasting glucose at inclusion (mmol/l) $^{p=0.016}$	4.7 (0.6)	4.6 (0.5)
2-h glucose (mmol/l)	5.8 (1.8)	5.5 (1.5)
HOMA-IR at inclusion	3.2 (1.9)	3.2 (2.7)
Systolic blood pressure (mm Hg)	119 (13)	118 (12)
Diastolic blood pressure (mm Hg)	75 (15)	73 (9)
GDM at inclusion (n, %)	10 (13.0)	8 (5.3)

Abbreviations: BMI = body mass index, PCOS = polycystic ovary syndrome, PCO = polycystic ovaries, OA = oligo- and amennorhea, HA = hyperandrogenism, IVF = *in vitro* fertilization, ICSI = intracytoplasmic sperm injection, HOMA-IR = homeostatic model assessment of insulin resistance, GDM = gestational diabetes mellitus. Continuous variables are presented as mean (SD) and categorical variables as number (%) of participants.

For peer review only

c) Baseline characteristics of the participants in the metformin group using metformin or no metformin at conception

	Metformin at conception (n=42)	No metformin at conception (n=86)
Age (years)	30.4 (4.2)	29.1 (4.6)
Weight (kg)	83.3 (18.8)	81.8 (19.1)
BMI (kg/m^2)	29.5 (6.7)	29.3 (6.6)
PCOS phenotype	(n=40)	(n=72)
A (PCO+OA+HA) (n, %)	23 (57.5)	42 (58.3)
B (OA+HA) (n, %)	1 (2.5)	2 (2.8)
C (PCO+HA) (n, %)	5 (12.5)	9 (12.5)
D (PCO+OA) (n, %)	11 (27.5)	19 (26.4)
hyperandrogenic (n, %)	29 (72.5)	53 (73.6)
normo-androgenic (n, %)	11 (27.5)	19 (26.4)
Mode of conception	42	86
Spontaneous (n, %)	23 (54.8)	53 (61.6)
Clomiphene citrate (n, %)	15 (35.7)	21 (24.4)
IVF/ICSI (n, %)	4 (9.5)	12 (14.0)
Other (n, %)	0 (0)	0 (0)
Fasting insulin at inclusion (µIU/ml)	15.9 (9.6)	15.7 (11.7)
Fasting glucose at inclusion (mmol/l)	4.7 (0.5)	4.6 (0.4)
2-h glucose (mmol/l)	5.6 (1.7)	5.5 (1.5)
HOMA-IR at inclusion	3.3 (2.2)	3.2 (2.8)
Systolic blood pressure (mm Hg)	120 (13)	119 (12)
Diastolic blood pressure (mm Hg)	76 (18)	74 (9)
GDM at inclusion (n, %)	5 (12.5)	3 (4.2)

Abbreviations: BMI = body mass index, PCOS = polycystic ovary syndrome, PCO = polycystic ovaries, OA = oligo- and amennorhea, HA = hyperandrogenism, IVF = *in vitro* fertilization, ICSI = intracytoplasmic sperm injection, HOMA-IR = homeostatic model assessment of insulin resistance, GDM = gestational diabetes mellitus. Continuous variables are presented as mean (SD) and categorical variables as number (%) of participants.

For peer review only

d) Baseline characteristics of the participants using metformin both at conception and during pregnancy or no metformin at all

	Metformin at conception and metformin during pregnancy (n=42)	No metformin at conception and placebo during pregnancy (n=95)
Age (years)	30.4 (4.2)	29.0 (4.3)
Weight (kg)	83.3 (18.8)	79.0 (19.4)
BMI (kg/m^2)	29.5 (6.7)	28.6 (7.9)
PCOS phenotype	(n=40)	(n=79)
A (PCO+OA+HA) (n, %)	23 (57.5)	45 (57.0)
B (OA+HA) (n, %)	1 (2.5)	2 (2.5)
C (PCO+HA) (n, %)	5 (12.5)	9 (11.4)
D (PCO+OA) (n, %)	11 (27.5)	23 (29.1)
hyperandrogenic (n, %)	29 (72.5)	56 (70.9)
normo-androgenic (n, %)	11 (27.5)	23 (29.1)
Mode of conception $^{p=0.048}$	42	95
Spontaneous (n, %)	23 (54.8)	53 (55.8)
Clomiphene citrate (n, %)	15 (35.7)	19 (20.0)
IVF/ICSI (n, %)	4 (9.5)	23 (24.2)
Other (n, %)	0 (0)	0 (0)
Fasting insulin at inclusion (µIU/ml)	15.9 (9.6)	15.7 (12.1)
Fasting glucose at inclusion (mmol/l)	4.7 (0.6)	4.6 (0.5)
2-h glucose (mmol/l)	5.6 (1.7)	5.6 (1.6)
HOMA-IR at inclusion	3.3 (2.2)	3.2 (2.5)
Systolic blood pressure (mm Hg)	120 (13)	117 (12)
Diastolic blood pressure (mm Hg)	76 (18)	72 (9)
GDM at inclusion (n, %)	5 (12.5)	5 (6.3)

Abbreviations: BMI = body mass index, PCOS = polycystic ovary syndrome, PCO = polycystic ovaries, OA = oligo- and amennorhea, HA = hyperandrogenism, IVF = *in vitro* fertilization, ICSI = intracytoplasmic sperm injection, HOMA-IR = homeostatic model assessment of insulin resistance, GDM = gestational diabetes mellitus. Continuous variables are presented as mean (SD) and categorical variables as number (%) of participants.

For peer review only

e) Baseline characteristics of hyperandrogenic and normo-androgenic participants

	Hyperandrogenic PCOS phenotypes (n=164)	Normo-androgenic PCOS phenotypes (n=64)
Age (years)	29.5 (4.6)	28.9 (4.0)
Weight (kg) $^{p=0.034}$	81.8 (18.2)	76.1 (18.2)
BMI (kg/m^2) $^{p=0.015}$	29.5 (6.9)	27.0 (7.0)
Mode of conception	164	64
Spontaneous (n, %)	94 (57.4)	32 (50.0)
Clomiphene citrate (n, %)	43 (26.2)	20 (31.2)
IVF/ICSI (n, %)	24 (14.6)	11 (17.2)
Other (n, %)	3 (1.8)	1 (1.6)
Fasting insulin at inclusion (µIU/ml) $^{p=0.029}$	16.5 (12.1)	13.0 (6.7)
Fasting glucose at inclusion (mmol/l)	4.6 (0.5)	4.6 (0.6)
2-h glucose (mmol/l)	5.6 (1.6)	5.1 (1.3)
HOMA-IR at inclusion $^{p=0.034}$	3.4 (2.7)	2.7 (1.5)
Systolic blood pressure (mm Hg)	118 (12)	119 (11)
Diastolic blood pressure (mm Hg)	74 (13)	73 (9)
GDM at inclusion (n, %)	16 (9.8)	5 (3.1)

Abbreviations: BMI = body mass index, IVF = *in vitro* fertilization, ICSI = intracytoplasmic sperm injection, HOMA-IR = homeostatic model assessment of insulin resistance, GDM = gestational diabetes mellitus. Continuous variables are presented as mean (SD) and categorical variables as number (%) of participants.

For peer review only

Supplementary Table 2. Pregnancy complications in women with PCOS according to treatment with metformin or placebo during pregnancy

	Metformin (n=128)	Placebo (n=136)
Pregnancy-induced hypertension (n, %)	7 (5.5)	8 (5.9)
Preeclampsia (n, %)	10 (7.8)	5 (3.7)
Late miscarriage/preterm delivery (n, %)	4 (3.1)	16 (11.8)

Data presented as number (%) of participants.

For peer review only

Supplementary Table 3. Correlations between PROK1 level at pregnancy week 19 and BMI, HOMA-IR, fasting insulin, testosterone and androstenedione levels at inclusion and at pregnancy week 19.

	r-value	p-value	r-value	p-value
	metformin group		placebo group	
BMI at inclusion	-0.091	0.308	-0.013	0.881
BMI at week 19	-0.112	0.207	0.022	0.800
Fasting insulin at inclusion	0.097	0.312	0.017	0.856
Fasting insulin at week 19	0.075	0.432	-0.044	0.636
HOMA-IR at inclusion	0.090	0.345	0.023	0.809
HOMA-IR at week 19	0.085	0.377	-0.065	0.494
Testosterone at inclusion	0.050	0.602	0.119	0.205
Testosterone at week 19	0.123	0.203	-0.057	0.544
Androstenedione at inclusion	0.018	0.850	0.089	0.346
Androstenedione at week 19	0.087	0.308	-0.079	0.881

Abbreviations: BMI = body mass index, HOMA-IR = homeostatic model assessment of insulin resistance

For peer review only

BMJ Open

Maternal serum levels of prokineticin-1 related to pregnancy complications and metformin use in women with polycystic ovary syndrome: a post-hoc analysis of two prospective, randomized, placebo-controlled trials

Journal:	*BMJ Open*
Manuscript ID	bmjopen-2023-073619.R2
Article Type:	Original research
Date Submitted by the Author:	17-Oct-2023
Complete List of Authors:	Ujvari, Dorina; Karolinska Institute, Department of Women's and Children's Health; Karolinska Institute, Department of Microbiology, Tumor and Cell Biology; National Pandemic Centre; Centre for Translational Microbiome Research<br>Trouva, Anastasia; Karolinska Institute, Department of Molecular Medicine and Surgery<br>Linden , Angelica; Karolinska Institute; Karolinska University Hospital, Department of Gynecology and Reproductive Medicine<br>Vanky, Eszter; Norwegian University of Science and Technology, Department of Clinical and Molecular Medicine; St Olav's University Hospital, Department of Gynaecology and Obstetrics
**Primary Subject Heading**:	Obstetrics and gynaecology
Secondary Subject Heading:	Reproductive medicine
Keywords:	Maternal medicine < OBSTETRICS, Hypertension < CARDIOLOGY, Reproductive medicine < GYNAECOLOGY, Subfertility < GYNAECOLOGY

SCHOLARONE™
Manuscripts

I, the Submitting Author has the right to grant and does grant on behalf of all authors of the Work (as defined in the below author licence), an exclusive licence and/or a non-exclusive licence for contributions from authors who are: i) UK Crown employees; ii) where BMJ has agreed a CC-BY licence shall apply, and/or iii) in accordance with the terms applicable for US Federal Government officers or employees acting as part of their official duties; on a worldwide, perpetual, irrevocable, royalty-free basis to BMJ Publishing Group Ltd ("BMJ") its licensees and where the relevant Journal is co-owned by BMJ to the co-owners of the Journal, to publish the Work in this journal and any other BMJ products and to exploit all rights, as set out in our licence.

The Submitting Author accepts and understands that any supply made under these terms is made by BMJ to the Submitting Author unless you are acting as an employee on behalf of your employer or a postgraduate student of an affiliated institution which is paying any applicable article publishing charge ("APC") for Open Access articles. Where the Submitting Author wishes to make the Work available on an Open Access basis (and intends to pay the relevant APC), the terms of reuse of such Open Access shall be governed by a Creative Commons licence – details of these licences and which Creative Commons licence will apply to this Work are set out in our licence referred to above.

Other than as permitted in any relevant BMJ Author's Self Archiving Policies, I confirm this Work has not been accepted for publication elsewhere, is not being considered for publication elsewhere and does not duplicate material already published. I confirm all authors consent to publication of this Work and authorise the granting of this licence.

For peer review only

Maternal serum levels of prokineticin-1 related to pregnancy complications and metformin use in women with polycystic ovary syndrome: a post-hoc analysis of two prospective, randomized, placebo-controlled trials

Dorina Ujvari[1,2], Anastasia Trouva[3], Angelica Lindén Hirschberg[1,4#], Eszter Vanky[5,6#]

[1]Department of Women's and Children's Health, Karolinska Institute, Solna, Sweden

[2]Department of Microbiology, Tumor and Cell Biology, National Pandemic Centre, Centre for Translational Microbiome Research, Karolinska Institute, Solna, Sweden

[3]Department of Molecular Medicine and Surgery, Karolinska Institute, Stockholm, Sweden

[4]Department of Gynecology and Reproductive Medicine, Karolinska University Hospital, Solna, Sweden

[5]Department of Clinical and Molecular Medicine, Norwegian University of Science and Technology, Trondheim, Norway

[6]Department of Gynaecology and Obstetrics, St Olav's University Hospital, Trondheim, Norway

[#] These authors contributed equally to the work.

Corresponding author:
Dorina Ujvari, PhD
Department of Women's and Children's Health,
Karolinska Institutet,
SE-171 64 Stockholm, Sweden
E-mail: dorina.ujvari@ki.se

For peer review only

Abstract

Objective

Serum prokineticin-1 (s-PROK1) in the second and third trimester of pregnancy is positively correlated to preeclampsia, intrauterine growth restriction and preterm delivery. Women with polycystic ovary syndrome (PCOS) are prone to these adverse pregnancy outcomes. However, the contribution of PROK1 to the development of pregnancy complications and the effect of metformin and hyperandrogenism on s-PROK1 in PCOS have not been studied previously.

Design

This work is a post-hoc analysis of two prospective, randomized, placebo-controlled trials.

Setting

Pregnant women with PCOS were included from 11 study centres in Norway.

Participants

From 313 women, 264 participated in the present study after exclusions due to dropouts or insufficient serum samples.

Intervention

Women with PCOS were randomly assigned to metformin or placebo, from first trimester to delivery.

Primary and secondary outcome measures

S-PROK1 were analyzed using enzyme-linked immunosorbent assay at gestational week 19 and related to pregnancy complications, fasting insulin levels, HOMA-IR, testosterone, or androstenedione levels, metformin use, PCOS phenotype and hyperandrogenism.

Results

Maternal s-PROK1 in the second trimester did not predict pregnancy-induced hypertension, preeclampsia or late miscarriage/preterm delivery in women with PCOS. However, s-PROK1 was lower in women who used metformin before inclusion, both in those randomized to metformin and to placebo, compared to those who did not. S-PROK1 was also lower in those who used metformin from inclusion, or in those who used metformin both at conception and during pregnancy compared to those who did

For peer review only - http://bmjopen.bmj.com/site/about/guidelines.xhtml

not use metformin at all. S-PROK1 was lower in hyperandrogenic compared to normo-androgenic women with PCOS.

Conclusions

Maternal s-PROK1 in the second trimester did not predict pregnancy complications in PCOS. Those who used metformin at conception and/or during pregnancy had lower s-PROK1. PCOS women with hyperandrogenism exhibited lower s-PROK1 compared with normo-adrogenic phenotypes.

Trial registration

The clinical trials were registered at www.clinicaltrials.gov as NCT03259919 and NCT00159536.

Strengths and limitations of this study

- This study is a post-hoc analysis of 2 randomized, double-blinded, placebo controlled, multi-center trials.
- The study includes 264 well-characterized pregnant women with PCOS.
- The number of pregnancy complications was limited, potentially leading to type II errors.
- Due to the broad timeframe of sampling in the first trimester and the lack of predictive value at week 32 and 36, s-PROK1 was measured only at week 19.

Keywords: PCOS, pregnancy, prokineticin-1, metformin, preeclampsia, placenta

Abbreviations: PROK1, prokineticin-1; s-PROK1, serum level of prokineticin-1; PCOS, polycystic ovary syndrome; HA, hyperandrogenism; PCO, polycystic ovaries; OA, oligo-amenorrhea; IUGR, intrauterine growth restriction; BMI, body mass index; HOMA-IR, homeostatic model assessment for insulin resistance

Word count: 1889

For peer review only - http://bmjopen.bmj.com/site/about/guidelines.xhtml

Introduction

Polycystic ovary syndrome (PCOS) is the most common endocrine disorder in women of reproductive age. Its prevalence is 10-17% based on at least two of the three features of PCOS (oligo-amenorrhea, hyperandrogenism and polycystic ovaries) according to the Rotterdam criteria (1). Approximately 70% of these patients have insulin resistance and compensatory hyperinsulinemia (2). PCOS is closely related to subfertility and pregnancy complications, including miscarriage, gestational diabetes, preeclampsia, intrauterine growth restriction (IUGR) and preterm delivery (3,4). Hyperinsulinemia and hyperandrogenism might contribute to the development of these complications (5,6).

Prokineticin-1 (PROK1) is a pleiotropic factor secreted by endocrine glands, with a broad range of biological functions. In the female reproductive system, it is expressed in the endometrium, ovaries, adrenal glands and placenta (7,8), and seems to be a pivotal actor at all the different stages of the gestation, from follicular development to parturition. PROK1 is significantly increased in first trimester decidua, compared with non-pregnant endometrium and controls trophoblast migration and invasion into the decidua and angiogenesis in the developing placenta (9,10). The placenta is thought to be the main source of PROK1 in the maternal circulation (11,12). In uncomplicated pregnancies, serum level of PROK1 (s-PROK1) increases 5-fold in the first trimester compared to non-pregnant state (13), and promotes the proliferation of anchoring cytotrophoblasts and formation of trophoblastic plugs in the maternal spiral arteries (11,14). After gestational week 10-12, PROK1 levels decline, facilitating the transformation of trophoblasts into an invasive phenotype, the remodeling of the maternal spiral arteries and the increase of placental oxygen tension to promote placental angiogenesis in order to supply the growing fetus (11,13). Excess expression of PROK1 was demonstrated to cause poor invasion of extravillous trophoblasts in the first trimester. This potentially leads to imperfect trophoblastic plugs resulting in early pregnancy loss, or in less severe cases to insufficient remodeling of maternal spiral arteries, and consequently to impaired placental development. The relatively hypoxic milieu in the abnormal placenta leads to the up-regulation of several hypoxia-regulated factors, among others PROK1 (10). Dysregulation of PROK1 is associated with preeclampsia, IUGR and preterm delivery (13,14,15,16). We have previously shown

For peer review only - http://bmjopen.bmj.com/site/about/guidelines.xhtml

that insulin up-regulates PROK1 in decidualizing endometrial stromal cells *in vitro*, and androgens potentiate this enhancement (17,18). Despite hyperinsulinemia/hyperandrogenism and an increased occurrence of these pregnancy complications, the role of PROK1 has not been studied in PCOS.

The administration of metformin, an insulin sensitizer, in the treatment of non-pregnant PCOS women is well established (19). Evidence shows its usefulness in reducing late miscarriages and preterm deliveries, however, its efficacy on other complications is debated and the mechanism of action is not fully understood (20,21,22,23).

The primary objective of this study was to investigate if s-PROK1 predicts pregnancy-induced hypertension, preeclampsia, and late miscarriage/preterm delivery. We hypothesized that metformin treatment was associated with lower, and hyperandrogenism with higher s-PROK1 in the second trimester.

Materials and methods

Study design

This work is a post-hoc analysis of two prospective, randomized, placebo-controlled trials, investigating the effect of metformin on pregnancy complications in women with PCOS. 264 women participated with analyzed serum samples in the present study, 128 and 136 in the metformin and placebo group, respectively (Figure 1). The clinical studies (20,21) are described in the Supplementary material.

Enzyme-linked immunosorbent assay

Serum samples, collected at gestational week 19, were analyzed by a PROK1 ELISA kit (Thermo Fischer Scientific), in 1:4 dilution. Absorbance of the samples was measured in duplicates at 450 and 550 nm on a Multiscan FC photometer, and A_{450}-A_{550} values were used to correct for optical imperfections in the microplate. To plot the standard curves, the four-parameter logistic curve fit was applied. The limit of detection (LOD) was 13.72 pg/ml. Values below the detection limit were replaced by 0.5xLOD. The intra-assay and inter-assay coefficients of variations were <10% and <12%, respectively.

For peer review only - http://bmjopen.bmj.com/site/about/guidelines.xhtml

Patient and public involvement statement

None.

Statistical analysis

Statistical procedures were performed using SPSS by IBM Version 25 and Statistica 14.0 TIBCO software Inc. Differences in baseline characteristics were analyzed using unpaired T-test and Fischer's exact test. S-PROK1 was positively skewed and therefore log-transformed. Logistic regression analysis was used to analyze whether s-PROK1 predicts pregnancy complications. Differences of s-PROK1 between subgroups were analyzed by median test, followed by pairwise median test with Bonferroni correction, in case of statistical significance. Correlations between s-PROK1 and other variables were evaluated using Spearman`s rank order correlation test. P<0.05 was considered as statistically significant.

Results

Study population

Baseline characteristics of the women in the metformin and the placebo groups were comparable (Supplementary Table 1). Baseline characteristics were compared in additional subgroups (Supplementary Table 1). Weight, body mass index (BMI), fasting insulin levels and homeostatic model assessment for insulin resistance (HOMA-IR) were higher in hyperandrogenic compared to normo-androgenic participants (p=0.034, p=0.015, p=0.029 and p=0.034, respectively). The mode of conception was significantly different between subgroups used and not used metformin during conception (p=0.003) and between women who took metformin both at conception and during pregnancy compared to those who did not use metformin at all (p=0.048).

Pregnancy complications

Pregnancy complications are listed in Supplementary Table 2. S-PROK1 did not predict pregnancy-induced hypertension (n=15) (OR 0.95, 95% CI 0.32–2.74, p=0.92), preeclampsia (n=15) (OR 0.47, 95% CI 0.14–1.35, p=0.17) or late miscarriage/preterm

For peer review only - http://bmjopen.bmj.com/site/about/guidelines.xhtml

delivery (n=20) (OR 0.62, 95% CI 0.24–1.56, p=0.32).

PROK1 and metformin use at conception, during pregnancy, and both at conception and during pregnancy

S-PROK1 was lower in women who used metformin at conception (median=6.86, 95% CI 6.86-11.22) compared to those who did not (median=19.53, 95% CI 7.00-30.83), regardless of later randomization to metformin (median=6.86, 95% CI 6.86-20.51) or placebo (median=17.75, 95% CI 7.00-30.83) (p=0.012) (Figure 2a). S-PROK1 was not different in women randomized to metformin or placebo during pregnancy (Figure 2b). Median test showed a statistically significant difference between the four subgroups of no metformin use/metformin use at conception combined with metformin/placebo use during pregnancy (p=0.020). Pairwise median test revealed that s-PROK1 was lower in women who used metformin both at conception and during pregnancy (median=6.86, 95% CI 6.86-18.00), compared to those who used metformin during pregnancy (median=19.76, 95% CI 9.60-46.46) (p=0.008, $p_{adjusted}$=0.031) (Figure 3). S-PROK1 was lower in women who took metformin both at conception and during pregnancy compared to those who did not use metformin at all (median=19.53, 95% CI 7.00-40.36) (p=0.001, $p_{adjusted}$=0.004) (Figure 3).

PCOS phenotypes

Differences in s- PROK1 were also analyzed in subgroups stratified according to PCOS phenotypes. Patients from the Pilot study (n=37) were not included in this analysis, as they were diagnosed according to the NIH criteria, and due to the lack of data on the presence or absence of polycystic ovaries, those fulfilling all three criteria and hyperandrogenism-oligo-amenorrhea groups could not been distinguished. In PregMet1 samples, s-PROK1 was not significantly different between PCOS phenotypes A (HA+PCO+OA), B (HA+PCO), C (HA+OA) and D (PCO+OA) (data not shown). S-PROK1 was lower in hyperandrogenic (median=6.86, 95% CI 6.86-9.69) compared to normo-androgenic women (median=6.86, 95% CI 7.00-88.69) (p=0.040) (Figure 4).

Correlations

For peer review only - http://bmjopen.bmj.com/site/about/guidelines.xhtml

S-PROK1 showed no correlation with BMI, fasting insulin levels, HOMA-IR, testosterone, or androstenedione levels either at inclusion or at week 19 of gestation (Supplementary Table 3).

Discussion

The primary aim of this study was to investigate if s-PROK1 predicts pregnancy-induced hypertension, preeclampsia, and late miscarriage/preterm delivery in women with PCOS. Previously, s-PROK1 was reported to be higher in the second and third trimesters in preeclamptic and IUGR pregnancies compared to uncomplicated pregnancies (13,14). We and others have also demonstrated that PROK1 restricts the migration and invasion of trophoblast cells *in vitro*, suggesting that elevated s-PROK1 impairs embryo implantation and placentation (13,18). However, s-PROK1 in the second trimester did not predict pregnancy-induced hypertension or preeclampsia in our women with PCOS. It has also been demonstrated that PROK1 is up-regulated in the myometrium and the placenta during labor, suggesting a role for PROK1 in the induction of term and preterm labor (15,16). However, in this study, s-PROK1 did not predict late miscarriages/preterm deliveries in pregnant women with PCOS, but we cannot exclude that a limited number of cases might have influenced our results.

We also investigated the impact of metformin on s-PROK1 and found lower levels by metformin use at conception, both with and without metformin use after inclusion, suggesting that metformin intake, especially in early pregnancy, might have a lowering effect on s-PROK1. However, participants were not randomized at this timepoint and the lower s-PROK1 at week 19 might reflect that those who used metformin at conception differed from those who did not. It is known that metformin decreases insulin levels, although the treatment does not seem to prevent gestational diabetes in PCOS women (20,21,23). We recently demonstrated that insulin upregulates PROK1 in decidualizing endometrial stromal cells *in vitro* (18). However, in this study, we found no correlation between s-PROK1 and insulin levels.

A possible association between s-PROK1 and hyperandrogenism was also investigated. In contrast to our hypothesis, s-PROK1 was lower in women with hyperandrogenic

For peer review only

phenotypes compared to normo-androgenic phenotype. However, there was no correlation between s-PROK1 and androgen levels at inclusion and week 19. Direct and indirect evidence support the role of androgens in the pathogenesis of placenta-related pregnancy complications (24). We have previously showed that androgens potentiate insulin to up-regulate PROK1 in decidualizing endometrial stromal cells *in vitro* (17). Increased maternal serum androgens have been reported in preeclamptic pregnancies (25,26,27,28). PCOS women with pregnancy complications have higher androgen levels than those without (29). It was also shown that trophoblast invasion and placentation are impaired in PCOS compared to controls, and this impairment is associated with insulin resistance and circulating testosterone concentrations (30). However, androgens do not seem to directly contribute to the regulation of s-PROK1, although hyperandrogenism could be indirectly involved.

Strengths and limitations of this study

There are several strengths of the present study including the well-characterized women with PCOS and the randomized, double-blinded placebo-controlled multi-center study design, prospective data sampling and meticulous quality-checked diagnosis control. Some limitations should also be mentioned. There was a slight difference between the two trials regarding the metformin dose, 1700 mg and 2000 mg daily, respectively, which might have affected our results. Since maternal s-PROK1 has a dynamic course during pregnancy, timing of blood sampling for determination of s-PROK1 is crucial. Based on previous studies showing elevated s-PROK1 in the second and third trimester of preeclamptic and IUGR pregnancies, we chose to analyze s-PROK1 in gestational week 19, representing the second trimester. We did not measure the s-PROK1 in serum samples taken at inclusion (between gestational week 5 and 12), as s-PROK1 has been shown to decline remarkably within this period and patients could not have been comparable. S-PROK1 was not measured at gestational week 32 and 36 either, as these late timepoints would not hold predictive values for gestational diabetes, early onset preeclampsia or late miscarriage. However, we cannot exclude that s-PROK1 should have been determined at several timepoints to study the course of PROK1 for pregnancy complications in PCOS. Furthermore, the number of pregnancy complication cases was limited possibly leading to type II errors.

For peer review only - http://bmjopen.bmj.com/site/about/guidelines.xhtml

Conclusions

S-PROK1, in the second trimester, did not predict pregnancy-induced hypertension, preeclampsia, or late miscarriage/preterm delivery in pregnant women with PCOS. Women who used metformin at conception and/or during pregnancy and those with hyperandrogenism compared to normo-androgenism exhibited lower s-PROK1. Further studies are needed to determine the exact role of s-PROK1 in pregnancy complications and the involvement of metformin and hyperandrogenism in the regulation of PROK1 in PCOS.

Acknowledgements

The authors thank Weifa A/S for delivering metformin and placebo tablets free of charge.

Author contributions

EV conceived and designed the original protocol. On the initiative of DU and ALH, the protocol of the present study was written together with EV. DU run the analysis, DU and AT managed the database and analyzed the data with the input from EV and ALH. A statistician performed the statistical analyses. DU and AT drafted the manuscript with input from ALH, EV. All the authors contributed to the article, reviewed and approved the submitted version.

Conflicts of interest

The authors declare no conflicts of interest.

Funding information

This work was supported by grants from the Liaison Committee between the Central Norway Regional Health Authority and the Norwegian University of Science and

For peer review only

Technology, Swedish Research Council (2021-01348), Stockholm County Council (2019-0248) and Åke Wiberg foundation (M18-0114).

Data availability

Data are available upon reasonable request.

Ethics statement

An informed consent was obtained from each patient and the declaration of Helsinki was followed throughout the studies. The Committee for Medical Research Ethics of Health Region IV, Norway and The Norwegian Medicines Agency approved the studies (project numbers: 51–2000 and 145.04) and the supplement application for the current study (reference number 2010/3115 (145-04)). The clinical trials were registered at www.clinicaltrials.gov as NCT03259919 (The pilot study) and NCT00159536 (The PregMet1 study).

References

1. Bozdag G, Mumusoglu S, Zengin D, et al. The prevalence and phenotypic features of polycystic ovary syndrome: a systematic review and meta-analysis. *Hum Reprod* 2016;31(12):2841-55. doi: 10.1093/humrep/dew218 [published Online First: 20160922]
2. March WA, Moore VM, Willson KJ, et al. The prevalence of polycystic ovary syndrome in a community sample assessed under contrasting diagnostic criteria. *Hum Reprod* 2010;25(2):544-51. doi: 10.1093/humrep/dep399 [published Online First: 20091112]
3. Hirschberg AL. Polycystic ovary syndrome, obesity and reproductive implications. *Womens Health (Lond)* 2009;5(5):529-40; quiz 41-2. doi: 10.2217/whe.09.39 [published Online First: 2009/08/26]
4. Franks S. Polycystic ovary syndrome. *N Engl J Med* 1995;333(13):853-61. doi: 10.1056/NEJM199509283331307
5. Azziz R, Carmina E, Chen Z, et al. Polycystic ovary syndrome. *Nat Rev Dis Primers* 2016;2:16057. doi: 10.1038/nrdp.2016.57 [published Online First: 20160811]
6. Dumesic DA, Oberfield SE, Stener-Victorin E, et al. Scientific Statement on the Diagnostic Criteria, Epidemiology, Pathophysiology, and Molecular

For peer review only - http://bmjopen.bmj.com/site/about/guidelines.xhtml

Genetics of Polycystic Ovary Syndrome. *Endocr Rev* 2015;36(5):487-525. doi: 10.1210/er.2015-1018
7. Battersby S, Critchley HO, Morgan K, et al. Expression and regulation of the prokineticins (endocrine gland-derived vascular endothelial growth factor and Bv8) and their receptors in the human endometrium across the menstrual cycle. *The Journal of clinical endocrinology and metabolism* 2004;89(5):2463-9. doi: 10.1210/jc.2003-032012
8. Traboulsi W, Brouillet S, Sergent F, et al. Prokineticins in central and peripheral control of human reproduction. *Horm Mol Biol Clin Investig* 2015;24(2):73-81. doi: 10.1515/hmbci-2015-0040
9. Alfaidy N, Brouillet S, Rajaraman G, et al. The Emerging Role of the Prokineticins and Homeobox Genes in the Vascularization of the Placenta: Physiological and Pathological Aspects. *Front Physiol* 2020;11:591850. doi: 10.3389/fphys.2020.591850 [published Online First: 20201112]
10. Alfaidy N, Hoffmann P, Boufettal H, et al. The multiple roles of EG-VEGF/PROK1 in normal and pathological placental angiogenesis. *Biomed Res Int* 2014;2014:451906. doi: 10.1155/2014/451906 [published Online First: 20140515]
11. Hoffmann P, Feige JJ, Alfaidy N. Expression and oxygen regulation of endocrine gland-derived vascular endothelial growth factor/prokineticin-1 and its receptors in human placenta during early pregnancy. *Endocrinology* 2006;147(4):1675-84. doi: 10.1210/en.2005-0912
12. Brouillet S, Hoffmann P, Benharouga M, et al. Molecular characterization of EG-VEGF-mediated angiogenesis: differential effects on microvascular and macrovascular endothelial cells. *Molecular biology of the cell* 2010;21(16):2832-43. doi: 10.1091/mbc.E10-01-0059
13. Hoffmann P, Saoudi Y, Benharouga M, et al. Role of EG-VEGF in human placentation: Physiological and pathological implications. *Journal of cellular and molecular medicine* 2009;13(8B):2224-35. doi: 10.1111/j.1582-4934.2008.00554.x
14. Brouillet S, Murthi P, Hoffmann P, et al. EG-VEGF controls placental growth and survival in normal and pathological pregnancies: case of fetal growth restriction (FGR). *Cellular and molecular life sciences : CMLS* 2013;70(3):511-25. doi: 10.1007/s00018-012-1141-z
15. Catalano RD, Lannagan TR, Gorowiec M, et al. Prokineticins: novel mediators of inflammatory and contractile pathways at parturition? *Mol Hum Reprod* 2010;16(5):311-9. doi: 10.1093/molehr/gaq014 [published Online First: 20100219]
16. Gorowiec MR, Catalano RD, Norman JE, et al. Prokineticin 1 induces inflammatory response in human myometrium: a potential role in initiating term and preterm parturition. *Am J Pathol* 2011;179(6):2709-19. doi: 10.1016/j.ajpath.2011.08.029 [published Online First: 20111006]
17. Ujvari D, Graells Brugalla C, Hirschberg AL. Dihydrotestosterone potentiates insulin to up-regulate prokineticin-1 in decidualizing human endometrial stromal cells. *Journal of cellular and molecular medicine* 2020;24(5):3242-45. doi: 10.1111/jcmm.14923 [published Online First: 20200128]
18. Ujvari D, Jakson I, Oldmark C, et al. Prokineticin 1 is up-regulated by insulin in decidualizing human endometrial stromal cells. *Journal of cellular and*

For peer review only - http://bmjopen.bmj.com/site/about/guidelines.xhtml

molecular medicine 2018;22(1):163-72. doi: 10.1111/jcmm.13305 [published Online First: 2017/08/07]
19. Teede HJ, Misso ML, Costello MF, et al. Recommendations from the international evidence-based guideline for the assessment and management of polycystic ovary syndrome. *Hum Reprod* 2018;33(9):1602-18. doi: 10.1093/humrep/dey256
20. Vanky E, Salvesen KA, Heimstad R, et al. Metformin reduces pregnancy complications without affecting androgen levels in pregnant polycystic ovary syndrome women: results of a randomized study. *Hum Reprod* 2004;19(8):1734-40. doi: 10.1093/humrep/deh347
21. Vanky E, Stridsklev S, Heimstad R, et al. Metformin versus placebo from first trimester to delivery in polycystic ovary syndrome: a randomized, controlled multicenter study. *The Journal of clinical endocrinology and metabolism* 2010;95(12):E448-55. doi: 10.1210/jc.2010-0853 [published Online First: 2010/10/12]
22. Fornes R, Simin J, Nguyen MH, et al. Pregnancy, perinatal and childhood outcomes in women with and without polycystic ovary syndrome and metformin during pregnancy: a nationwide population-based study. *Reprod Biol Endocrinol* 2022;20(1):30. doi: 10.1186/s12958-022-00905-6 [published Online First: 20220207]
23. Lovvik TS, Carlsen SM, Salvesen O, et al. Use of metformin to treat pregnant women with polycystic ovary syndrome (PregMet2): a randomised, double-blind, placebo-controlled trial. *Lancet Diabetes Endocrinol* 2019;7(4):256-66. doi: 10.1016/S2213-8587(19)30002-6 [published Online First: 20190218]
24. Kumar S, Gordon GH, Abbott DH, et al. Androgens in maternal vascular and placental function: implications for preeclampsia pathogenesis. *Reproduction* 2018;156(5):R155-R67. doi: 10.1530/REP-18-0278 [published Online First: 20181016]
25. Acromite MT, Mantzoros CS, Leach RE, et al. Androgens in preeclampsia. *American journal of obstetrics and gynecology* 1999;180(1 Pt 1):60-3. doi: 10.1016/s0002-9378(99)70150-x [published Online First: 1999/01/23]
26. Serin IS, Kula M, Basbug M, et al. Androgen levels of preeclamptic patients in the third trimester of pregnancy and six weeks after delivery. *Acta obstetricia et gynecologica Scandinavica* 2001;80(11):1009-13.
27. Atamer Y, Erden AC, Demir B, et al. The relationship between plasma levels of leptin and androgen in healthy and preeclamptic pregnant women. *Acta obstetricia et gynecologica Scandinavica* 2004;83(5):425-30. doi: 10.1111/j.0001-6349.2004.00276.x
28. Carlsen SM, Romundstad P, Jacobsen G. Early second-trimester maternal hyperandrogenemia and subsequent preeclampsia: a prospective study. *Acta obstetricia et gynecologica Scandinavica* 2005;84(2):117-21. doi: 10.1111/j.0001-6349.2005.00493.x
29. Falbo A, Rocca M, Russo T, et al. Changes in androgens and insulin sensitivity indexes throughout pregnancy in women with polycystic ovary syndrome (PCOS): relationships with adverse outcomes. *Journal of ovarian research* 2010;3:23. doi: 10.1186/1757-2215-3-23
30. Palomba S, Russo T, Falbo A, et al. Decidual endovascular trophoblast invasion in women with polycystic ovary syndrome: an experimental

For peer review only

case-control study. *The Journal of clinical endocrinology and metabolism* 2012;97(7):2441-9. doi: 10.1210/jc.2012-1100

For peer review only

Figure legends

Figure 1

Flowchart of the original and the secondary studies.

Figure 2

a. Serum levels of PROK1 at pregnancy week 19 in women with PCOS, who took metformin at conception (n=83) compared to those, who did not use metformin at conception (n=181). Values are presented as median, interquartile ranges (P25-P75) and non-outlier ranges (min-max). Open circles represent outliers (1.5*the box-height from the box).

b. Serum levels of PROK1 at pregnancy week 19 in women with PCOS, who took metformin (n=128) compared to those, who took placebo after randomization (n=136). Values are presented as median, interquartile ranges (P25-P75) and non-outlier ranges (min-max). Open circles represent outliers (1.5*the box-height from the box).

Figure 3

Serum levels of PROK1 at pregnancy week 19 in women with PCOS, who did not use metformin/used metformin at conception in the placebo group (n=41 and n=95, respectively) and in the metformin group (n=42 and n=86, respectively). Values are presented as median, interquartile ranges (P25-P75) and non-outlier ranges (min-max). Open circles represent outliers (1.5*the box-height from the box) and the stars represent extremes (3*the box-height from the box).

Figure 4

Serum levels of PROK1 at pregnancy week 19 in women with PCOS and hyperandrogenism (n=164) compared with those without hyperandrogenism (n=64). Values are presented as median, interquartile ranges (P25-P75) and non-outlier ranges (min-max). Open circles represent outliers (1.5*the box-height from the box).

Supplementary material

Description and details of the two prospective, randomized, placebo-controlled trials.

For peer review only - http://bmjopen.bmj.com/site/about/guidelines.xhtml

Figure 1

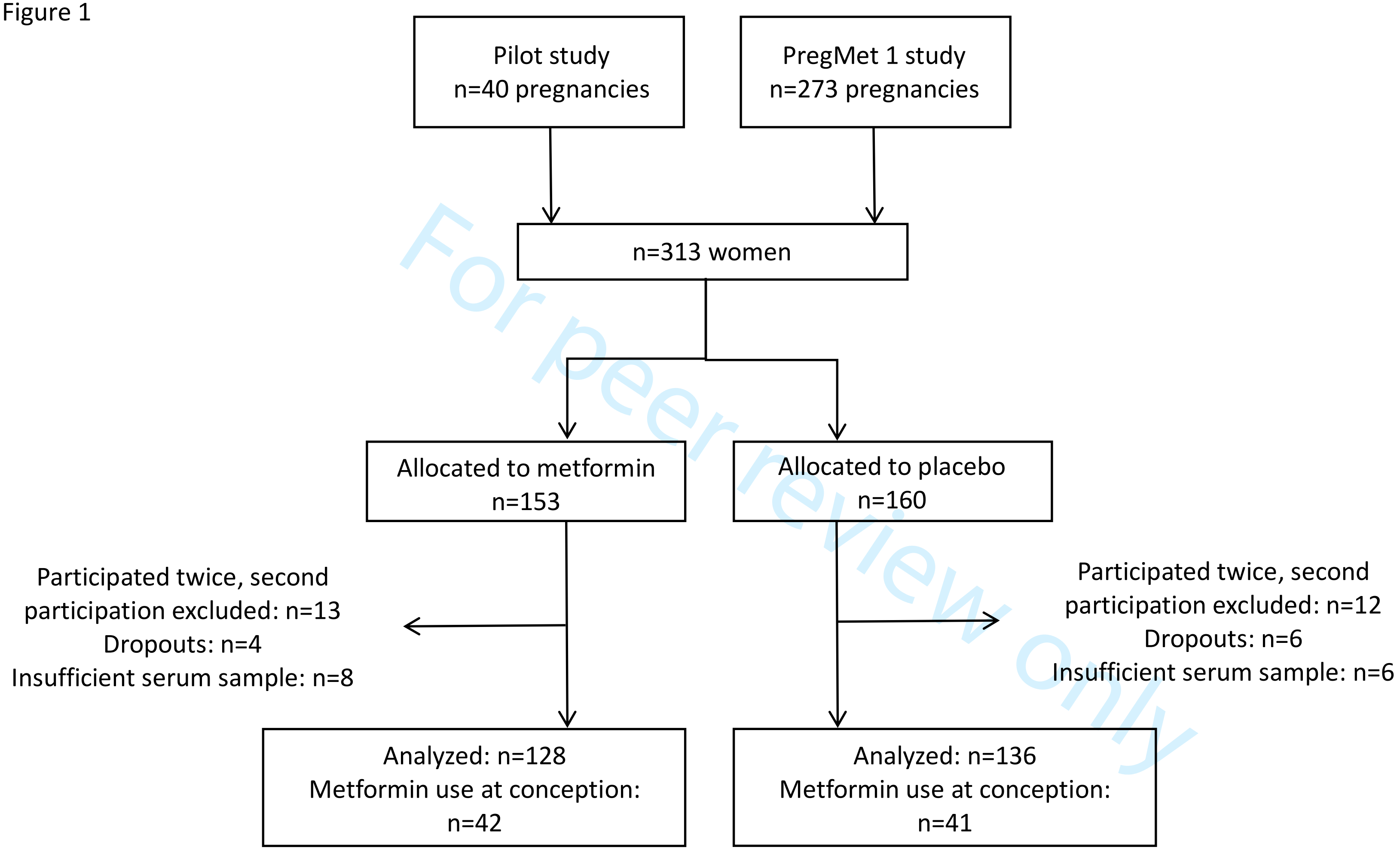

For peer review only

Figure 2

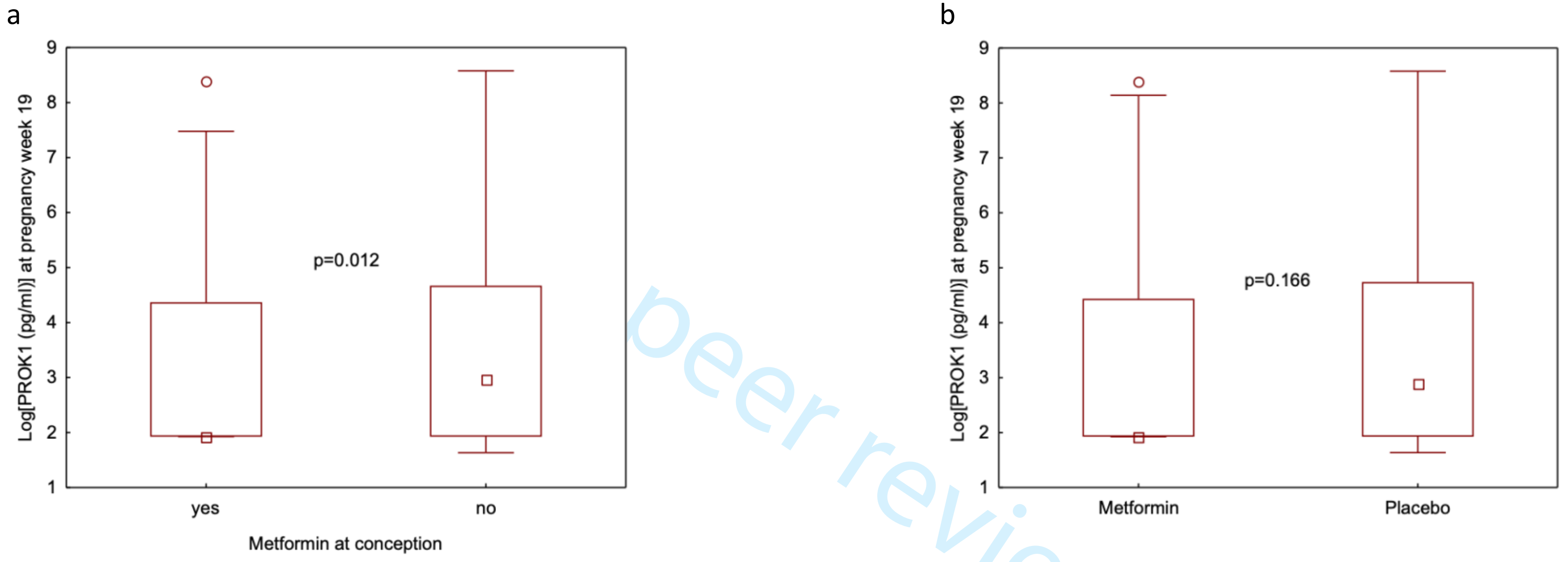

For peer review only - http://bmjopen.bmj.com/site/about/guidelines.xhtml

Figure 3

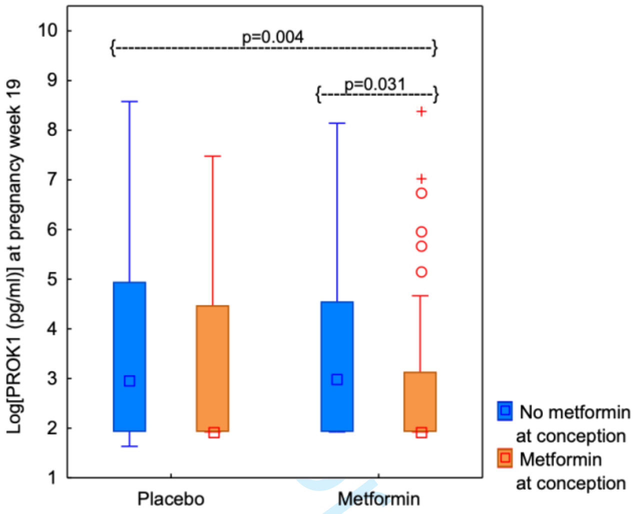

For peer review only

Figure 4

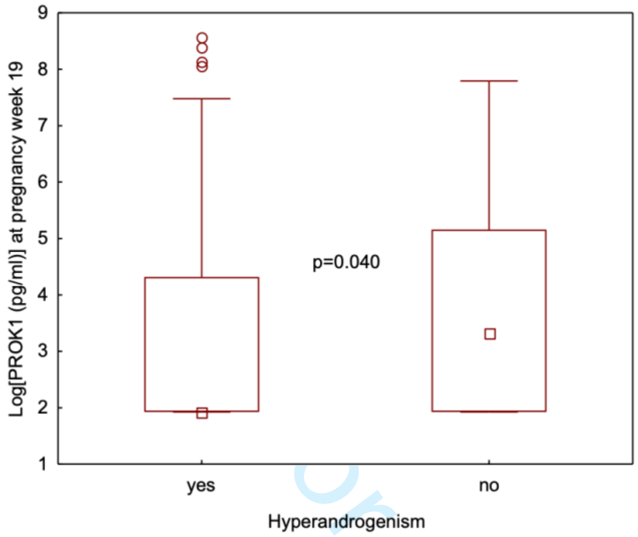
Log[PROK1 (pg/ml)] at pregnancy week 19
9
8
7
6
5
4
3
2
1
p=0.040
yes
no
Hyperandrogenism

Supplementary Table 1 a-e.

a) Baseline characteristics of the participants

	Metformin (n=128)	Placebo (n=136)
Age (years)	29.5 (4.5)	29.0 (4.3)
Weight (kg)	82.3 (18.9)	79.7 (18.7)
BMI (kg/m^2)	29.4 (6.6)	28.7 (7.5)
PCOS phenotype	n=112	n=116
A (PCO+OA+HA) (n, %)	65 (58.0)	67 (57.8)
B (OA+HA) (n, %)	3 (2.7)	4 (3.4)
C (PCO+HA) (n, %)	14 (12.5)	11 (9.5)
D (PCO+OA) (n, %)	30 (26.8)	34 (29.3)
hyperandrogenic (n, %)	82 (73.2)	82 (70.7)
normo-androgenic (n, %)	30 (26.8)	34 (29.3)
Mode of conception	n=128	n=136
Spontaneous (n, %)	76 (59.4)	78 (57.4)
Clomiphene citrate (n, %)	36 (28.1)	28 (20.6)
IVF/ICSI (n, %)	16 (12.5)	26 (19.1)
Other (n, %)	0 (0)	4 (2.9)
Fasting insulin at inclusion (µIU/ml)	15.7 (11.0)	15.3 (10.9)
Fasting glucose at inclusion (mmol/l)	4.6 (0.4)	4.7 (0.6)
HOMA-IR at inclusion	3.3 (2.6)	3.2 (2.3)
2-h glucose (mmol/l)	5.5 (1.5)	5.7 (1.7)
Systolic blood pressure (mm Hg)	119 (12)	117 (12)
Diastolic blood pressure (mm Hg)	75 (13)	73 (10)
Metformin at conception (n, %)	42 (32.8)	41 (30.1)
GDM at inclusion (n, %)	8 (6.3)	10 (7.4)

Abbreviations: BMI = body mass index, PCOS = polycystic ovary syndrome, PCO = polycystic ovaries, OA = oligo- and amennorhea, HA = hyperandrogenism, IVF = *in vitro* fertilization, ICSI = intracytoplasmic sperm injection, HOMA-IR = homeostatic model assessment of insulin resistance, GDM = gestational diabetes mellitus. Continuous variables are presented as mean (SD) and categorical variables as number (%) of participants.

For peer review only

b) Baseline characteristics in the whole population of participants using metformin or no metformin at conception

	Metformin at conception (n=83)	No metformin at conception (n=181)
Age (years)	29.8 (4.3)	29.0 (4.4)
Weight (kg)	82.3 (18.0)	80.3 (19.2)
BMI (kg/m^2)	29.3 (6.6)	28.9 (7.3)
PCOS phenotype	n=77	n=151
A (PCO+OA+HA) (n, %)	45 (58.4)	87 (57.6)
B (OA+HA) (n, %)	3 (3.9)	4 (2.7)
C (PCO+HA) (n, %)	7 (9.1)	18 (11.9)
D (PCO+OA) (n, %)	22 (28.6)	42 (27.8)
hyperandrogenic (n, %)	55 (71.4)	109 (72.2)
normo-androgenic (n, %)	22 (28.6)	42 (27.8)
Mode of conception $^{p=0.003}$	n=83	n=181
Spontaneous (n, %)	48 (57.8)	106 (58.6)
Clomiphene citrate (n, %)	24 (28.9)	40 (22.1)
IVF/ICSI (n, %)	7 (8.4)	35 (19.3)
Other (n, %)	4 (4.8)	0 (0)
Fasting insulin at inclusion (µIU/ml)	15.3 (8.8)	15.7 (11.8)
Fasting glucose at inclusion (mmol/l) $^{p=0.016}$	4.7 (0.6)	4.6 (0.5)
HOMA-IR at inclusion	3.2 (1.9)	3.2 (2.7)
2-h glucose (mmol/l)	5.8 (1.8)	5.5 (1.5)
Systolic blood pressure (mm Hg)	119 (13)	118 (12)
Diastolic blood pressure (mm Hg)	75 (15)	73 (9)
GDM at inclusion (n, %)	10 (13.0)	8 (5.3)

Abbreviations: BMI = body mass index, PCOS = polycystic ovary syndrome, PCO = polycystic ovaries, OA = oligo- and amennorhea, HA = hyperandrogenism, IVF = *in vitro* fertilization, ICSI = intracytoplasmic sperm injection, HOMA-IR = homeostatic model assessment of insulin resistance, GDM = gestational diabetes mellitus. Continuous variables are presented as mean (SD) and categorical variables as number (%) of participants.

For peer review only

c) Baseline characteristics of the participants in the metformin group using metformin or no metformin at conception

	Metformin at conception (n=42)	No metformin at conception (n=86)
Age (years)	30.4 (4.2)	29.1 (4.6)
Weight (kg)	83.3 (18.8)	81.8 (19.1)
BMI (kg/m^2)	29.5 (6.7)	29.3 (6.6)
PCOS phenotype	n=40	n=72
A (PCO+OA+HA) (n, %)	23 (57.5)	42 (58.3)
B (OA+HA) (n, %)	1 (2.5)	2 (2.8)
C (PCO+HA) (n, %)	5 (12.5)	9 (12.5)
D (PCO+OA) (n, %)	11 (27.5)	19 (26.4)
hyperandrogenic (n, %)	29 (72.5)	53 (73.6)
normo-androgenic (n, %)	11 (27.5)	19 (26.4)
Mode of conception $^{p=0.003}$	n=40	n=86
Spontaneous (n, %)	23 (54.8)	53 (61.6)
Clomiphene citrate (n, %)	15 (35.7)	21 (24.4)
IVF/ICSI (n, %)	4 (9.5)	12 (14.0)
Other (n, %)	0 (0)	0 (0)
Fasting insulin at inclusion (µIU/ml)	15.9 (9.6)	15.7 (11.7)
Fasting glucose at inclusion (mmol/l)	4.7 (0.5)	4.6 (0.4)
HOMA-IR at inclusion	3.3 (2.2)	3.2 (2.8)
2-h glucose (mmol/l)	5.6 (1.7)	5.5 (1.5)
Systolic blood pressure (mm Hg)	120 (13)	119 (12)
Diastolic blood pressure (mm Hg)	76 (18)	74 (9)
GDM at inclusion (n, %)	5 (12.5)	3 (4.2)

Abbreviations: BMI = body mass index, PCOS = polycystic ovary syndrome, PCO = polycystic ovaries, OA = oligo- and amennorhea, HA = hyperandrogenism, IVF = *in vitro* fertilization, ICSI = intracytoplasmic sperm injection, HOMA-IR = homeostatic model assessment of insulin resistance, GDM = gestational diabetes mellitus. Continuous variables are presented as mean (SD) and categorical variables as number (%) of participants.

For peer review only

d) Baseline characteristics of the participants using metformin both at conception and during pregnancy or no metformin at all

	Metformin at conception and metformin during pregnancy (n=42)	No metformin at conception and placebo during pregnancy (n=95)
Age (years)	30.4 (4.2)	29.0 (4.3)
Weight (kg)	83.3 (18.8)	79.0 (19.4)
BMI (kg/m^2)	29.5 (6.7)	28.6 (7.9)
PCOS phenotype	n=40	n=79
A (PCO+OA+HA) (n, %)	23 (57.5)	45 (57.0)
B (OA+HA) (n, %)	1 (2.5)	2 (2.5)
C (PCO+HA) (n, %)	5 (12.5)	9 (11.4)
D (PCO+OA) (n, %)	11 (27.5)	23 (29.1)
hyperandrogenic (n, %)	29 (72.5)	56 (70.9)
normo-androgenic (n, %)	11 (27.5)	23 (24.2)
Mode of conception $^{p=0.048}$	n=42	n=95
Spontaneous (n, %)	23 (54.8)	53 (55.8)
Clomiphene citrate (n, %)	15 (35.7)	19 (20.0)
IVF/ICSI (n, %)	4 (9.5)	23 (24.2)
Other (n, %)	0 (0)	0 (0)
Fasting insulin at inclusion (µIU/ml)	15.9 (9.6)	15.7 (12.1)
Fasting glucose at inclusion (mmol/l)	4.7 (0.5)	4.6 (0.5)
HOMA-IR at inclusion	3.3 (2.2)	3.2 (2.5)
2-h glucose (mmol/l)	5.6 (1.7)	5.6 (1.6)
Systolic blood pressure (mm Hg)	120 (13)	117 (12)
Diastolic blood pressure (mm Hg)	76 (18)	72 (9)
GDM at inclusion (n, %)	5 (12.5)	5 (6.3)

Abbreviations: BMI = body mass index, PCOS = polycystic ovary syndrome, PCO = polycystic ovaries, OA = oligo- and amennorhea, HA = hyperandrogenism, IVF = *in vitro* fertilization, ICSI = intracytoplasmic sperm injection, HOMA-IR = homeostatic model assessment of insulin resistance, GDM = gestational diabetes mellitus. Continuous variables are presented as mean (SD) and categorical variables as number (%) of participants.

For peer review only

e) Baseline characteristics of hyperandrogenic and normo-androgenic participants

	Hyperandrogenic PCOS phenotypes (n=164)	Normoandrogenic PCOS phenotypes (n=64)
Age (years)	29.5 (4.6)	28.9 (4.0)
Weight (kg) $^{p=0.034}$	81.8 (18.2)	76.1 (18.2)
BMI (kg/m^{2} $^{p=0.015}$	29.5 (6.9)	27.0 (7.0)
Mode of conception	n=164	n=64
Spontaneous (n, %)	94 (57.4)	32 (50.0)
Clomiphene citrate (n, %)	43 (26.2)	20 (31.2)
IVF/ICSI (n, %)	24 (14.6)	11 (17.2)
Other (n, %)	3 (1.8)	1 (1.6)
Fasting insulin at inclusion (µIU/ml) $^{p=0.029}$	16.5 (12.1)	13.0 (6.7)
Fasting glucose at inclusion (mmol/l)	4.6 (0.5)	4.6 (0.6)
HOMA-IR at inclusion $^{p=0.034}$	3.4 (2.7)	2.7 (1.5)
2-h glucose (mmol/l)	5.6 (1.6)	5.1 (1.3)
Systolic blood pressure (mm Hg)	118 (12)	119 (11)
Diastolic blood pressure (mm Hg)	74 (13)	73 (9)
GDM at inclusion (n, %)	16 (9.8)	5 (6.3)

Abbreviations: BMI = body mass index, IVF = *in vitro* fertilization, ICSI = intracytoplasmic sperm injection, HOMA-IR = homeostatic model assessment of insulin resistance, GDM = gestational diabetes mellitus. Continuous variables are presented as mean (SD) and categorical variables as number (%) of participants.

For peer review only

Supplementary Table 2. Pregnancy complications in women with PCOS according to treatment with metformin or placebo during pregnancy

	Metformin (n=128)	Placebo (n=136)
Pregnancy-induced hypertension (n, %)	7 (5.5)	8 (5.9)
Preeclampsia (n, %)	10 (7.8)	5 (3.7)
Late miscarriage/preterm delivery (n, %)	4 (3.1)	16 (11.8)

Data presented as number (%) of participants.

For peer review only

Supplementary Table 3. Correlations between PROK1 level at pregnancy week 19 and BMI, HOMA-IR, fasting insulin, testosterone and androstenedione levels at inclusion and at pregnancy week 19.

	r-value	p-value	r-value	p-value
	metformin group		placebo group	
BMI at inclusion	-0.091	0.308	-0.013	0.881
BMI at week 19	-0.112	0.207	0.022	0.800
Fasting insulin at inclusion	0.097	0.312	0.017	0.856
Fasting insulin at week 19	0.075	0.432	-0.044	0.636
HOMA-IR at inclusion	0.090	0.345	0.023	0.809
HOMA-IR at week 19	0.085	0.377	-0.065	0.494
Testosterone at inclusion	0.050	0.602	0.119	0.205
Testosterone at week 19	0.123	0.203	-0.057	0.544
Androstenedione at inclusion	0.018	0.850	0.089	0.346
Androstenedione at week 19	0.087	0.308	-0.079	0.881

Abbreviations: BMI = body mass index, HOMA-IR = homeostatic model assessment of insulin resistance

For peer review only

Supplementary material

All participants in the Pilot and PregMet 1 studies met the Rotterdam criteria (retrospectively evaluated in the Pilot study, as it was conducted prior to the establishment of the Rotterdam criteria), additional inclusion criteria were: age 18-45 years, gestational age between weeks 5-12, and a singleton viable fetus shown on ultrasonography. Exclusion criteria were alanine aminotransferase higher than 90 nmol/L, serum creatinine higher than 139 mmol/L, known alcohol abuse, previously diagnosed diabetes mellitus or fasting serum glucose higher than at inclusion, treatment with oral glucocorticoids, or use of drugs known to interfere with metformin. Randomization to metformin or placebo was stratified according to metformin use at conception. All participants were assigned in a double-blind manner either to metformin or placebo group. The participants received 850 mg (Pilot study) or 1000 mg (PregMet 1 study) metformin or placebo twice daily. Women who used metformin at conception had a "wash-out" period of ≥7 days before inclusion. All the participants received a 1mg tablet of folate daily, and one daily multivitamin tablet containing: vitamin A 800 mg, vitamin B_1 1.4 mg, vitamin B_2 1.6 mg, vitamin B_6 2 mg, vitamin B_{12} 1 mg, folic acid 200 mg, niacin 18 mg, pantotenic acid 6 mg, vitamin C 60 mg, vitamin D 5 mg, vitamin E 10 mg, Fe^{2+} 14 mg, Zn^{+} 15 mg, Cu^{2+} 2 mg, iodine 150 mg, Mn^{2+} 2.5 mg, Cr^{+} 50 mg, and Se^{+} 50 mg (Vitaplex^{W}; Alpharma AS, Norway).

Gestational diabetes mellitus was diagnosed as fasting plasma glucose higher than 7 mmol/L and/or 2-h serum glucose higher than 7.8 mmol/L after an OGTT (75 g glucose dissolved in 300 ml water). Pregnancy-induced hypertension was diagnosed as blood pressure of 140/90 mmHg or higher, measured after 10 minutes rest at least at two occasions. Preeclampsia was diagnosed as blood pressure of 140/90 mmHg or higher, measured on two occasions after pregnancy week 20 and albuminuria of at least 2 dipsticks on one occasion or 1 dipstick on two occasions. Late miscarriage was defined as pregnancy loss between week 13 and week 22 and 6 days, preterm delivery was defined as delivery between week 23 and week 36 and 6 days, including spontaneous deliveries, induced vaginal deliveries, and operative deliveries for medical indications.

For peer review only

BMJ Open

Maternal serum levels of prokineticin-1 related to pregnancy complications and metformin use in women with polycystic ovary syndrome: a post-hoc analysis of two prospective, randomized, placebo-controlled trials

Journal:	*BMJ Open*
Manuscript ID	bmjopen-2023-073619.R3
Article Type:	Original research
Date Submitted by the Author:	25-Oct-2023
Complete List of Authors:	Ujvari, Dorina; Karolinska Institute, Department of Women's and Children's Health; Karolinska Institute, Department of Microbiology, Tumor and Cell Biology; National Pandemic Centre; Centre for Translational Microbiome Research<br>Trouva, Anastasia; Karolinska Institute, Department of Molecular Medicine and Surgery<br>Linden , Angelica; Karolinska Institute; Karolinska University Hospital, Department of Gynecology and Reproductive Medicine<br>Vanky, Eszter; Norwegian University of Science and Technology, Department of Clinical and Molecular Medicine; St Olav's University Hospital, Department of Gynaecology and Obstetrics
**Primary Subject Heading**:	Obstetrics and gynaecology
Secondary Subject Heading:	Reproductive medicine
Keywords:	Maternal medicine < OBSTETRICS, Hypertension < CARDIOLOGY, Reproductive medicine < GYNAECOLOGY, Subfertility < GYNAECOLOGY

SCHOLARONE™
Manuscripts

I, the Submitting Author has the right to grant and does grant on behalf of all authors of the Work (as defined in the below author licence), an exclusive licence and/or a non-exclusive licence for contributions from authors who are: i) UK Crown employees; ii) where BMJ has agreed a CC-BY licence shall apply, and/or iii) in accordance with the terms applicable for US Federal Government officers or employees acting as part of their official duties; on a worldwide, perpetual, irrevocable, royalty-free basis to BMJ Publishing Group Ltd ("BMJ") its licensees and where the relevant Journal is co-owned by BMJ to the co-owners of the Journal, to publish the Work in this journal and any other BMJ products and to exploit all rights, as set out in our licence.

The Submitting Author accepts and understands that any supply made under these terms is made by BMJ to the Submitting Author unless you are acting as an employee on behalf of your employer or a postgraduate student of an affiliated institution which is paying any applicable article publishing charge ("APC") for Open Access articles. Where the Submitting Author wishes to make the Work available on an Open Access basis (and intends to pay the relevant APC), the terms of reuse of such Open Access shall be governed by a Creative Commons licence – details of these licences and which Creative Commons licence will apply to this Work are set out in our licence referred to above.

Other than as permitted in any relevant BMJ Author's Self Archiving Policies, I confirm this Work has not been accepted for publication elsewhere, is not being considered for publication elsewhere and does not duplicate material already published. I confirm all authors consent to publication of this Work and authorise the granting of this licence.

For peer review only

Maternal serum levels of prokineticin-1 related to pregnancy complications and metformin use in women with polycystic ovary syndrome: a post-hoc analysis of two prospective, randomized, placebo-controlled trials

Dorina Ujvari[1,2], Anastasia Trouva[3], Angelica Lindén Hirschberg[1,4#], Eszter Vanky[5,6#]

[1]Department of Women's and Children's Health, Karolinska Institute, Solna, Sweden

[2]Department of Microbiology, Tumor and Cell Biology, National Pandemic Centre, Centre for Translational Microbiome Research, Karolinska Institute, Solna, Sweden

[3]Department of Molecular Medicine and Surgery, Karolinska Institute, Stockholm, Sweden

[4]Department of Gynecology and Reproductive Medicine, Karolinska University Hospital, Solna, Sweden

[5]Department of Clinical and Molecular Medicine, Norwegian University of Science and Technology, Trondheim, Norway

[6]Department of Gynaecology and Obstetrics, St Olav's University Hospital, Trondheim, Norway

[#] These authors contributed equally to the work.

Corresponding author:
Dorina Ujvari, PhD
Department of Women's and Children's Health,
Karolinska Institutet,
SE-171 64 Stockholm, Sweden
E-mail: dorina.ujvari@ki.se

For peer review only

Abstract

Objective

Serum prokineticin-1 (s-PROK1) in the second and third trimester of pregnancy is positively correlated to preeclampsia, intrauterine growth restriction and preterm delivery. Women with polycystic ovary syndrome (PCOS) are prone to these adverse pregnancy outcomes. However, the contribution of PROK1 to the development of pregnancy complications and the effect of metformin and hyperandrogenism on s-PROK1 in PCOS have not been studied previously.

Design

This work is a post-hoc analysis of two prospective, randomized, placebo-controlled trials.

Setting

Pregnant women with PCOS were included from 11 study centres in Norway.

Participants

From 313 women, 264 participated in the present study after exclusions due to dropouts or insufficient serum samples.

Intervention

Women with PCOS were randomly assigned to metformin or placebo, from first trimester to delivery.

Primary and secondary outcome measures

S-PROK1 were analyzed using enzyme-linked immunosorbent assay at gestational week 19 and related to pregnancy complications, fasting insulin levels, HOMA-IR, testosterone, or androstenedione levels, metformin use, PCOS phenotype and hyperandrogenism.

Results

Maternal s-PROK1 in the second trimester did not predict pregnancy-induced hypertension, preeclampsia or late miscarriage/preterm delivery in women with PCOS. However, s-PROK1 was lower in women who used metformin before inclusion, both in those randomized to metformin and to placebo, compared to those who did not. S-PROK1 was also lower in those who used metformin from inclusion, or in those who used metformin both at conception and during pregnancy compared to those who did

For peer review only

not use metformin at all. S-PROK1 was lower in hyperandrogenic compared to normo-androgenic women with PCOS.

Conclusions

Maternal s-PROK1 in the second trimester did not predict pregnancy complications in PCOS. Those who used metformin at conception and/or during pregnancy had lower s-PROK1. PCOS women with hyperandrogenism exhibited lower s-PROK1 compared with normo-adrogenic phenotypes.

Trial registration

The clinical trials were registered at www.clinicaltrials.gov as NCT03259919 and NCT00159536.

For peer review only

Strengths and limitations of this study

- This study is a post-hoc analysis of 2 randomized, double-blinded, placebo controlled, multi-center trials.
- The study includes 264 well-characterized pregnant women with PCOS.
- The number of pregnancy complications was limited, potentially leading to type II errors.
- Due to the broad timeframe of sampling in the first trimester and the lack of predictive value at week 32 and 36, s-PROK1 was measured only at week 19.

Keywords: PCOS, pregnancy, prokineticin-1, metformin, preeclampsia, placenta

Abbreviations: PROK1, prokineticin-1; s-PROK1, serum level of prokineticin-1; PCOS, polycystic ovary syndrome; HA, hyperandrogenism; PCO, polycystic ovaries; OA, oligo-amenorrhea; IUGR, intrauterine growth restriction; BMI, body mass index; HOMA-IR, homeostatic model assessment for insulin resistance

Word count: 1980

Introduction

Polycystic ovary syndrome (PCOS) is the most common endocrine disorder in women of reproductive age. Its prevalence is 10-17% based on at least two of the three features of PCOS (oligo-amenorrhea, hyperandrogenism and polycystic ovaries) according to the Rotterdam criteria (1). Approximately 70% of these patients have insulin resistance and compensatory hyperinsulinemia (2). PCOS is closely related to subfertility and pregnancy complications, including miscarriage, gestational diabetes, preeclampsia, intrauterine growth restriction (IUGR) and preterm delivery (3,4). Hyperinsulinemia and hyperandrogenism might contribute to the development of these complications (5,6).

Prokineticin-1 (PROK1) is a pleiotropic factor secreted by endocrine glands, with a broad range of biological functions. In the female reproductive system, it is expressed in the endometrium, ovaries, adrenal glands and placenta (7,8), and seems to be a pivotal actor at all the different stages of the gestation, from follicular development to parturition. PROK1 is significantly increased in first trimester decidua, compared with non-pregnant endometrium and controls trophoblast migration and invasion into the decidua and angiogenesis in the developing placenta (9,10). The placenta is thought to be the main source of PROK1 in the maternal circulation (11,12). In uncomplicated pregnancies, serum level of PROK1 (s-PROK1) increases 5-fold in the first trimester compared to non-pregnant state (13), and promotes the proliferation of anchoring cytotrophoblasts and formation of trophoblastic plugs in the maternal spiral arteries (11,14). After gestational week 10-12, PROK1 levels decline, facilitating the transformation of trophoblasts into an invasive phenotype, the remodeling of the maternal spiral arteries and the increase of placental oxygen tension to promote placental angiogenesis in order to supply the growing fetus (11,13). Excess expression of PROK1 was demonstrated to cause poor invasion of extravillous trophoblasts in the first trimester. This potentially leads to imperfect trophoblastic plugs resulting in early pregnancy loss, or in less severe cases to insufficient remodeling of maternal spiral arteries, and consequently to impaired placental development. The relatively hypoxic milieu in the abnormal placenta leads to the up-regulation of several hypoxia-regulated factors, among others PROK1 (10). Dysregulation of PROK1 is associated with preeclampsia, IUGR and preterm delivery (13,14,15,16). We have previously shown

For peer review only

that insulin up-regulates PROK1 in decidualizing endometrial stromal cells *in vitro*, and androgens potentiate this enhancement (17,18). Despite hyperinsulinemia/hyperandrogenism and an increased occurrence of these pregnancy complications, the role of PROK1 has not been studied in PCOS.

The administration of metformin, an insulin sensitizer, in the treatment of non-pregnant PCOS women is well established (19). Evidence shows its usefulness in reducing late miscarriages and preterm deliveries, however, its efficacy on other complications is debated and the mechanism of action is not fully understood (20,21,22,23).

The primary objective of this study was to investigate if s-PROK1 predicts pregnancy-induced hypertension, preeclampsia, and late miscarriage/preterm delivery. We hypothesized that metformin treatment was associated with lower, and hyperandrogenism with higher s-PROK1 in the second trimester.

Materials and methods

Study design

This work is a post-hoc analysis of two prospective, randomized, placebo-controlled trials, investigating the effect of metformin on pregnancy complications in women with PCOS. 264 women participated with analyzed serum samples in the present study, 128 and 136 in the metformin and placebo group, respectively (Figure 1). The clinical studies (20,21) are described in the Supplementary material.

Enzyme-linked immunosorbent assay

Serum samples, collected at gestational week 19, were analyzed by a PROK1 ELISA kit (Thermo Fischer Scientific), in 1:4 dilution. Absorbance of the samples was measured in duplicates at 450 and 550 nm on a Multiscan FC photometer, and A_{450}-A_{550} values were used to correct for optical imperfections in the microplate. To plot the standard curves, the four-parameter logistic curve fit was applied. The limit of detection (LOD) was 13.72 pg/ml. Values below the detection limit were replaced by 0.5xLOD. The intra-assay and inter-assay coefficients of variations were <10% and <12%, respectively.

For peer review only - http://bmjopen.bmj.com/site/about/guidelines.xhtml

Patient and public involvement statement

None.

Statistical analysis

Statistical procedures were performed using SPSS by IBM Version 25 and Statistica 14.0 TIBCO software Inc. Differences in baseline characteristics were analyzed using unpaired T-test and Fischer's exact test. S-PROK1 was positively skewed and therefore log-transformed. Logistic regression analysis was used to analyze whether s-PROK1 predicts pregnancy complications. Differences of s-PROK1 between subgroups were analyzed by median test, followed by pairwise median test with Bonferroni correction, in case of statistical significance. Correlations between s-PROK1 and other variables were evaluated using Spearman`s rank order correlation test. $P<0.05$ was considered as statistically significant.

Results

Study population

Baseline characteristics of the women in the metformin and the placebo groups were comparable (Supplementary Table 1). Baseline characteristics were compared in additional subgroups (Supplementary Table 1). Weight, body mass index (BMI), fasting insulin levels and homeostatic model assessment for insulin resistance (HOMA-IR) were higher in hyperandrogenic compared to normo-androgenic participants ($p=0.034$, $p=0.015$, $p=0.029$ and $p=0.034$, respectively). The mode of conception was significantly different between subgroups used and not used metformin during conception ($p=0.003$) and between women who took metformin both at conception and during pregnancy compared to those who did not use metformin at all ($p=0.048$).

Pregnancy complications

Pregnancy complications are listed in Supplementary Table 2. S-PROK1 did not predict pregnancy-induced hypertension (n=15) (OR 0.95, 95% CI 0.32–2.74, $p=0.92$), preeclampsia (n=15) (OR 0.47, 95% CI 0.14–1.35, $p=0.17$) or late miscarriage/preterm

For peer review only - http://bmjopen.bmj.com/site/about/guidelines.xhtml

delivery (n=20) (OR 0.62, 95% CI 0.24–1.56, p=0.32).

PROK1 and metformin use at conception, during pregnancy, and both at conception and during pregnancy

S-PROK1 was lower in women who used metformin at conception (median=6.86, 95% CI 6.86-11.22) compared to those who did not (median=19.53, 95% CI 7.00-30.83) (p=0.012), regardless of later randomization to metformin or placebo. S-PROK1 was not different in women randomized to metformin (median=6.86, 95% CI 6.86-20.51) or placebo (median=17.75, 95% CI 7.00-30.83) during pregnancy (p=0.166) (Figure 2b). Median test showed a statistically significant difference between the four subgroups of no metformin use/metformin use at conception combined with metformin/placebo use during pregnancy (p=0.020). Pairwise median test revealed that s-PROK1 was lower in women who used metformin both at conception and during pregnancy (median=6.86, 95% CI 6.86-18.00), compared to those who used metformin during pregnancy (median=19.76, 95% CI 9.60-46.46) (p=0.008, $p_{adjusted}$=0.040) (Figure 3). S-PROK1 was lower in women who took metformin both at conception and during pregnancy compared to those who did not use metformin at all (median=19.53, 95% CI 7.00-40.36) (p=0.001, $p_{adjusted}$=0.005) (Figure 3). The difference between those who used metformin at conception and during pregnancy compared with those who used metformin at conception and placebo during pregnancy (median=6.86, 95% CI 6.86-68.70) did not reach the level of statistical significance (p=0.060, $p_{adjusted}$=0.301) (Figure 3). There was no statistically significant difference between women who did not use compared with those who used metformin at conception in the placebo group (p=0.577, $p_{adjusted}$=1) and between women who did not use metformin at conception, but took metformin compared with women who took placebo during pregnancy (p=0.832, $p_{adjusted}$=1) (Figure 3).

PCOS phenotypes

Differences in s- PROK1 were also analyzed in subgroups stratified according to PCOS phenotypes. Patients from the Pilot study (n=37) were not included in this analysis, as they were diagnosed according to the NIH criteria, and due to the lack of data on the presence or absence of polycystic ovaries, those fulfilling all three criteria and hyperandrogenism-oligo-amenorrhea groups could not been distinguished. In PregMet1 samples, s-PROK1 was not significantly different between PCOS

phenotypes A (HA+PCO+OA), B (HA+PCO), C (HA+OA) and D (PCO+OA) (p=0.177) (data not shown). S-PROK1 was lower in hyperandrogenic (median=6.86, 95% CI 6.86-9.69) compared to normo-androgenic women (median=6.86, 95% CI 7.00-88.69) (p=0.040) (Figure 4).

Correlations

S-PROK1 showed no correlation with BMI, fasting insulin levels, HOMA-IR, testosterone, or androstenedione levels either at inclusion or at week 19 of gestation (Supplementary Table 3).

Discussion

The primary aim of this study was to investigate if s-PROK1 predicts pregnancy-induced hypertension, preeclampsia, and late miscarriage/preterm delivery in women with PCOS. Previously, s-PROK1 was reported to be higher in the second and third trimesters in preeclamptic and IUGR pregnancies compared to uncomplicated pregnancies (13,14). We and others have also demonstrated that PROK1 restricts the migration and invasion of trophoblast cells *in vitro*, suggesting that elevated s-PROK1 impairs embryo implantation and placentation (13,18). However, s-PROK1 in the second trimester did not predict pregnancy-induced hypertension or preeclampsia in our women with PCOS. It has also been demonstrated that PROK1 is up-regulated in the myometrium and the placenta during labor, suggesting a role for PROK1 in the induction of term and preterm labor (15,16). However, in this study, s-PROK1 did not predict late miscarriages/preterm deliveries in pregnant women with PCOS, but we cannot exclude that a limited number of cases might have influenced our results.

We also investigated the impact of metformin on s-PROK1 and found lower levels by metformin use at conception, both with and without metformin use after inclusion, suggesting that metformin intake, especially in early pregnancy, might have a lowering effect on s-PROK1. However, participants were not randomized at this timepoint and the lower s-PROK1 at week 19 might reflect that those who used metformin at conception differed from those who did not. It is known that metformin decreases insulin levels, although the treatment does not seem to prevent gestational diabetes in

For peer review only - http://bmjopen.bmj.com/site/about/guidelines.xhtml

PCOS women (20,21,23). We recently demonstrated that insulin upregulates PROK1 in decidualizing endometrial stromal cells *in vitro* (18). However, in this study, we found no correlation between s-PROK1 and insulin levels.

A possible association between s-PROK1 and hyperandrogenism was also investigated. In contrast to our hypothesis, s-PROK1 was lower in women with hyperandrogenic phenotypes compared to normo-androgenic phenotype. However, there was no correlation between s-PROK1 and androgen levels at inclusion and week 19. Direct and indirect evidence support the role of androgens in the pathogenesis of placenta-related pregnancy complications (24). We have previously showed that androgens potentiate insulin to up-regulate PROK1 in decidualizing endometrial stromal cells *in vitro* (17). Increased maternal serum androgens have been reported in preeclamptic pregnancies (25,26,27,28). PCOS women with pregnancy complications have higher androgen levels than those without (29). It was also shown that trophoblast invasion and placentation are impaired in PCOS compared to controls, and this impairment is associated with insulin resistance and circulating testosterone concentrations (30). However, androgens do not seem to directly contribute to the regulation of s-PROK1, although hyperandrogenism could be indirectly involved.

Strengths and limitations of this study

There are several strengths of the present study including the well-characterized women with PCOS and the randomized, double-blinded placebo-controlled multi-center study design, prospective data sampling and meticulous quality-checked diagnosis control. Some limitations should also be mentioned. There was a slight difference between the two trials regarding the metformin dose, 1700 mg and 2000 mg daily, respectively, which might have affected our results. Since maternal s-PROK1 has a dynamic course during pregnancy, timing of blood sampling for determination of s-PROK1 is crucial. Based on previous studies showing elevated s-PROK1 in the second and third trimester of preeclamptic and IUGR pregnancies, we chose to analyze s-PROK1 in gestational week 19, representing the second trimester. We did not measure the s-PROK1 in serum samples taken at inclusion (between gestational week 5 and 12), as s-PROK1 has been shown to decline remarkably within this period and patients could not have been comparable. S-PROK1 was not measured at gestational week 32 and 36 either, as these late timepoints would not hold predictive values for gestational diabetes, early onset

For peer review only

preeclampsia or late miscarriage. However, we cannot exclude that s-PROK1 should have been determined at several timepoints to study the course of PROK1 for pregnancy complications in PCOS. Furthermore, the number of pregnancy complication cases was limited possibly leading to type II errors.

Conclusions

S-PROK1, in the second trimester, did not predict pregnancy-induced hypertension, preeclampsia, or late miscarriage/preterm delivery in pregnant women with PCOS. Women who used metformin at conception and/or during pregnancy and those with hyperandrogenism compared to normo-androgenism exhibited lower s-PROK1. Further studies are needed to determine the exact role of s-PROK1 in pregnancy complications and the involvement of metformin and hyperandrogenism in the regulation of PROK1 in PCOS.

Acknowledgements

The authors thank Weifa A/S for delivering metformin and placebo tablets free of charge.

Author contributions

EV conceived and designed the original protocol. On the initiative of DU and ALH, the protocol of the present study was written together with EV. DU run the analysis, DU and AT managed the database and analyzed the data with the input from EV and ALH. A statistician performed the statistical analyses. DU and AT drafted the manuscript with input from ALH, EV. All the authors contributed to the article, reviewed and approved the submitted version.

Conflicts of interest

The authors declare no conflicts of interest.

For peer review only - http://bmjopen.bmj.com/site/about/guidelines.xhtml

Funding information

This work was supported by grants from the Liaison Committee between the Central Norway Regional Health Authority and the Norwegian University of Science and Technology, Swedish Research Council (2021-01348), Stockholm County Council (2019-0248) and Åke Wiberg foundation (M18-0114).

Data availability

Data are available upon reasonable request.

Ethics statement

An informed consent was obtained from each patient and the declaration of Helsinki was followed throughout the studies. The Committee for Medical Research Ethics of Health Region IV, Norway and The Norwegian Medicines Agency approved the studies (project numbers: 51–2000 and 145.04) and the supplement application for the current study (reference number 2010/3115 (145-04)). The clinical trials were registered at www.clinicaltrials.gov as NCT03259919 (The pilot study) and NCT00159536 (The PregMet1 study).

References

1. Bozdag G, Mumusoglu S, Zengin D, et al. The prevalence and phenotypic features of polycystic ovary syndrome: a systematic review and meta-analysis. *Hum Reprod* 2016;31(12):2841-55. doi: 10.1093/humrep/dew218 [published Online First: 20160922]
2. March WA, Moore VM, Willson KJ, et al. The prevalence of polycystic ovary syndrome in a community sample assessed under contrasting diagnostic criteria. *Hum Reprod* 2010;25(2):544-51. doi: 10.1093/humrep/dep399 [published Online First: 20091112]
3. Hirschberg AL. Polycystic ovary syndrome, obesity and reproductive implications. *Womens Health (Lond)* 2009;5(5):529-40; quiz 41-2. doi: 10.2217/whe.09.39 [published Online First: 2009/08/26]

For peer review only

4. Franks S. Polycystic ovary syndrome. *N Engl J Med* 1995;333(13):853-61. doi: 10.1056/NEJM199509283331307
5. Azziz R, Carmina E, Chen Z, et al. Polycystic ovary syndrome. *Nat Rev Dis Primers* 2016;2:16057. doi: 10.1038/nrdp.2016.57 [published Online First: 20160811]
6. Dumesic DA, Oberfield SE, Stener-Victorin E, et al. Scientific Statement on the Diagnostic Criteria, Epidemiology, Pathophysiology, and Molecular Genetics of Polycystic Ovary Syndrome. *Endocr Rev* 2015;36(5):487-525. doi: 10.1210/er.2015-1018
7. Battersby S, Critchley HO, Morgan K, et al. Expression and regulation of the prokineticins (endocrine gland-derived vascular endothelial growth factor and Bv8) and their receptors in the human endometrium across the menstrual cycle. *The Journal of clinical endocrinology and metabolism* 2004;89(5):2463-9. doi: 10.1210/jc.2003-032012
8. Traboulsi W, Brouillet S, Sergent F, et al. Prokineticins in central and peripheral control of human reproduction. *Horm Mol Biol Clin Investig* 2015;24(2):73-81. doi: 10.1515/hmbci-2015-0040
9. Alfaidy N, Brouillet S, Rajaraman G, et al. The Emerging Role of the Prokineticins and Homeobox Genes in the Vascularization of the Placenta: Physiological and Pathological Aspects. *Front Physiol* 2020;11:591850. doi: 10.3389/fphys.2020.591850 [published Online First: 20201112]
10. Alfaidy N, Hoffmann P, Boufettal H, et al. The multiple roles of EG-VEGF/PROK1 in normal and pathological placental angiogenesis. *Biomed Res Int* 2014;2014:451906. doi: 10.1155/2014/451906 [published Online First: 20140515]
11. Hoffmann P, Feige JJ, Alfaidy N. Expression and oxygen regulation of endocrine gland-derived vascular endothelial growth factor/prokineticin-1 and its receptors in human placenta during early pregnancy. *Endocrinology* 2006;147(4):1675-84. doi: 10.1210/en.2005-0912
12. Brouillet S, Hoffmann P, Benharouga M, et al. Molecular characterization of EG-VEGF-mediated angiogenesis: differential effects on microvascular and macrovascular endothelial cells. *Molecular biology of the cell* 2010;21(16):2832-43. doi: 10.1091/mbc.E10-01-0059
13. Hoffmann P, Saoudi Y, Benharouga M, et al. Role of EG-VEGF in human placentation: Physiological and pathological implications. *Journal of cellular and molecular medicine* 2009;13(8B):2224-35. doi: 10.1111/j.1582-4934.2008.00554.x
14. Brouillet S, Murthi P, Hoffmann P, et al. EG-VEGF controls placental growth and survival in normal and pathological pregnancies: case of fetal growth restriction (FGR). *Cellular and molecular life sciences : CMLS* 2013;70(3):511-25. doi: 10.1007/s00018-012-1141-z
15. Catalano RD, Lannagan TR, Gorowiec M, et al. Prokineticins: novel mediators of inflammatory and contractile pathways at parturition? *Mol Hum Reprod* 2010;16(5):311-9. doi: 10.1093/molehr/gaq014 [published Online First: 20100219]
16. Gorowiec MR, Catalano RD, Norman JE, et al. Prokineticin 1 induces inflammatory response in human myometrium: a potential role in initiating term and preterm parturition. *Am J Pathol* 2011;179(6):2709-19. doi: 10.1016/j.ajpath.2011.08.029 [published Online First: 20111006]

For peer review only

17. Ujvari D, Graells Brugalla C, Hirschberg AL. Dihydrotestosterone potentiates insulin to up-regulate prokineticin-1 in decidualizing human endometrial stromal cells. *Journal of cellular and molecular medicine* 2020;24(5):3242-45. doi: 10.1111/jcmm.14923 [published Online First: 20200128]
18. Ujvari D, Jakson I, Oldmark C, et al. Prokineticin 1 is up-regulated by insulin in decidualizing human endometrial stromal cells. *Journal of cellular and molecular medicine* 2018;22(1):163-72. doi: 10.1111/jcmm.13305 [published Online First: 2017/08/07]
19. Teede HJ, Misso ML, Costello MF, et al. Recommendations from the international evidence-based guideline for the assessment and management of polycystic ovary syndrome. *Hum Reprod* 2018;33(9):1602-18. doi: 10.1093/humrep/dey256
20. Vanky E, Salvesen KA, Heimstad R, et al. Metformin reduces pregnancy complications without affecting androgen levels in pregnant polycystic ovary syndrome women: results of a randomized study. *Hum Reprod* 2004;19(8):1734-40. doi: 10.1093/humrep/deh347
21. Vanky E, Stridsklev S, Heimstad R, et al. Metformin versus placebo from first trimester to delivery in polycystic ovary syndrome: a randomized, controlled multicenter study. *The Journal of clinical endocrinology and metabolism* 2010;95(12):E448-55. doi: 10.1210/jc.2010-0853 [published Online First: 2010/10/12]
22. Fornes R, Simin J, Nguyen MH, et al. Pregnancy, perinatal and childhood outcomes in women with and without polycystic ovary syndrome and metformin during pregnancy: a nationwide population-based study. *Reprod Biol Endocrinol* 2022;20(1):30. doi: 10.1186/s12958-022-00905-6 [published Online First: 20220207]
23. Lovvik TS, Carlsen SM, Salvesen O, et al. Use of metformin to treat pregnant women with polycystic ovary syndrome (PregMet2): a randomised, double-blind, placebo-controlled trial. *Lancet Diabetes Endocrinol* 2019;7(4):256-66. doi: 10.1016/S2213-8587(19)30002-6 [published Online First: 20190218]
24. Kumar S, Gordon GH, Abbott DH, et al. Androgens in maternal vascular and placental function: implications for preeclampsia pathogenesis. *Reproduction* 2018;156(5):R155-R67. doi: 10.1530/REP-18-0278 [published Online First: 20181016]
25. Acromite MT, Mantzoros CS, Leach RE, et al. Androgens in preeclampsia. *American journal of obstetrics and gynecology* 1999;180(1 Pt 1):60-3. doi: 10.1016/s0002-9378(99)70150-x [published Online First: 1999/01/23]
26. Serin IS, Kula M, Basbug M, et al. Androgen levels of preeclamptic patients in the third trimester of pregnancy and six weeks after delivery. *Acta obstetricia et gynecologica Scandinavica* 2001;80(11):1009-13.
27. Atamer Y, Erden AC, Demir B, et al. The relationship between plasma levels of leptin and androgen in healthy and preeclamptic pregnant women. *Acta obstetricia et gynecologica Scandinavica* 2004;83(5):425-30. doi: 10.1111/j.0001-6349.2004.00276.x
28. Carlsen SM, Romundstad P, Jacobsen G. Early second-trimester maternal hyperandrogenemia and subsequent preeclampsia: a prospective study. *Acta obstetricia et gynecologica Scandinavica* 2005;84(2):117-21. doi: 10.1111/j.0001-6349.2005.00493.x

For peer review only - http://bmjopen.bmj.com/site/about/guidelines.xhtml

29. Falbo A, Rocca M, Russo T, et al. Changes in androgens and insulin sensitivity indexes throughout pregnancy in women with polycystic ovary syndrome (PCOS): relationships with adverse outcomes. *Journal of ovarian research* 2010;3:23. doi: 10.1186/1757-2215-3-23
30. Palomba S, Russo T, Falbo A, et al. Decidual endovascular trophoblast invasion in women with polycystic ovary syndrome: an experimental case-control study. *The Journal of clinical endocrinology and metabolism* 2012;97(7):2441-9. doi: 10.1210/jc.2012-1100

For peer review only

Figure legends

Figure 1

Flowchart of the original and the secondary studies.

Figure 2

a. Serum levels of PROK1 at pregnancy week 19 in women with PCOS, who took metformin at conception (n=83) compared to those, who did not use metformin at conception (n=181). Values are presented as median, interquartile ranges (P25-P75) and non-outlier ranges (min-max). Open circles represent outliers (1.5*the box-height from the box).

b. Serum levels of PROK1 at pregnancy week 19 in women with PCOS, who took metformin (n=128) compared to those, who took placebo after randomization (n=136). Values are presented as median, interquartile ranges (P25-P75) and non-outlier ranges (min-max). Open circles represent outliers (1.5*the box-height from the box).

Figure 3

Serum levels of PROK1 at pregnancy week 19 in women with PCOS, who did not use metformin/used metformin at conception in the placebo group (n=41 and n=95, respectively) and in the metformin group (n=42 and n=86, respectively). Values are presented as median, interquartile ranges (P25-P75) and non-outlier ranges (min-max). Open circles represent outliers (1.5*the box-height from the box) and the stars represent extremes (3*the box-height from the box).

Figure 4

Serum levels of PROK1 at pregnancy week 19 in women with PCOS and hyperandrogenism (n=164) compared with those without hyperandrogenism (n=64). Values are presented as median, interquartile ranges (P25-P75) and non-outlier ranges (min-max). Open circles represent outliers (1.5*the box-height from the box).

Supplementary material

Description and details of the two prospective, randomized, placebo-controlled trials.

For peer review only - http://bmjopen.bmj.com/site/about/guidelines.xhtml

Figure 1

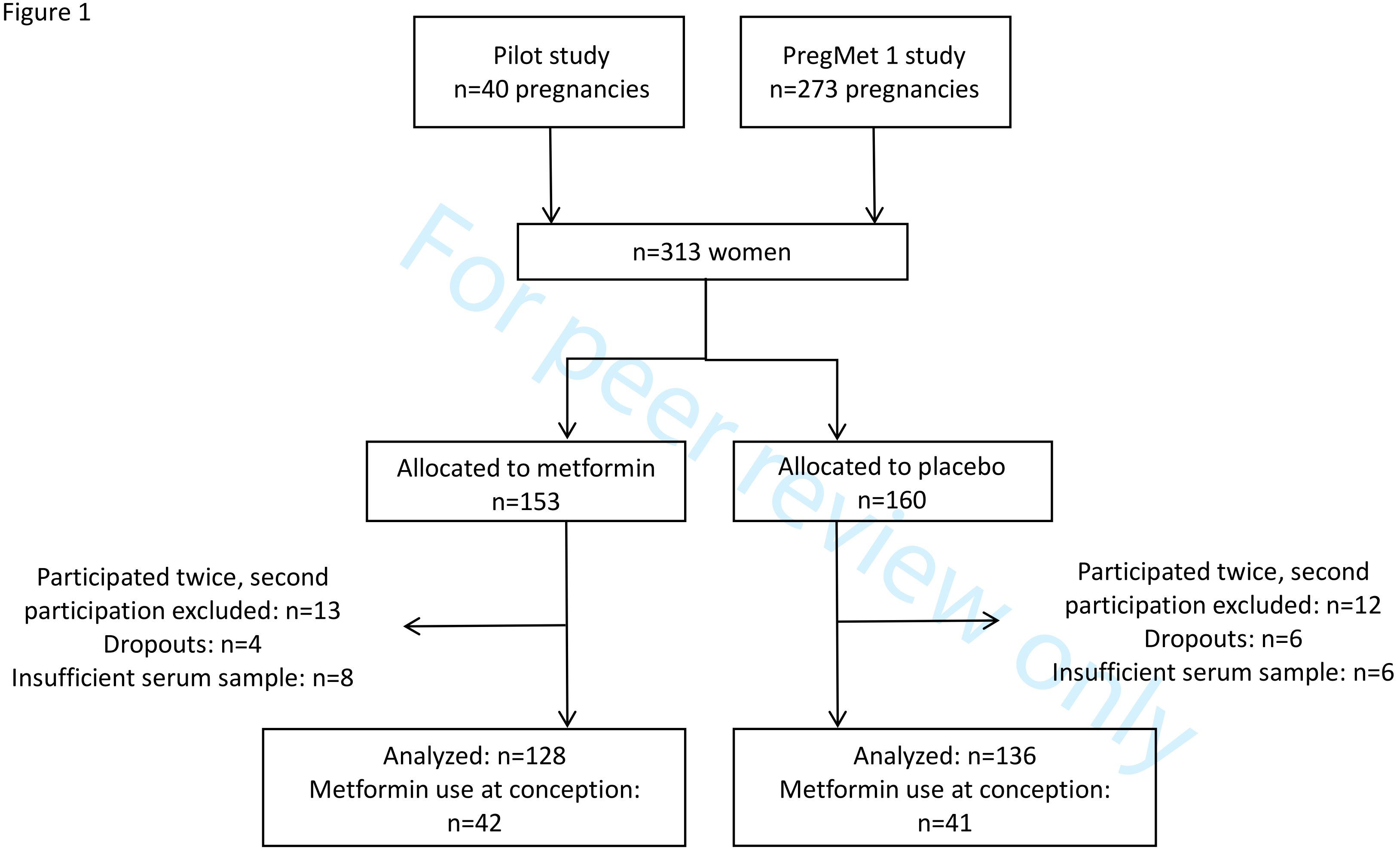

For peer review only

Figure 2

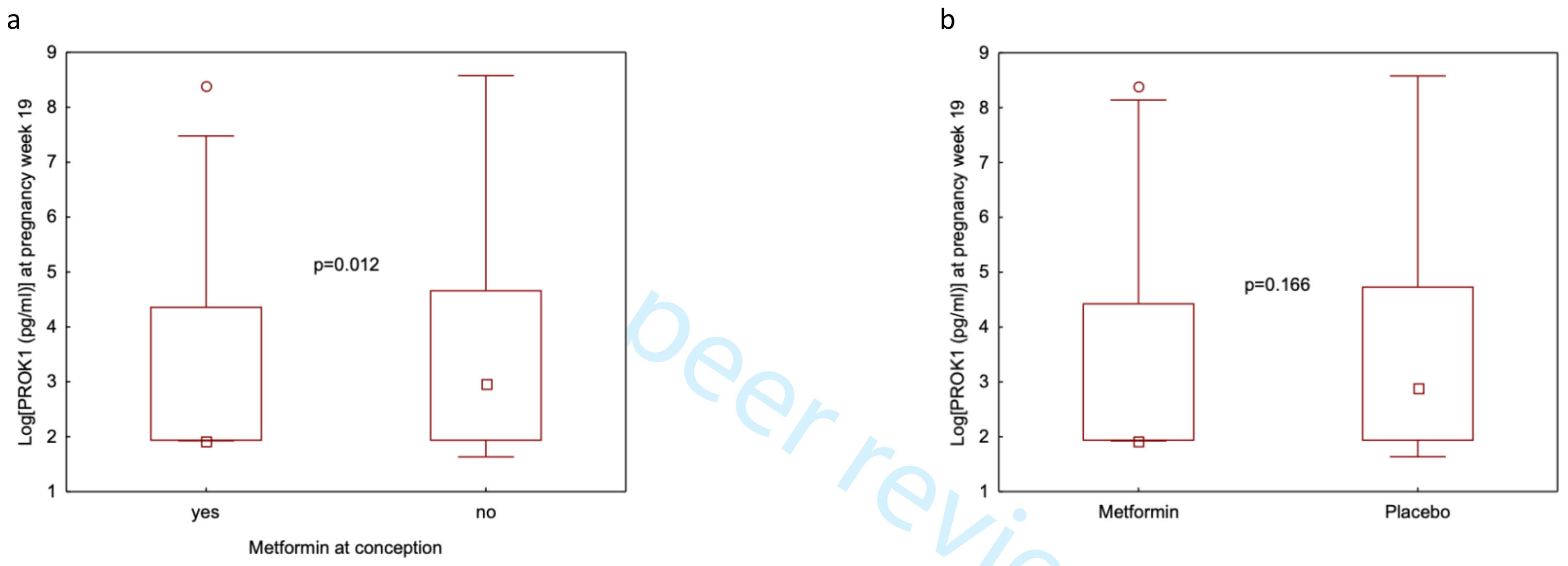

For peer review only

Figure 3

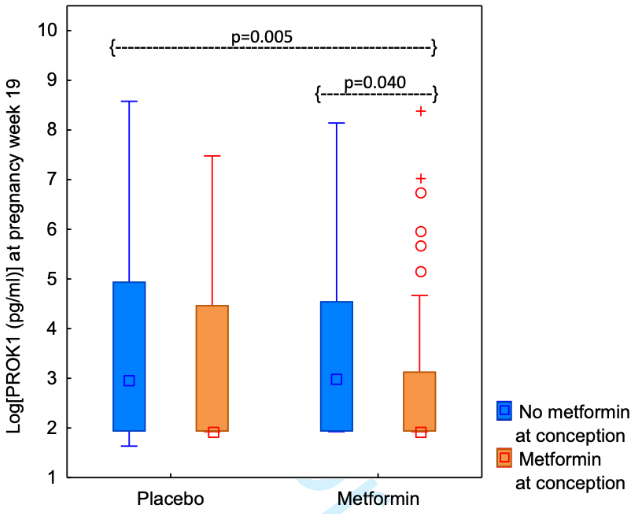

For peer review only

Figure 4

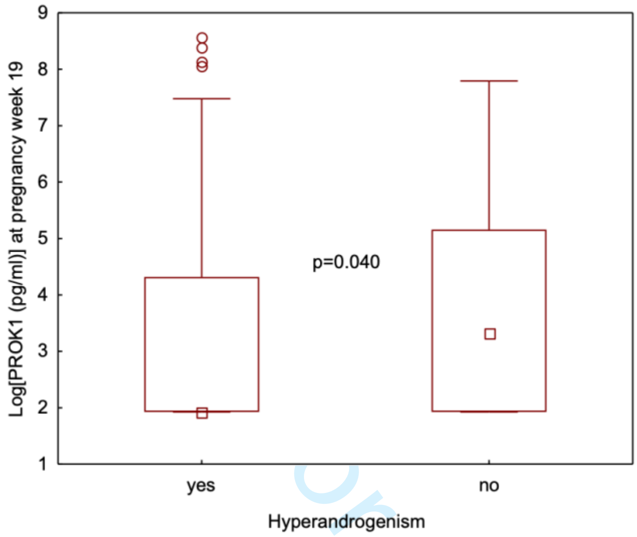

For peer review only

Supplementary Table 1 a-e.

a) Baseline characteristics of the participants

	Metformin (n=128)	Placebo (n=136)
Age (years)	29.5 (4.5)	29.0 (4.3)
Weight (kg)	82.3 (18.9)	79.7 (18.7)
BMI (kg/m^2)	29.4 (6.6)	28.7 (7.5)
PCOS phenotype	n=112	n=116
A (PCO+OA+HA) (n, %)	65 (58.0)	67 (57.8)
B (OA+HA) (n, %)	3 (2.7)	4 (3.4)
C (PCO+HA) (n, %)	14 (12.5)	11 (9.5)
D (PCO+OA) (n, %)	30 (26.8)	34 (29.3)
hyperandrogenic (n, %)	82 (73.2)	82 (70.7)
normo-androgenic (n, %)	30 (26.8)	34 (29.3)
Mode of conception	n=128	n=136
Spontaneous (n, %)	76 (59.4)	78 (57.4)
Clomiphene citrate (n, %)	36 (28.1)	28 (20.6)
IVF/ICSI (n, %)	16 (12.5)	26 (19.1)
Other (n, %)	0 (0)	4 (2.9)
Fasting insulin at inclusion (µIU/ml)	15.7 (11.0)	15.3 (10.9)
Fasting glucose at inclusion (mmol/l)	4.6 (0.4)	4.7 (0.6)
HOMA-IR at inclusion	3.3 (2.6)	3.2 (2.3)
2-h glucose (mmol/l)	5.5 (1.5)	5.7 (1.7)
Systolic blood pressure (mm Hg)	119 (12)	117 (12)
Diastolic blood pressure (mm Hg)	75 (13)	73 (10)
Metformin at conception (n, %)	42 (32.8)	41 (30.1)
GDM at inclusion (n, %)	8 (6.3)	10 (7.4)

Abbreviations: BMI = body mass index, PCOS = polycystic ovary syndrome, PCO = polycystic ovaries, OA = oligo- and amennorhea, HA = hyperandrogenism, IVF = *in vitro* fertilization, ICSI = intracytoplasmic sperm injection, HOMA-IR = homeostatic model assessment of insulin resistance, GDM = gestational diabetes mellitus. Continuous variables are presented as mean (SD) and categorical variables as number (%) of participants.

For peer review only

b) Baseline characteristics in the whole population of participants using metformin or no metformin at conception

	Metformin at conception (n=83)	No metformin at conception (n=181)
Age (years)	29.8 (4.3)	29.0 (4.4)
Weight (kg)	82.3 (18.0)	80.3 (19.2)
BMI (kg/m^2)	29.3 (6.6)	28.9 (7.3)
PCOS phenotype	n=77	n=151
A (PCO+OA+HA) (n, %)	45 (58.4)	87 (57.6)
B (OA+HA) (n, %)	3 (3.9)	4 (2.7)
C (PCO+HA) (n, %)	7 (9.1)	18 (11.9)
D (PCO+OA) (n, %)	22 (28.6)	42 (27.8)
hyperandrogenic (n, %)	55 (71.4)	109 (72.2)
normo-androgenic (n, %)	22 (28.6)	42 (27.8)
Mode of conception $^{p=0.003}$	n=83	n=181
Spontaneous (n, %)	48 (57.8)	106 (58.6)
Clomiphene citrate (n, %)	24 (28.9)	40 (22.1)
IVF/ICSI (n, %)	7 (8.4)	35 (19.3)
Other (n, %)	4 (4.8)	0 (0)
Fasting insulin at inclusion (µIU/ml)	15.3 (8.8)	15.7 (11.8)
Fasting glucose at inclusion (mmol/l) $^{p=0.016}$	4.7 (0.6)	4.6 (0.5)
HOMA-IR at inclusion	3.2 (1.9)	3.2 (2.7)
2-h glucose (mmol/l)	5.8 (1.8)	5.5 (1.5)
Systolic blood pressure (mm Hg)	119 (13)	118 (12)
Diastolic blood pressure (mm Hg)	75 (15)	73 (9)
GDM at inclusion (n, %)	10 (13.0)	8 (5.3)

Abbreviations: BMI = body mass index, PCOS = polycystic ovary syndrome, PCO = polycystic ovaries, OA = oligo- and amennorhea, HA = hyperandrogenism, IVF = *in vitro* fertilization, ICSI = intracytoplasmic sperm injection, HOMA-IR = homeostatic model assessment of insulin resistance, GDM = gestational diabetes mellitus. Continuous variables are presented as mean (SD) and categorical variables as number (%) of participants.

For peer review only

c) Baseline characteristics of the participants in the metformin group using metformin or no metformin at conception

	Metformin at conception (n=42)	No metformin at conception (n=86)
Age (years)	30.4 (4.2)	29.1 (4.6)
Weight (kg)	83.3 (18.8)	81.8 (19.1)
BMI (kg/m^2)	29.5 (6.7)	29.3 (6.6)
PCOS phenotype	n=40	n=72
A (PCO+OA+HA) (n, %)	23 (57.5)	42 (58.3)
B (OA+HA) (n, %)	1 (2.5)	2 (2.8)
C (PCO+HA) (n, %)	5 (12.5)	9 (12.5)
D (PCO+OA) (n, %)	11 (27.5)	19 (26.4)
hyperandrogenic (n, %)	29 (72.5)	53 (73.6)
normo-androgenic (n, %)	11 (27.5)	19 (26.4)
Mode of conception $^{p=0.003}$	n=40	n=86
Spontaneous (n, %)	23 (54.8)	53 (61.6)
Clomiphene citrate (n, %)	15 (35.7)	21 (24.4)
IVF/ICSI (n, %)	4 (9.5)	12 (14.0)
Other (n, %)	0 (0)	0 (0)
Fasting insulin at inclusion (µIU/ml)	15.9 (9.6)	15.7 (11.7)
Fasting glucose at inclusion (mmol/l)	4.7 (0.5)	4.6 (0.4)
HOMA-IR at inclusion	3.3 (2.2)	3.2 (2.8)
2-h glucose (mmol/l)	5.6 (1.7)	5.5 (1.5)
Systolic blood pressure (mm Hg)	120 (13)	119 (12)
Diastolic blood pressure (mm Hg)	76 (18)	74 (9)
GDM at inclusion (n, %)	5 (12.5)	3 (4.2)

Abbreviations: BMI = body mass index, PCOS = polycystic ovary syndrome, PCO = polycystic ovaries, OA = oligo- and amennorhea, HA = hyperandrogenism, IVF = *in vitro* fertilization, ICSI = intracytoplasmic sperm injection, HOMA-IR = homeostatic model assessment of insulin resistance, GDM = gestational diabetes mellitus. Continuous variables are presented as mean (SD) and categorical variables as number (%) of participants.

For peer review only

d) Baseline characteristics of the participants using metformin both at conception and during pregnancy or no metformin at all

	Metformin at conception and metformin during pregnancy (n=42)	No metformin at conception and placebo during pregnancy (n=95)
Age (years)	30.4 (4.2)	29.0 (4.3)
Weight (kg)	83.3 (18.8)	79.0 (19.4)
BMI (kg/m^2)	29.5 (6.7)	28.6 (7.9)
PCOS phenotype	n=40	n=79
A (PCO+OA+HA) (n, %)	23 (57.5)	45 (57.0)
B (OA+HA) (n, %)	1 (2.5)	2 (2.5)
C (PCO+HA) (n, %)	5 (12.5)	9 (11.4)
D (PCO+OA) (n, %)	11 (27.5)	23 (29.1)
hyperandrogenic (n, %)	29 (72.5)	56 (70.9)
normo-androgenic (n, %)	11 (27.5)	23 (24.2)
Mode of conception $^{p=0.048}$	n=42	n=95
Spontaneous (n, %)	23 (54.8)	53 (55.8)
Clomiphene citrate (n, %)	15 (35.7)	19 (20.0)
IVF/ICSI (n, %)	4 (9.5)	23 (24.2)
Other (n, %)	0 (0)	0 (0)
Fasting insulin at inclusion (µIU/ml)	15.9 (9.6)	15.7 (12.1)
Fasting glucose at inclusion (mmol/l)	4.7 (0.5)	4.6 (0.5)
HOMA-IR at inclusion	3.3 (2.2)	3.2 (2.5)
2-h glucose (mmol/l)	5.6 (1.7)	5.6 (1.6)
Systolic blood pressure (mm Hg)	120 (13)	117 (12)
Diastolic blood pressure (mm Hg)	76 (18)	72 (9)
GDM at inclusion (n, %)	5 (12.5)	5 (6.3)

Abbreviations: BMI = body mass index, PCOS = polycystic ovary syndrome, PCO = polycystic ovaries, OA = oligo- and amennorhea, HA = hyperandrogenism, IVF = *in vitro* fertilization, ICSI = intracytoplasmic sperm injection, HOMA-IR = homeostatic model assessment of insulin resistance, GDM = gestational diabetes mellitus. Continuous variables are presented as mean (SD) and categorical variables as number (%) of participants.

For peer review only

e) Baseline characteristics of hyperandrogenic and normo-androgenic participants

	Hyperandrogenic PCOS phenotypes (n=164)	Normoandrogenic PCOS phenotypes (n=64)
Age (years)	29.5 (4.6)	28.9 (4.0)
Weight (kg) $^{p=0.034}$	81.8 (18.2)	76.1 (18.2)
BMI (kg/m^{2} $^{p=0.015}$	29.5 (6.9)	27.0 (7.0)
Mode of conception	n=164	n=64
Spontaneous (n, %)	94 (57.4)	32 (50.0)
Clomiphene citrate (n, %)	43 (26.2)	20 (31.2)
IVF/ICSI (n, %)	24 (14.6)	11 (17.2)
Other (n, %)	3 (1.8)	1 (1.6)
Fasting insulin at inclusion (µIU/ml) $^{p=0.029}$	16.5 (12.1)	13.0 (6.7)
Fasting glucose at inclusion (mmol/l)	4.6 (0.5)	4.6 (0.6)
HOMA-IR at inclusion $^{p=0.034}$	3.4 (2.7)	2.7 (1.5)
2-h glucose (mmol/l)	5.6 (1.6)	5.1 (1.3)
Systolic blood pressure (mm Hg)	118 (12)	119 (11)
Diastolic blood pressure (mm Hg)	74 (13)	73 (9)
GDM at inclusion (n, %)	16 (9.8)	5 (6.3)

Abbreviations: BMI = body mass index, IVF = *in vitro* fertilization, ICSI = intracytoplasmic sperm injection, HOMA-IR = homeostatic model assessment of insulin resistance, GDM = gestational diabetes mellitus. Continuous variables are presented as mean (SD) and categorical variables as number (%) of participants.

For peer review only

Supplementary Table 2. Pregnancy complications in women with PCOS according to treatment with metformin or placebo during pregnancy

	Metformin (n=128)	Placebo (n=136)
Pregnancy-induced hypertension (n, %)	7 (5.5)	8 (5.9)
Preeclampsia (n, %)	10 (7.8)	5 (3.7)
Late miscarriage/preterm delivery (n, %)	4 (3.1)	16 (11.8)

Data presented as number (%) of participants.

For peer review only

Supplementary Table 3. Correlations between PROK1 level at pregnancy week 19 and BMI, HOMA-IR, fasting insulin, testosterone and androstenedione levels at inclusion and at pregnancy week 19.

	r-value	p-value	r-value	p-value
	metformin group		placebo group	
BMI at inclusion	-0.091	0.308	-0.013	0.881
BMI at week 19	-0.112	0.207	0.022	0.800
Fasting insulin at inclusion	0.097	0.312	0.017	0.856
Fasting insulin at week 19	0.075	0.432	-0.044	0.636
HOMA-IR at inclusion	0.090	0.345	0.023	0.809
HOMA-IR at week 19	0.085	0.377	-0.065	0.494
Testosterone at inclusion	0.050	0.602	0.119	0.205
Testosterone at week 19	0.123	0.203	-0.057	0.544
Androstenedione at inclusion	0.018	0.850	0.089	0.346
Androstenedione at week 19	0.087	0.308	-0.079	0.881

Abbreviations: BMI = body mass index, HOMA-IR = homeostatic model assessment of insulin resistance

For peer review only

Supplementary material

All participants in the Pilot and PregMet 1 studies met the Rotterdam criteria (retrospectively evaluated in the Pilot study, as it was conducted prior to the establishment of the Rotterdam criteria), additional inclusion criteria were: age 18-45 years, gestational age between weeks 5-12, and a singleton viable fetus shown on ultrasonography. Exclusion criteria were alanine aminotransferase higher than 90 nmol/L, serum creatinine higher than 139 mmol/L, known alcohol abuse, previously diagnosed diabetes mellitus or fasting serum glucose higher than at inclusion, treatment with oral glucocorticoids, or use of drugs known to interfere with metformin. Randomization to metformin or placebo was stratified according to metformin use at conception. All participants were assigned in a double-blind manner either to metformin or placebo group. The participants received 850 mg (Pilot study) or 1000 mg (PregMet 1 study) metformin or placebo twice daily. Women who used metformin at conception had a "wash-out" period of ≥7 days before inclusion. All the participants received a 1mg tablet of folate daily, and one daily multivitamin tablet containing: vitamin A 800 mg, vitamin B_1 1.4 mg, vitamin B_2 1.6 mg, vitamin B_6 2 mg, vitamin B_{12} 1 mg, folic acid 200 mg, niacin 18 mg, pantotenic acid 6 mg, vitamin C 60 mg, vitamin D 5 mg, vitamin E 10 mg, Fe^{2+} 14 mg, Zn^{+} 15 mg, Cu^{2+} 2 mg, iodine 150 mg, Mn^{2+} 2.5 mg, Cr^{+} 50 mg, and Se^{+} 50 mg (Vitaplex^{W}; Alpharma AS, Norway).

Gestational diabetes mellitus was diagnosed as fasting plasma glucose higher than 7 mmol/L and/or 2-h serum glucose higher than 7.8 mmol/L after an OGTT (75 g glucose dissolved in 300 ml water). Pregnancy-induced hypertension was diagnosed as blood pressure of 140/90 mmHg or higher, measured after 10 minutes rest at least at two occasions. Preeclampsia was diagnosed as blood pressure of 140/90 mmHg or higher, measured on two occasions after pregnancy week 20 and albuminuria of at least 2 dipsticks on one occasion or 1 dipstick on two occasions. Late miscarriage was defined as pregnancy loss between week 13 and week 22 and 6 days, preterm delivery was defined as delivery between week 23 and week 36 and 6 days, including spontaneous deliveries, induced vaginal deliveries, and operative deliveries for medical indications.

For peer review only
